# Cryopreservation of aldehyde-fixed whole brains

**DOI:** 10.1371/journal.pone.0344932

**Published:** 2026-08-24

**Authors:** Macy Garrood, Alicia Keberle, Andria Slaughter, Allison Sowa, Emma L. Thorn, Claudia De Sanctis, Kurt Farrell, John F. Crary, Andrew T. McKenzie

**Affiliations:** 1 Apex Neuroscience, Salem, Oregon, United States of America; 2 Microscopy and Advanced Bioimaging Core, Icahn School of Medicine at Mount Sinai, New York, New York, United States of America; 3 Friedman Brain Institute, Departments of Pathology, Neuroscience, and Artificial Intelligence & Human Health, Icahn School of Medicine at Mount Sinai, New York, New York, United States of America; 4 Neuropathology Brain Bank & Research Core and Ronald M. Loeb Center for Alzheimer’s Disease, Icahn School of Medicine at Mount Sinai, New York, New York, United States of America; The Islamic University, IRAQ

## Abstract

Long-term storage of aldehyde-fixed brain tissue is commonly performed in the fluid state. This has the potential to maintain morphology for many decades, but has been found to cause progressive loss of antigenicity over time for some biomolecules, motivating interest in alternative long-term preservation strategies, such as cryopreservation. While cryoprotection and subzero storage has been successfully used for brain tissue sections or blocks, methods for preserving whole brains using this approach have not been widely characterized. Here we present a protocol for preserving fixed whole brains using graded immersion cryoprotection followed by subzero temperature storage. We refer to this general strategy – aldehyde fixation followed by cryoprotectant loading and subzero storage – as aldehyde-based cryopreservation (ABC). Our method uses a gradual ramp-up of the osmotic concentration of cryoprotectants, leading to a final solution containing 50% (v/v) ethylene glycol and 30% (w/v) sucrose in fixative. We used CT imaging to track cryoprotectant penetration, finding that approximately 9 months is required for the CT signal to stabilize throughout whole human brains. In our initial validation experiment, insufficient equilibration time prior to freezing led to ice crystal artifacts in the white matter. After refining the protocol to allow adequate diffusion time, light and electron microscopy showed preserved cellular architecture and ultrastructure. Our approach may be valuable for laboratories seeking a method for long-term subzero storage of fixed whole brain specimens.

## Introduction

Many thousands of human brains are banked around the world yearly with the goal of future research study. Banked brains often contain rare pathologies, unique genetic variants, or specific disease states that cannot be replicated. Additionally, many animal model specimens, such as those from long-term studies or unique experimental conditions, may require robust long-term preservation. For this reason, it is critical to develop preservation methods that can maintain both structural and molecular features of brain tissue over extended time periods. Fluid preservation of aldehyde-fixed brain tissue at room or refrigerator temperature is a widely used method, due to its simplicity and ability to maintain morphological features for years or decades [1]. However, studies have shown progressive loss of antigenicity during prolonged storage, as well as other potential biochemical changes, which could impact some types of future research applications. Although cryopreservation has proven successful for preserving molecular aspects of brain tissue sections or blocks, this technique can cause damage to cellular structures, in part due to the formation of ice crystals during freezing [2]. As a result, there is a critical need to develop improved methods for long-term preservation that can both maintain structural integrity and minimize biomolecular alterations of whole brain specimens. Developing and validating such methods requires addressing several practical and technical questions, which is the focus of the present study.

One alternative approach is to combine aldehyde fixation and cryopreservation. After the initial aldehyde fixation, the tissue can be immersed in cryoprotective agents and then cryopreserved. This approach prevents ice damage and the resulting morphological damage from unprotected cryopreservation, while also minimizing the loss of antigenicity associated with long-term storage in aldehyde solutions. We use the term aldehyde-based cryopreservation (ABC) to describe this general strategy, in which tissue that is already aldehyde fixed is subsequently cryoprotected and stored at subzero temperatures.

While numerous previous studies have applied this approach to sections or blocks of fixed brain tissue and reported preservation of both morphology and protein immunoreactivity for extended periods [3–11], its application to whole brains or large tissue segments has been less thoroughly characterized [12–15]. Two practical questions in particular are incompletely resolved. First, how long does cryoprotectant immersion require for diffusion to reach equilibration throughout an intact brain, which determines the waiting period before subzero storage? Second, does the cryoprotectant loading, subzero cooldown, warming, and cryoprotectant unloading process itself introduce structural alterations that are visible by microscopy? Although prior studies using these protocols have not identified significant alterations, replication would strengthen confidence in the approach.

To address these two questions, we describe a protocol for whole brain cryoprotectant immersion following aldehyde fixation and present two validation studies. First, using CT imaging of whole human brains, we characterize the time course of cryoprotectant penetration throughout the brain volume, providing a measure for when the tissue has reached sufficient equilibration for transfer to subzero storage. Second, through histological analysis of biopsy samples, we assess whether the cryoprotectant loading, freezer storage, and unloading steps introduce microscopic alterations. Our findings provide a diffusion timeline and structural validation for laboratories seeking to preserve intact brains for future research applications.

## Materials and methods

### Anatomical donation procedures

Dog brains were obtained postmortem after euthanasia by a licensed veterinarian independent of our organization, with owner consent for research use. Pig brains were sourced as animal byproducts from regulated agricultural facilities. All animal tissue was obtained postmortem from animals that died for reasons unrelated to this study; no live animals were used, and therefore IACUC approval was not required. Human whole body donations were performed by a partner organization operating under Oregon Health Authority regulations. The Apex Neuroscience Brain and Tissue Bank operates under an exemption determination from the Pearl Institutional Review Board (Pearl IRB ID #2023-0260).

### Initial tissue fixation

Human and pig brains were removed from the skull following standard procedures and immersion fixed in 10% neutral buffered formalin (NBF; Azer Scientific NBF55G) at 4°C for at least one month prior to further processing (Table 1) [16,17]. The dog brains were perfusion fixed *in situ* with 10% NBF: donor #65 via transcardial cannulation approximately 90 minutes after euthanasia, and donor #177 via bilateral carotid artery injection approximately 20 minutes after euthanasia. Both were subsequently immersion fixed in 10% NBF at 4°C for at least one month prior to further processing.

**Table 1:** Reagents used in this study.

Chemical	Supplier	Identifier
10% Neutral Buffered Formalin	Azer Scientific	NBF55G
50% Glutaraldehyde	Thermo Fisher	A10500.0E
Ethylene glycol	CQ Concepts	r-671–5
Sucrose	Lab Alley	SUCGA-500G
Polyvinylpyrrolidone (Average Molecular Weight 40,000)	Sigma	PVP40-100G

### Cryoprotectant penetration kinetics study

Three intact human brains were used to characterize the penetration kinetics of the cryoprotectant: a 71-year-old woman (donor #54, postmortem interval (PMI) of 117 hours), a 90 + -year-old man (donor #137, PMI 47 hours), and a 75-year-old man (donor #180, PMI 46 hours). These represented a convenience sample of brains donated to the Apex Neuroscience Brain and Tissue Bank, with no specific exclusion criteria.

After fixation in 10% NBF for at least one month, whole brains were transferred through a series of cryoprotectant solutions. The brains were maintained at the temperature of 4°C, except for one week when they were left at room temperature. Step 1 was 10% ethylene glycol (v/v; 400 mL) added to the solution of 10% NBF (3.6 L) for a total of 4 L of solution. In step 2, 1143 mL of ethylene glycol was added to this solution, reaching a total of 30% ethylene glycol (v/v) in 5 L of fluid. In step 3 (the final solution), a new solution was made, with 50% ethylene glycol (v/v; 2500 mL), 30% sucrose (w/v; 1500 g), and 1% PVP-40 (w/v; 50 g), with the remainder of the solution being 10% NBF. The 30% sucrose and 1% PVP was dissolved in the ethylene glycol at a slightly elevated temperature. Each intermediate step was maintained for at least one month before proceeding to the next concentration. Solutions were not replaced or replenished within a given step. The brains remained in the final solution until equilibration was confirmed by CT imaging. Given a human brain volume of approximately 1.3 L, the volume of the bath relative to the tissue volume of the brain was approximately 3:1 in step 1 (4 L solution) and 4:1 in steps 2 and 3 (5 L solution). CT scans were performed using the OmniTom Elite (Neurologica, Danvers, MA), a 16-slice portable CT scanner. Images were viewed with the Osimis Web Viewer. Baseline scans were obtained after fixation, with sequential scans after each cryoprotectant step. Equilibration was determined when the Hounsfield Unit values stabilized throughout the brain.

To assess macroscopic dimensional changes during cryoprotectant loading, we measured the brain width on coronal CT images at each imaging time point. For each scan, we identified the most anterior coronal slice in which the frontal horns of the lateral ventricles were clearly visible. The maximum transverse brain width was then measured at this level using the measurement tool in Orthanc Explorer 2. The frontal horns were chosen as the reference landmark as they are well-defined structures that are identifiable across serial scans. Because this approach depends on consistent identification of an anatomical landmark across time points, we performed a second analysis using sagittal CT slices, which captures the orthogonal anteroposterior dimension and does not require identification of an anatomical landmark. For each scan, we identified the sagittal slice containing the greatest anteroposterior brain length and measured the length at that level. In one of the CT scans, the scan was incomplete such that the posterior aspect of the brain was not fully visible, and in this case, the length was estimated based on extrapolation from the visible parts of the brain.

### Histological validation study

Biopsy samples (approximately 1.5 x 1.5 x 1 cm) from two perfusion-fixed dog brains were used to assess whether cryoprotectant loading, freezer storage, and unloading introduce histological alterations. All solution changes for biopsy samples were performed in 20 mL conical tubes containing 10 mL of solution per sample, yielding a bath-to-tissue volume ratio of approximately 4.4:1 (10 mL solution to ~2.25 mL tissue). We performed an initial experiment (donor #65) and, based on those results, developed a refined protocol that was tested in a second experiment (donor #177).

#### Initial protocol (donor #65).

Samples were collected from the sensorimotor cortex, thalamus, and subcortical white matter. For each region, samples were divided into cryopreserved and non-cryopreserved groups. Non-cryopreserved control samples were maintained in 10% NBF at 4°C during the cryoprotectant ramp up and down.

Cryopreserved samples were transferred through cryoprotectant solutions at 4°C. On day 1, samples were placed in 10% ethylene glycol (v/v) in 10% NBF. On day 2, samples were transferred to 30% ethylene glycol (v/v) in 10% NBF. On day 3, samples were transferred to the final solution: 50% ethylene glycol (v/v), 30% sucrose (w/v), and 1% PVP-40 (w/v), with the remainder being 10% NBF. The 30% sucrose and 1% PVP was dissolved in the ethylene glycol at a slightly elevated temperature before adding the NBF. After 3 days for equilibration, samples were transferred to −20°C for 3 days.

For cryoprotectant unloading, samples were rewarmed at 4°C for 24 hours, then transferred through decreasing concentrations: 30% ethylene glycol (v/v) in 10% NBF for 5 days, 10% ethylene glycol (v/v) in 10% NBF for 7 days, and finally 10% NBF alone. At the end of this, a 50% glutaraldehyde stock solution (2% v/v) was added to both cryopreserved and control samples for one day prior to processing for electron microscopy, resulting in a final glutaraldehyde concentration of 1%.

#### Refined protocol (donor #177).

Based on the ultrastructural artifacts we observed in white matter samples from the initial protocol, we developed a refined protocol with four main modifications: (1) a slower loading protocol with more time for equilibration prior to moving the samples to the freezer, (2) a more gradual unloading protocol with smaller concentration decrements, (3) inclusion of glutaraldehyde earlier in the loading and unloading steps, and (4) substitution of 0.01M PBS for most of the 10% NBF used as the bulk aqueous vehicle in the initial protocol. The rationale for the addition of glutaraldehyde was to reduce the osmotic reactivity of the tissue and thereby help cells to maintain their shape when exposed to and removed from hyperosmotic cryoprotectant solutions. The timing of the unloading schedule was chosen as a conservative reduction in step size relative to the initial protocol. The substitution of PBS for most of the NBF was made to reduce the total formaldehyde load through the loading, storage, and unloading processes. We judged formaldehyde unnecessary at the original concentration due to the effect of adding glutaraldehyde earlier in this protocol than in other protocols, and maintaining glutaraldehyde throughout the solution changes.

Samples were collected from parietal and temporal cortex and nearby subcortical white matter. For each region, samples were divided into cryopreserved and non-cryopreserved groups. Non-cryopreserved control samples were maintained in 10% NBF with 1% glutaraldehyde at 4°C, to match the glutaraldehyde exposure of the cryopreserved group.

Cryopreserved samples were transferred through cryoprotectant solutions at 4°C. On day 1, samples were placed in 10% ethylene glycol (v/v) in 10% NBF. On day 5, samples were transferred to 30% ethylene glycol (v/v) in 10% NBF, with 2% (v/v) of a 50% glutaraldehyde stock solution, for a glutaraldehyde concentration of 1%. Glutaraldehyde was first introduced at this day 5 loading step and was maintained in all subsequent loading and unloading solutions. On day 9, samples were transferred to the final solution, which contained 50% ethylene glycol (v/v), 30% sucrose (w/v), and 2% (v/v) of a 50% glutaraldehyde stock solution. The remaining 48% (v/v) of the nominal formulation consisted of 10% NBF (10% v/v of the total, for a final concentration of 1% NBF) and 0.01M PBS (38% v/v of the total). In this case, the solutions were prepared by first dissolving sucrose in the PBS component using a heating plate, then once it had cooled, adding ethylene glycol, NBF, and glutaraldehyde to prepare the final solution. Notably, the additional volume contributed by the dissolved sucrose after adding it to PBS was not accounted for, so these percentages should be considered nominal concentrations, because they are based on the intended formulation rather than the measured final solution volume. The same preparation order was used for the unloading solutions (Table 2). After 3 days for equilibration, the samples were transferred to −20°C for 7 days.

**Table 2:** Cryoprotectant unloading steps for the refined protocol (donor #177). The sucrose was completely dissolved in the PBS before the specified volume of the PBS was added to the preparation. However, the additional volume of the sucrose after adding it to PBS was not accounted for, so these should be considered nominal concentrations, and the actual final concentrations of the solution components were slightly lower. Glut: Glutaraldehyde. PBS: Phosphate buffered saline. NBF: Neutral buffered formalin.

Day	Ethylene glycol (v/v)	Sucrose (w/v)	0.01M PBS (v/v)	10% NBF (v/v)	50% Glut. (v/v)
1	40%	25%	30%	28%	2%
4	30%	20%	23%	45%	2%
7	20%	15%	18%	60%	2%
11	10%	10%	13%	75%	2%
15	5%	5%	8%	85%	2%
18	0%	0%	0%	98%	2%

For cryoprotectant unloading, samples were rewarmed at 4°C for 24 hours, then transferred through a gradual 18-day protocol with decreasing concentrations of ethylene glycol and sucrose (Table 2). Glutaraldehyde was maintained throughout all unloading steps. Following unloading, samples remained in 10% NBF with 1% glutaraldehyde at 4°C for several months until processing for electron microscopy. As with the final loading solution, the additional volume contributed by dissolved sucrose was not accounted for. Therefore, the percentages listed here should be regarded as nominal concentrations.

### Histological methods

For light microscopy, brain tissue was placed into cassettes for processing and embedded in paraffin. Paraffin-embedded brain sections 6 μm thick were baked, deparaffinized, and stained for Hematoxylin and Eosin (H&E). Digital images of the stained sections were captured at 40X as whole slide images (WSIs) using an Aperio GT450 high-resolution scanner (Leica Biosystems).

For electron microscopy on the initial protocol, we followed a previously described protocol [18]. Briefly, the samples were cut into 1 mm^3^ pieces and placed in mPrep/s capsules. Using an ASP-2000, the samples were rinsed in 0.1M sodium cacodylate buffer, stained with 2% osmium tetroxide in 0.1M sodium cacodylate, then 2.5% w/v potassium ferricyanide in 0.1M sodium cacodylate, rinsed in water, immersed in 1% w/v aqueous thiocarbohydrazide, rinsed in water, stained with 2% aqueous osmium tetroxide, rinsed in water, stained with 1% uranyl acetate, rinsed in water, and finally stained with lead aspartate. After rinsing in water, the samples were dehydrated in an ascending acetone series, infiltrated with resin overnight, and polymerized in a 60°C oven. Once polymerized, the samples were sectioned at 70 nm thickness. Sections were placed on a grid, stained with uranyl acetate and lead citrate, and imaged at 80 kV in an FEI Tecnai T12 transmission electron microscope equipped with an AMT Nanosprint12 camera.

For electron microscopy on the refined protocol, tissue samples were processed as previously described [19]. Briefly, the tissue was processed using an adapted NCMIR protocol for enhanced contrast. This included sequential treatments with tannic acid, reduced osmium, thiocarbohydrazide, osmium, and uranyl acetate at room temperature, followed by lead aspartate staining at 60°C. Samples were dehydrated through graded ethanol, infiltrated with Embed 812 epoxy resin (EMS), and polymerized for 72 hours at 60°C. Ultrathin sections (70 nm) were cut using a Leica UC7 ultramicrotome and collected on nickel slot grids. Images were acquired on a HT7500 transmission electron microscope (Hitachi High-Technologies, Tokyo, Japan) using an AMT NanoSprint12 12-megapixel CMOS TEM Camera System, with minimal contrast adjustments applied during acquisition.

Two raters (M.G. and A.S.), blinded to experimental condition, independently scored each electron micrograph of the refined protocol for ultrastructural preservation quality using a subjective 5-point scale (1 = best preservation, 5 = worst preservation). Scoring was performed on the images from both grey matter (specimens c7–c9, n7–n9) and white matter (specimens c10–c12, n10–n12) samples, with three specimens per condition per region. A total of 83 cryopreserved images (37 grey matter, 46 white matter) and 83 non-cryopreserved control images (38 grey matter, 45 white matter) were scored. All electron microscopy imaging was performed at core facilities, and all raw image data that was generated by them has been made publicly available (see Data Availability Statement). The two raters developed the 1–5 scale together prior to blinded scoring. The scoring rubric was not pre-registered. The interrater reliability for these grades was calculated using the intraclass correlation coefficient (ICC), applying a model with agreement estimation, single unit of analysis, and two-way random-effects. The ICC values were interpreted using previously established guidelines [20]. For analysis, rater scores were averaged (arithmetic mean) to produce a single score per image. These image-level scores were then averaged within each sample to yield one arithmetic mean score per sample. Differences between the cryopreserved and non-cryopreserved groups were assessed using the Wilcoxon rank-sum test due to the small sample size (n = 6 per group). Effect sizes are reported as the Hodges–Lehmann shift estimate with its associated 95% confidence interval (obtained from the Wilcoxon rank-sum test) and the Mann–Whitney area under the curve (AUC), defined as the probability that a randomly chosen cryopreserved specimen scores higher than a randomly chosen control, with a percentile 95% confidence interval based on 10,000 bootstrap replicates.

### Use of artificial intelligence tools

The authors used Claude (Anthropic) during manuscript preparation. The tool was used for two purposes. First, for writing and debugging R code for electron microscopy data analysis. Second, for editing the language of the manuscript to improve clarity and readability. All code generated with AI assistance was reviewed by the authors and tested against expected outputs before inclusion. All language edits were reviewed by the authors to ensure scientific accuracy was preserved. The authors take full responsibility for the accuracy and integrity of the entirety of the final manuscript.

## Results

*Preliminary observations on cryoprotectant selection* A wide variety of cryoprotective agents (CPAs) or mixtures could potentially prevent ice crystal formation at a storage temperature of −20°C. Our goal was to identify a mixture that would not only prevent ice formation but also serve well to maintain tissue structure as a fluid preservative, especially in the case of a freezer failure. As an initial qualitative pilot test, we tested glycerol on a single pig brain specimen, which we reasoned would be desirable due to its long established history as a fluid preservation agent and its high viscosity [1]. However, we found that pig brain tissue exposed to a concentration of 90% (v/v) glycerol for two days at 4°C demonstrated significant surface browning. This phenomenon of glycerol-induced browning has been previously reported numerous times, for example in the context of embalming [21] and tissue clearing [22]. It is likely due to the Maillard reaction. While this browning does likely not impact a significant number of downstream applications, it could interfere with some analyses and was therefore deemed undesirable. This observation led us to explore alternative CPA additives that did not contain glycerol. We used ethylene glycol (50% v/v), sucrose (30% w/v), and PVP-40 (1% w/v), building on a previously designed preservation method that performed storage at −20°C [4]. We tested this storage method on a pig brain, finding that it did not cause rapid surface browning nor any obvious volumetric changes on gross visual examination. Furthermore, we found that CT scans could be used to track the increases in the concentration of ethylene glycol.

### Penetration rate and effect on volumes in human brains

**Fig 1:**
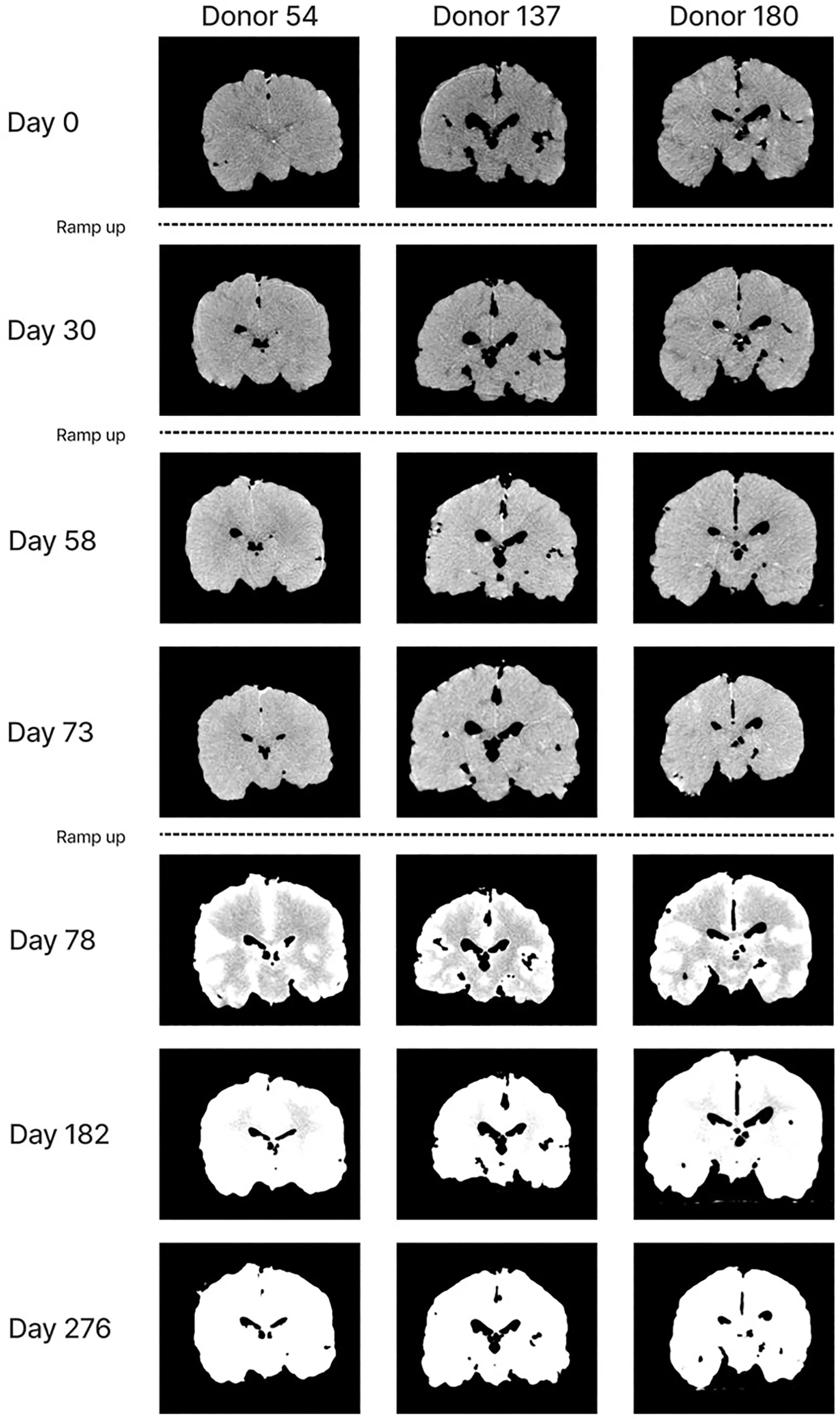
CT imaging of cryoprotectant penetration in whole human brains. Coronal CT sections from three donors (donor #54: 71-year-old female, postmortem interval (PMI) 117 hr; #137: 90 + -year-old male, PMI 47 hr; #180: 75-year-old male, PMI 46 hr) during stepwise cryoprotectant loading. Dashed lines indicate the concentration increases: 10% ethylene glycol (added on day 0), 30% ethylene glycol (added on day 30), and final solution containing 50% ethylene glycol, 30% sucrose, and 1% PVP-40 (added on day 73). Increasing brightness reflects rising Hounsfield Unit values as cryoprotectant diffuses inward. Note that grey matter equilibrates faster than white matter, with deep white matter regions showing delayed penetration. By day 276, the uniform signal intensity indicates equilibration throughout all of the brain.

For the first two cryoprotectant loading steps (10% and 30% ethylene glycol), we waited approximately one month between each concentration increase, which our CT scans revealed was not sufficient for full equilibration throughout the entire brain volume (Fig 1). However, we reasoned that brain-wide equilibration was not necessary at these intermediate steps, as it is only the outermost brain tissue that would be exposed to the higher concentration and thus the largest osmotic concentration difference. The more inner parts of the brain tissue would instead gradually increase in cryoprotectant concentration over time through diffusion, naturally preventing a large increase in osmotic concentration at any specific region. This approach allowed us to substantially decrease the waiting period before transferring the brain to subzero temperatures, making the protocol more feasible for implementation in brain banking facilities. For the final concentration step, we did wait for full equilibration as confirmed by CT imaging, with Hounsfield Unit values stabilizing between outer cortical and deep brain regions after 276 days. Notably, we found that white matter appeared to have substantially slower cryoprotectant diffusion kinetics than grey matter.

To assess for macroscopic dimension changes during cryoprotectant loading, we measured the maximum transverse brain width at the level of the frontal horns of the lateral ventricles on serial CT images. For donors #54 and #137, the brain width remained stable throughout the loading protocol, with no appreciable volumetric changes observed across all time points (S1 Data). Donor #180 similarly showed stable measurements for the majority of the time points, but then at one time point exhibited an abrupt increase of approximately 10% in the measured width, which persisted stably across the final four time points (S1 Data). One possible explanation is that this could be a measurement artifact. In particular, as the cryoprotectant solution entered the ventricular spaces, the increased contrast within the ventricles may have altered the anteroposterior level at which the frontal horns were first visualized on coronal sections, shifting the measurement plane posteriorly to where the brain is naturally wider. To investigate this further, we performed a second analysis measuring the greatest anteroposterior brain length on sagittal CT slices, which captures an orthogonal dimension and does not require landmark identification (S2 Data). The abrupt increase seen in donor #180 was not replicated, and no consistent directional change was observed across any of the three donors. This supports the interpretation that the observed increase on coronal slice analysis was a measurement artifact rather than true swelling.

After two months of freezer storage at −20°C, one of the brains was removed for examination. We found that there were no macroscopic signs of ice crystal formation. The brain was firm but not frozen, and was easily able to be sectioned for extracting a biopsy sample. There was a degree of surface browning that accumulated during the approximately 9-month cryoprotectant loading period at 4°C, though this was not as severe as that observed with 90% glycerol (S3 Data). The browning was primarily present on the surface, with a gradient in the outermost few millimeters of the tissue.

### Histological assessment of the initial protocol

**Fig 2:**
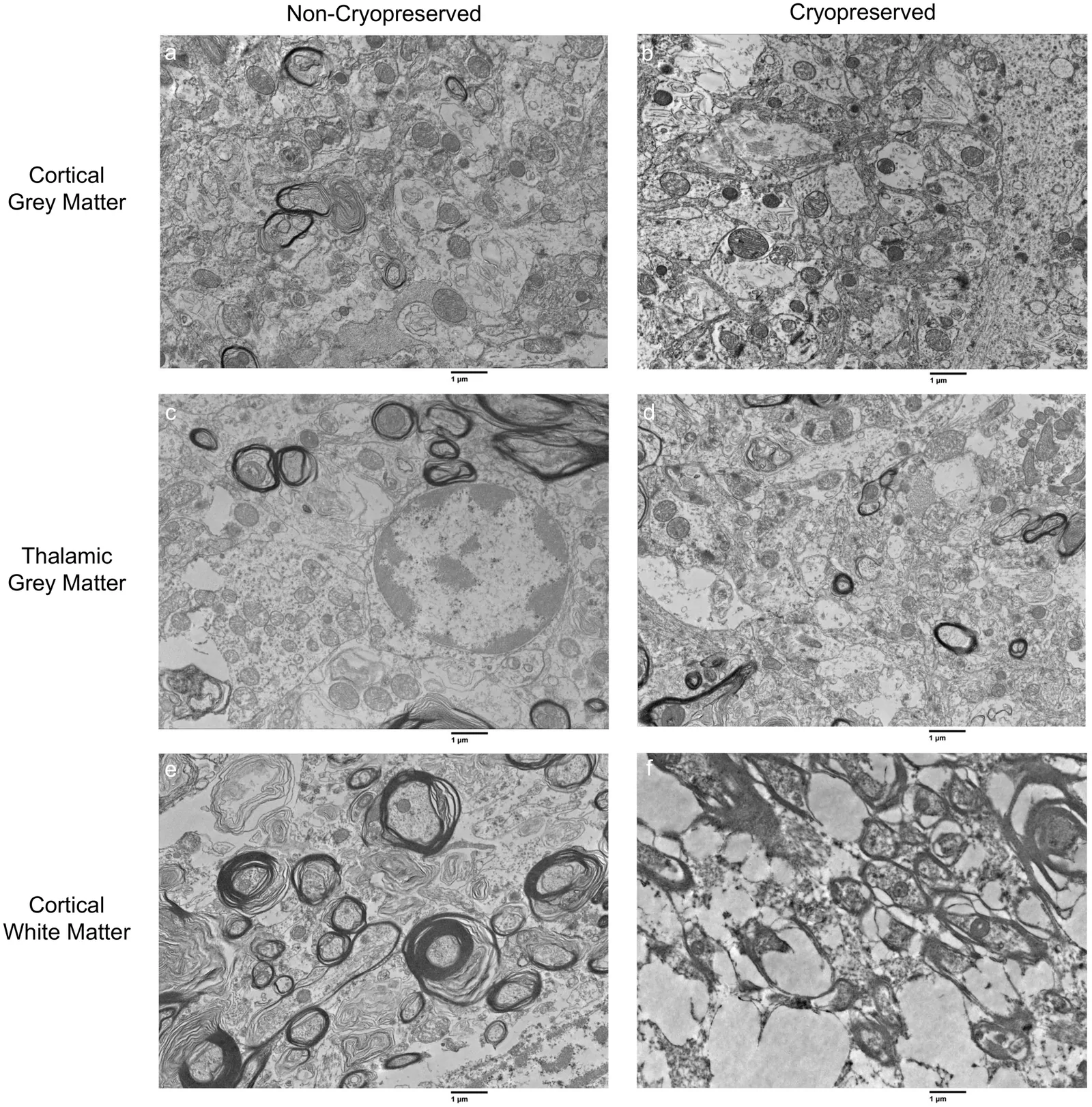
Electron microscopy of cryopreserved and non-cryopreserved brain tissue from the initial protocol. Tissue was prepared from donor #65 using osmium, potassium ferricyanide, thiocarbohydrazide, uranyl acetate, and lead aspartate, then post-stained on-section with uranyl acetate and lead citrate. Left column: non-cryopreserved controls maintained in fixative solution prior to embedding for electron microscopy. Right column: tissue after cryoprotectant loading, storage at −20°C, and unloading. **a**, **b**: Cortical grey matter. **c**, **d**: Thalamic grey matter. **e**, **f**: Subcortical white matter. Grey matter shows comparable preservation between conditions. Cryopreserved white matter (**f**) exhibits large void spaces with compressed surrounding tissue, consistent with ice crystal formation due to incomplete cryoprotectant penetration prior to the freezer storage. Scale bars: **a-f**: 1 μm.

In our initial experiment using tissue from donor #65, we assessed whether cryoprotectant loading, freezer storage at −20°C, and subsequent unloading would alter the tissue ultrastructure compared to non-cryopreserved controls maintained in fixative solution. We found that grey matter samples from the sensorimotor cortex and thalamus showed comparable preservation between cryopreserved and control conditions, with no differences in the structure of cellular membranes, and no obvious artifacts attributable to the cryopreservation process (Fig 2). Neither condition showed perfect ultrastructural preservation, but the alterations observed were consistent with expected postmortem changes rather than cryopreservation-induced damage [19,23].

However, subcortical white matter samples showed a different outcome. While non-cryopreserved white matter displayed myelinated axon profiles with expected postmortem changes such as delamination, cryopreserved white matter exhibited prominent artifacts characterized by large, sharply delineated void spaces that displaced and compressed the surrounding tissue (Fig 2). These irregular vacuolar structures ranged from sub-micron to approximately 10 μm in diameter and were distributed throughout the tissue, with intervening regions of compressed myelinated fibers between them. The morphology of these artifacts – i.e., discrete empty spaces with sharp boundaries and compressed surrounding tissue – is consistent with ice crystal formation [2]. We hypothesize that the 3-day cryoprotectant loading period was insufficient for complete equilibration in white matter, which has slower diffusion kinetics than grey matter. This would have left residual freezable water in white matter compartments, leading to ice formation during storage at −20°C. Ice crystal formation has previously been reported in fixed brain tissue when cryoprotectant infiltration is incomplete [12]. It is also possible that insufficient glutaraldehyde fixation prior to EM processing or osmotic stress during cryoprotectant unloading contributed to these artifacts. However, the sharp morphology of the artifacts and their restriction to the slower-diffusing white matter suggest that ice crystal formation is the sole cause.

### Histological assessment of the refined protocol

**Fig 3:**
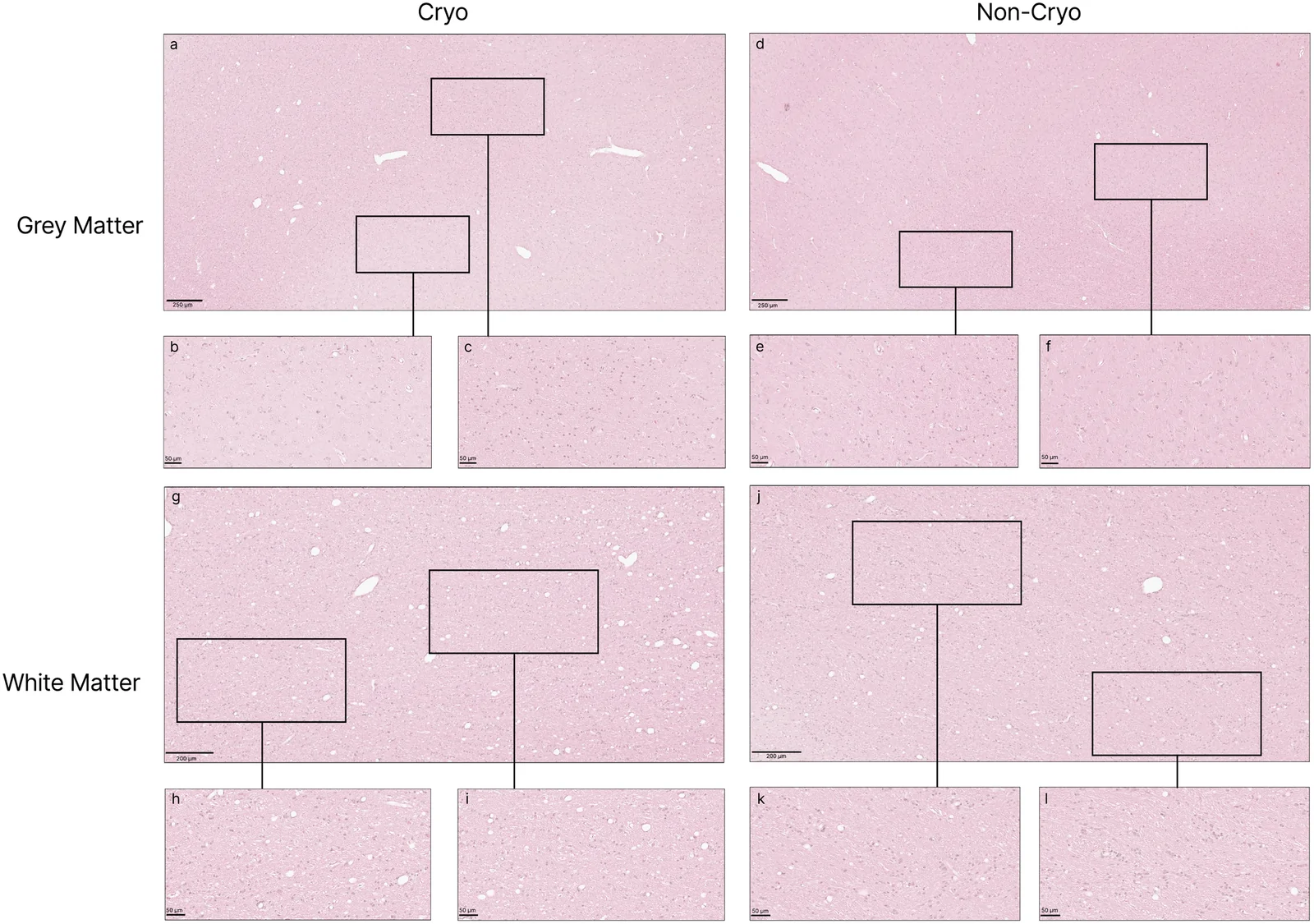
Representative light microscopy images comparing cryopreserved and non-cryopreserved canine brain tissue samples. H&E staining of cortical grey matter (**a-f**) and subcortical white matter (**g-l**). The left column shows cryopreserved samples (**a-c**: Donor #177 Cortex Sample #C1; **g-i**: Donor #177 White Matter Sample #C5) that underwent cryoprotectant loading, storage at −20 °C, and unloading. The right column shows non-cryopreserved controls (**d-f**: Donor #177 Cortex Sample #N1; **j-l**: Donor #177 White Matter Sample #N5) maintained in fixative throughout. Progressive magnification from overview to cellular detail shows that the tissue architecture is preserved in both conditions, with no observable differences in preservation quality between the cryopreserved and control samples in either grey matter or white matter. Scale bars: **a**, **d**: 250 μm; **g**, **j**: 200 μm; **b**, **c**, **e**, **f**, **h**, **i**, **k**, **l**: 50 μm.

We next evaluated tissue preservation using the refined cryopreservation protocol, which incorporated longer equilibration times, more gradual cryoprotectant unloading steps, and glutaraldehyde fixation. Light microscopy evaluation of both cryopreserved and non-cryopreserved samples using this protocol showed that the tissue architecture was preserved across all specimens (Fig 3). In both conditions, cell bodies, nuclei, and blood vessels remained clearly visible, and we observed no void spaces or tissue disruption patterns characteristic of ice crystal formation. This indicates that the cryoprotectant loading, freezer storage, and unloading protocol successfully maintained tissue integrity at the light microscopic level.

**Fig 4:**
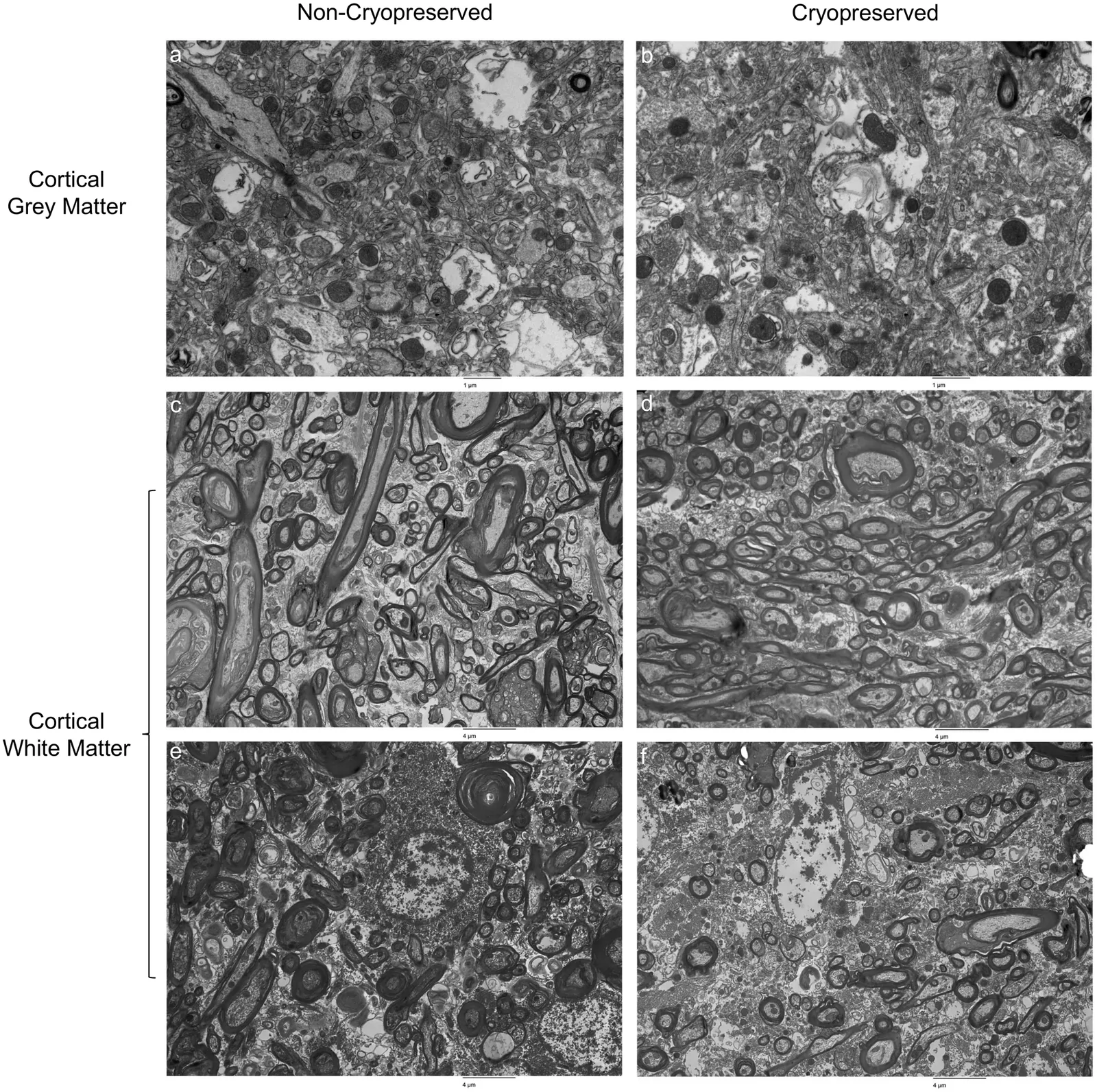
Representative electron micrographs of cryopreserved and non-cryopreserved brain tissue from the refined protocol. Tissue from donor #177 was prepared using an adapted NCMIR protocol with tannic acid, reduced osmium, thiocarbohydrazide, osmium, uranyl acetate, and lead aspartate. Left column: non-cryopreserved controls maintained in fixative solution prior to embedding for electron microscopy. Right column: tissue after cryoprotectant loading, storage at −20°C, rewarming, and cryoprotectant unloading. **a**, **b**: Cortical grey matter. **c**-**f**: Cortical white matter. Both grey matter and white matter show comparable preservation between cryopreserved and non-cryopreserved conditions, without the artifacts that were observed in the white matter in the initial protocol. Scale bars: **a**, **b**: 1 μm; **c**-**f**: 4 μm.

Electron microscopy of grey matter and white matter samples showed comparable preservation between the cryopreserved samples and non-cryopreserved controls (Fig 4). As with the initial experiment, neither condition showed perfect ultrastructural preservation, with expected postmortem alterations. Qualitatively, we found that there was no clear effect of the cryopreservation protocol. For example, there were no large void spaces observed in the white matter that were attributed to ice crystal artifacts, as were seen in the initial protocol. In order to assess this, two blinded raters independently scored each image for ultrastructural preservation quality on a subjective 5-point scale (1 = best, 5 = worst). Blinded ratings of the preservation quality in electron microscopy images showed good interrater reliability (ICC = 0.76, 95% CI: 0.69 to 0.82). There was no significant difference in the mean preservation scores between the six cryopreserved samples (mean = 2.10, median = 2.12) and the six non-cryopreserved control samples (mean = 2.54, median = 2.63; Wilcoxon rank-sum test, W = 11, p = 0.30; Hodges–Lehmann shift = −0.45, 95% CI: −1.41 to 0.23; Mann–Whitney AUC = 0.31, bootstrap 95% CI: 0.00 to 0.67; S4 Data). With n = 6 per group, the confidence intervals are wide and the study is powered only to detect large effects. These results suggest that the refined cryopreservation protocol preserves tissue throughout the cryoprotectant loading, freezer storage, and unloading process without introducing a large burden of ultrastructural artifacts.

## Discussion

In this study, we describe our protocol for whole-brain cryoprotectant immersion following aldehyde fixation, enabling long-term storage at −20°C. This is one implementation of the ABC approach to long-term preservation. Using CT imaging, we characterized the time course of cryoprotectant penetration in whole human brains, finding that with this protocol, approximately 9 months is required for full equilibration of the final preservation solution. In our histology studies, we found that cryoprotectant loading, freezer storage, and unloading did not introduce significant alterations to tissue architecture or ultrastructure, as long as the time for cryoprotectant loading was adequate. This method offers several advantages for brain banking applications, as it can be implemented using standard laboratory freezers, it keeps the tissue easily accessible for sampling, and it relies upon widely available, well-characterized cryoprotective agents.

### Cryoprotectant formulation

A well-established protocol in the literature for long-term freezer storage of fixed brain tissue, Watson et al. (1986), uses a mixture of 30% ethylene glycol, 30% sucrose, and 1% polyvinylpyrrolidone (PVP-40) [4]. Storage of fixed brain tissue under these conditions has been reported to maintain both tissue ultrastructure and antigenicity for multiple decades [4,8]. We modified this protocol in several ways to optimize it for our purposes.

First, we increased the concentration of ethylene glycol from 30% (v/v) to 50% (v/v). At this concentration, ethylene glycol alone is sufficient to ensure the complete prevention of ice formation, because any solution with a melting point below the storage temperature cannot freeze [24]. Raising the concentration of ethylene glycol further would lead to a risk of crystallization of ethylene glycol solutes.

Second, our final protocol uses fixative as a component of the solution. The rationale for this is twofold. First, we want to prevent the possibility of microbial growth during the approximately 9-month cryoprotectant diffusion period at refrigerator temperature, which is much longer than required for tissue sections. We note that the residual fixative within the tissue after the initial fixation would likely be sufficient for this purpose, so adding fixative to the cryoprotectant loading solutions is a more conservative approach and may not be necessary. Second, we want the solution to function as an effective long-term fluid preservative in its own right. This way, if circumstances arise wherein freezer storage becomes not possible, the tissue would remain adequately preserved in the fluid state at room or refrigerator temperature for the long-term without the need for any solution changes.

Third, although PVP-40 was included in the original formulation [4] and in our whole brain cryoprotectant kinetics study, we omitted it from the refined biopsy protocol and intend to omit it from future whole brain work as well. We found it impractical for our whole brain application, as it was difficult to prepare large volumes of solution containing it, because PVP dissolves so slowly. Since 50% ethylene glycol alone provides sufficient cryoprotection at −20°C, and the sucrose component contributes to solution viscosity, we determined that PVP was not essential for our purposes.

Notably, of the cryoprotectants we used, only ethylene glycol was loaded in a stepwise manner. Our reasoning is that ethylene glycol is the dominant contributor to the osmolarity of the final solution, so its gradual introduction is what primarily matters for mitigating osmotic stress during loading. Sucrose was introduced only at the final step, by which point the tissue is already loaded with a high concentration of ethylene glycol, such that the marginal osmotic contribution from adding sucrose would be secondary.

We maintained the same concentration of sucrose at 30% w/v as in the original protocol [4]. Sucrose will increase the viscosity of the solution, which is expected to slow the molecular motion of biomolecules and aid in long-term preservation in the fluid state [1]. Consistent with this, sucrose has been found to be an effective fluid preservative for heart valves stored at 4°C [25]. Additionally, ethylene glycol has been reported to be an effective agent for improving the preservation of fixed tissue in the fluid state [26,27].

### Loading and unloading of cryoprotectants

The extent to which gradual osmotic concentration changes are necessary when introducing cryoprotectants to aldehyde-fixed tissue is not yet entirely clear. Fixatives themselves are not thought to exert a substantial effective osmotic force, because they freely diffuse across cell membranes [28,29]. Some studies have reported that fixed tissue can still have osmotic sensitivity, although the ones we identified employed relatively short fixation times, such as 4 hours or less [30,31]. In contrast, one study found that rat brain tissue fixed with glutaraldehyde for 24 hours is entirely resistant to osmotic stress [32]. Other studies have also found that an adequate degree of aldehyde fixation prior to cryoprotection prevents cellular alterations due to osmotic stress [33–35].

Several studies using similar cryoprotection protocols for fixed brain tissue have employed an immediate transfer to the final cryoprotectant concentration without a gradual ramp-up [8,11]. For example, in the Watson et al. (1986) protocol, which is the primary inspiration for this method, cryoprotected fixed brain sections were placed immediately in the final cryoprotectant mixture [4]. They reported that the ultrastructure remained normal after washing, although they did note transient shrinkage of the tissue upon cryoprotectant removal that resolved within 30 minutes in PBS. Other protocols have used a single intermediate cryoprotectant ramp up step [12,13]. In contrast, our final protocol uses three ramp-up steps (10% ethylene glycol, 30% ethylene glycol, then the final solution). For whole brain protocols such as ours, the stepwise gradient is likely only relevant to the outermost surface of the brain tissue. However, because we do not wait for equilibration until the final step, which takes by far the longest time, our use of the slower ramp-up approach only adds a relatively small amount of time compared to the overall protocol. Future studies could determine whether this degree of caution regarding rapid osmotic concentration changes is necessary, or whether more rapid loading and unloading would produce equivalent results with less time and complexity. Based on our biopsy-scale results, we intend to include glutaraldehyde in the loading and unloading solutions for whole brains in our future research, which will at least help stabilize the tissue at the surface where cryoprotectant concentration changes occur most rapidly.

### Equilibration time

There is some previously reported data on how much time has been used for cryoprotectant immersion in brain tissue. One study reported that fixed monkey brain blocks equilibrated with 30% sucrose in 4–7 days, as determined by sinking [12]. Another study reported that perfusion-fixed elephant brains equilibrated in their cryoprotectant solution (30% glycerol, 30% ethylene glycol) in approximately 3 weeks [13].

In this study, our CT data demonstrate that the full cryoprotectant loading protocol we used requires approximately 9 months at 4°C to reach a uniform distribution throughout intact human brains, with the final concentration ramp up and equilibration requiring approximately 7 months. Performing the equilibration at room temperature would accelerate diffusion, but we chose to use refrigerator temperature to minimize any potential biochemical changes during the loading period. Our CT imaging also revealed that white matter equilibrates substantially slower than grey matter. White matter consists of densely packed myelinated axons, and the lipid-rich myelin sheath may impede the diffusion of water-soluble cryoprotectants. Estimating effective diffusion coefficients for white versus grey matter from these CT data is an important direction for future work and would aid in protocol planning. Several strategies could also potentially shorten the equilibration period in future protocols, including a modest elevation of loading temperature (which would need to be balanced against any resulting biochemical changes), stirring the bath to keep the cryoprotectant concentration uniform at the tissue surface, sectioning of the brain prior to loading to shorten diffusion path lengths at the cost of losing whole-brain integrity, or leveraging vascular pathways to accelerate the diffusion of cryoprotectant solutions. Empirical evaluation of these strategies is an important area for future work.

One important limitation of our use of CT imaging to assess the penetration rate is that it is a measure of the combined X-ray attenuation of all solutes. Our final solution contains both ethylene glycol (MW 62 g/mol) and sucrose (MW 342 g/mol), which will diffuse at different rates, with ethylene glycol diffusing faster due to its smaller size. Because our concentration of ethylene glycol alone provides sufficient cryoprotection at −20°C, and because it penetrates faster than sucrose, adequate ice prevention will still be assured by the time a uniform CT signal is reached, regardless of which solute contributes more to the CT signal.

Instead of using cryoprotectant immersion, another possibility would be to perfuse cryoprotectant immediately after perfusion fixation. This method can achieve a more rapid distribution of cryoprotectants throughout the brain when used at the time of death in ideal laboratory circumstances [35,36]. However, in the context of postmortem human brain banking, perfusion quality is unreliable due to agonal factors, the postmortem interval, and vascular pathology in aged or diseased brains [37,38]. In the cases in which perfusion quality is low, there will be osmotic damage due to the introduction of cryoprotectants without prior fixation, inadequate intracellular penetration of cryoprotectants in the absence of fixation, and/or ice damage in areas with poor perfusion [2,35,39]. Therefore, we reasoned that the ABC approach, while substantially slower because it relies on cryoprotectant immersion, is more reliable for achieving structural preservation across the diverse range of specimens encountered in brain banking.

### Long-term preservation

A key limitation of this study is that we did not test long-term storage effects. However, our approach closely parallels other protocols that have been shown to maintain both tissue morphology and protein immunoreactivity for years [11] or decades [8]. Theoretically, the Q10 temperature coefficient predicts that most chemical reactions will slow by a factor of approximately 2 for every 10°C decrease in temperature. However, empirical evidence suggests that preservation at subzero temperatures with cryoprotectants substantially exceeds these kinetic predictions. For example, one study examined unfixed cat brains cryoprotected with 15% glycerol and stored at −20°C for up to 7.25 years, finding that parts of the brain retained cellular structure [40, 41]. This contrasts sharply with unfixed brain tissue stored at 4°C, which generally shows rapid cellular degradation within days to weeks [23]. As another example, fixed brain sections stored in cryoprotectant at −20°C have been found to maintain immunoreactivity for over 20 years, whereas before this method was developed, sections stored in standard buffers at refrigerator temperature could maintain this type of antigenicity for only a few days [8].

Samples naturally preserved in permafrost at temperatures around −7 to −10°C demonstrate that subzero conditions can enable very long-term preservation of some biological features, with DNA recovered from specimens over 1 million years old [42] and collagen ultrastructure preserved for 40,000 years [43]. Our specimens differ mechanistically from permafrost in that they are preserved in a viscous fluid rather than frozen state, but taking the available evidence together, the combination of aldehyde crosslinking, cryoprotectants, and storage at −20°C is expected to support long-term preservation, potentially for decades. Another possibility with the use of the ABC approach is to store the tissue at a lower temperature, such as −40°C or −80°C. Whether lower temperatures would provide meaningful additional preservation benefit beyond −20°C is unclear, as −20°C storage with cryoprotectants already dramatically slows biochemical reactions relative to refrigerator temperatures, which has been reported to lead to minimal changes in antigenicity for decades [8]. However, some investigators may prefer storage at lower temperatures. Our 50% (v/v) ethylene glycol solution alone has been reported to have a freezing point of −38.3°C [24]. The sucrose in the solution would depress the freezing point further through colligative effects, but the magnitude is, to the best of our knowledge, not well characterized for this ternary system, nor in the context of cryopreserving brain tissue. Therefore, modifying the cryoprotectant formulation may be necessary for the use of lower temperature storage. One option would be to increase the total concentration of permeating cryoprotectants, for example by incorporating dimethyl sulfoxide (DMSO). DMSO and glycerol mixtures have previously been used together for immersion cryoprotection of fixed brain tissue blocks prior to freezing and sectioning [5,12]. Future studies could evaluate whether modified cryoprotectant formulations and lower temperature storage provide measurable benefits over the −20°C protocol described here.

Notably, standard fluid preservation of aldehyde-fixed brain tissue at room or refrigerator temperatures has also been found to maintain many morphological features for extended periods [1]. The primary documented benefit of subzero storage in cryoprotectants is the prevention of progressive antigenicity loss that has been observed for some epitopes during prolonged fixative storage [4,8]. The extent to which cryoprotection and subzero storage may provide additional preservation benefits beyond improved retention of antigenicity, such as reduced lipid oxidation, is an area for future investigation. Another consideration is that the required 9-month period for the full equilibration entails a duration of fluid storage where some degree of antigen masking would already be expected to have occurred. During long-term fluid preservation in fixative, the most commonly affected biomolecular class is proteins with fixation-sensitive epitopes, whereby progressive crosslinking renders antigens increasingly inaccessible over time, while other biomolecules such as a subset of lipids and small molecules may be extracted or chemically modified [1]. The effects have been found to be specific to the particular antigen rather than determined by subcellular localization, although there is limited data directly comparing susceptibility across subcellular compartments [1]. Because the decline in immunoreactivity for moderately fixation-sensitive antigens is a progressive process that continues to worsen even after the first year [1], stabilizing the tissue state after 9 months of cryoprotectant equilibration is thought to still offer a long-term advantage over indefinite storage at higher temperatures. The specific time-temperature tolerances of the protocol during freezer failure, and the corresponding effects on the tissue, have not been characterized empirically and are an area for future work.

### Limitations

This study has several additional limitations. First, our protocol evolved during the study, with the refined histological validation protocol incorporating changes prompted in part due to the artifacts we observed in the initial experiment.

Second, our sample sizes were small. This limits the statistical power and generalizability of our findings. Larger studies will be needed to confirm these results.

Third, we used donated dog tissue because it allowed for shorter postmortem intervals than we had available from human donations, providing better baseline ultrastructure for detecting any cryopreservation-induced artifacts. However, it is possible that species differences could affect the generalizability of our results. For example, differences in white matter density or myelin composition between species could influence cryoprotectant diffusion kinetics, osmotic responses, and ultrastructural preservation outcomes. On a related note, it is important to point out that ultrastructural preservation of whole human brains following the full ABC protocol has not yet been empirically demonstrated and is a clear priority for future research. For whole-brain applications, one practical approach would be to take biopsy samples for ultrastructural assessment rather than unloading the entire brain, given the long diffusion path lengths that would otherwise be involved. Although the human brain tissue in the present study was sectioned to confirm that it could be cleanly cut, no tissue from these brains was submitted for histological or ultrastructural evaluation, and doing so is a clear next step.

Fourth, we assessed only morphological preservation and did not evaluate biomolecular labeling outcomes such as immunoreactivity, which is the primary rationale for this approach over conventional room temperature or refrigerator storage. Future studies using other methods such as immunohistochemistry, RNA preservation analysis, and protein stability assays are primary areas for further validation of the molecular preservation benefits of this protocol.

Fifth, although our protocol closely parallels published methods that have demonstrated multi-decade preservation of fixed brain sections at −20°C [8], our freezer storage experiment lasted only two months. Further empirical validation of long-term storage in whole brains is an important area for future research.

Sixth, in this study, the cryoprotectant solutions were not replenished within each loading step. As cryoprotectants diffuse from the bath into the tissue, the bath concentration will decrease over time, with the magnitude of this dilution depending on the bath-to-tissue solvent ratio and the duration of the step. This effect is most relevant for the final equilibration step, which lasted approximately 7 months. The resulting bath concentration at equilibrium would therefore be somewhat lower than the nominal starting concentration. Future studies could address this by periodically replacing the solution to maintain the nominal concentration throughout the equilibration period, and monitoring it via refractive index measurements.

Seventh, in the refined protocol for cryoprotectant loading and unloading of samples, the volume contribution of the dissolved sucrose in PBS was not accounted for when preparing the cryoprotectant solutions. As a result, the reported concentrations are nominal rather than true final concentrations, and the actual concentrations of the solution components were slightly lower than stated for those solutions.

Finally, our volumetric assessment used only two types of linear measurement on CT images, which have limited sensitivity for detecting small volume changes, and are susceptible to variability introduced by the positioning and tilt of the tissue on the scanner bed. Although this was not the primary focus of our study, future studies would benefit from larger sample sizes and more robust approaches to measuring volume changes, such as 3D reconstructions.

## Conclusions

We present our protocol for long-term storage of aldehyde-fixed whole brains at −20°C using ethylene glycol and sucrose cryoprotection. CT imaging demonstrates that approximately 9 months is required for our protocol to allow for cryoprotectant equilibration in whole human brains, with white matter equilibrating more slowly than grey matter. Our histological validation results, once optimized to allow adequate time for cryoprotectant diffusion into the white matter, corroborate existing data that this protocol maintains brain tissue ultrastructure through the process of cryoprotectant loading, brief storage, and cryoprotectant unloading. Additional mechanistic characterization and practical verification of preservation efficacy will be important next steps. Future work should also evaluate the long-term biomolecular preservation outcomes, determine whether the protocol can be simplified without compromising tissue quality, and explore its applicability to other organs and tissues.

## Supporting information

S1 Data Serial coronal slice CT measurements across donor brains. Maximum brain width measurements for donor IDs #54, #137, and #180 across 11 timepoints during cryoprotectant loading. For each time point, the width measurements were taken at the first coronal slice in which the frontal horns of the lateral ventricles were seen.
(PDF)

S2 Data Serial sagittal slice CT measurements across donor brains. Maximum brain anteroposterior distance measurements for donor IDs #54, #137, and #180 across 11 timepoints during cryoprotectant loading.
(PDF)

S3 Data Surface browning of a whole human brain after cryoprotectant loading. Photograph of a fixed whole human brain (donor ID #137) following the approximately 9-month graded cryoprotectant loading protocol at 4°C. A degree of surface browning is visible.
(TIFF)

S4 Data Per-specimen distributions of image-level preservation scores. Each point represents one electron microscopy image, plotted as the mean of two blinded raters’ scores on a 1–5 ordinal scale (1 = best preservation, 5 = worst). Points are jittered horizontally for visibility. Black horizontal bars indicate within-specimen medians. Specimens are grouped by region: c7–c9 and n7–n9 are grey matter (left panel); c10–c12 and n10–n12 are white matter (right panel). Blue = cryopreserved (n = 6 specimens, 83 images total); red = non-cryopreserved control (n = 6 specimens, 83 images total).
(PDF)

## Abbreviations

ABC: Aldehyde-based cryopreservation
CI: Confidence interval
CPA: Cryoprotective agent
CT: Computed tomography
DMSO: Dimethyl sulfoxide
EM: Electron microscopy
H&E: Hematoxylin and Eosin
ICC: Intraclass correlation coefficient
MW: Molecular weight
NBF: Neutral buffered formalin
PBS: Phosphate buffered saline
PMI: Postmortem interval
PVP: Polyvinylpyrrolidone
TEM: Transmission electron microscopy
WSI: Whole slide image
