# Supplementary material for: Cryopreservation of aldehyde-fixed whole brains
Source: PLoS One. 2026 Aug 24;21(8):e0344932. doi: 10.1371/journal.pone.0344932 (PMC13502592; doi:10.1371/journal.pone.0344932)
Supplement: Supplementary file 1 [file pone.0344932.s001.pdf]

CT scans

Format: Date:measurement mm @ frame number

<table>
<tr><th>DN 54</th><th>DN 137</th><th>DN 180</th></tr>
<tr>
<td>2/18/25:102.56 mm at 147
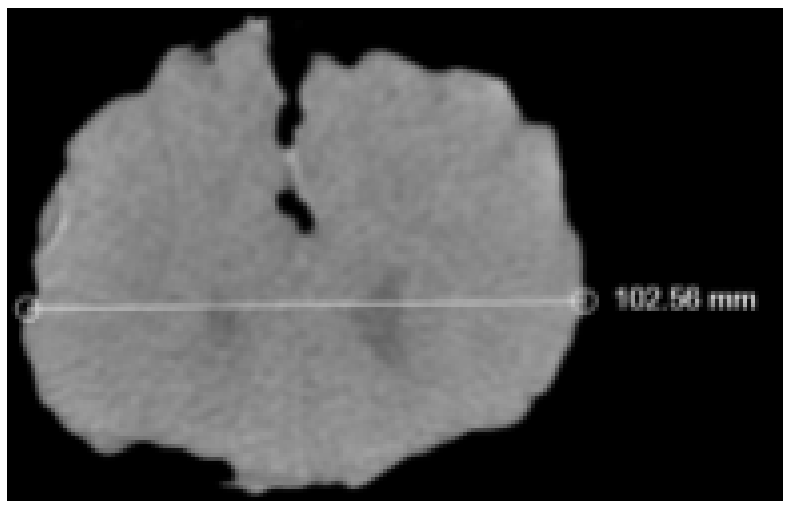</td>
<td>2/18/25:108.55 mm at 40
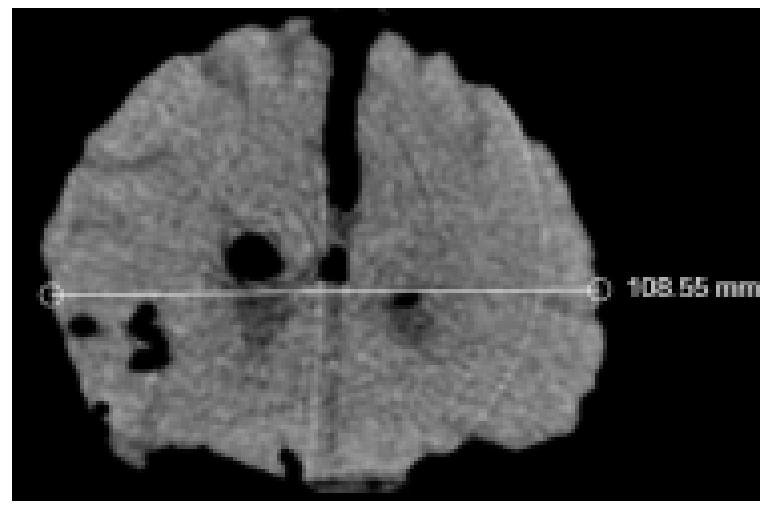</td>
<td>2/18/25:103.26 mm at 98
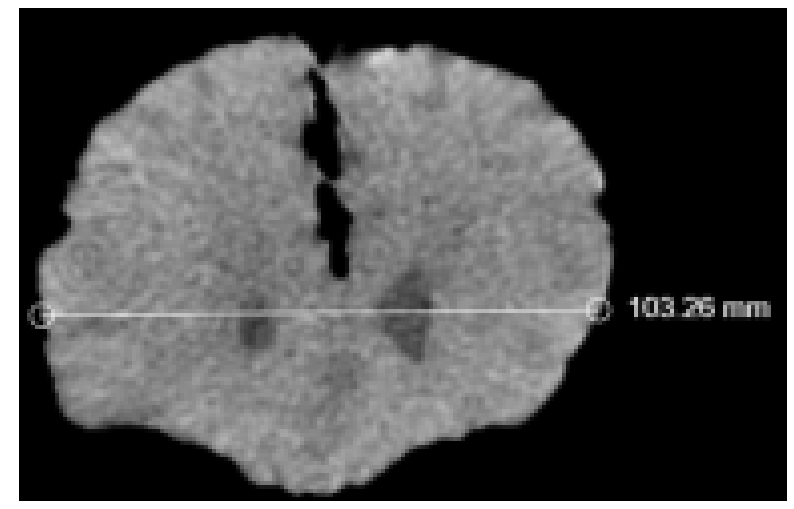</td>
</tr>
<tr>
<td>2/27/25:102.20 mm at 144
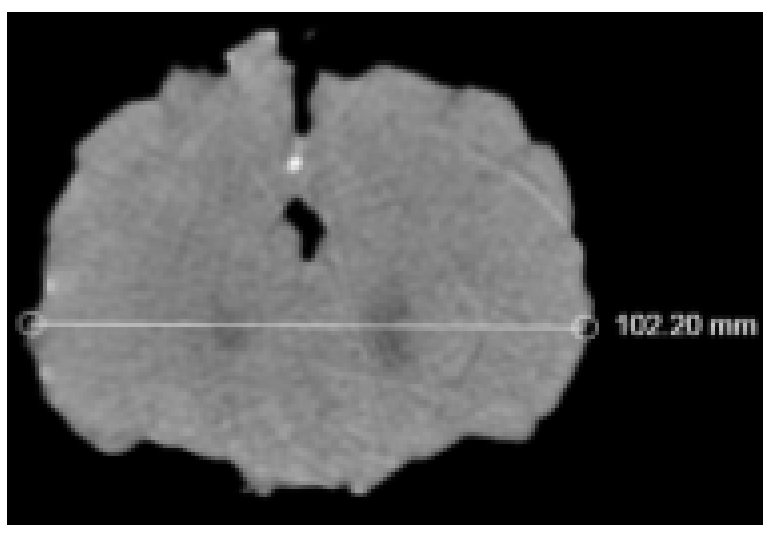</td>
<td>2/27/25:107.14 mm at 116
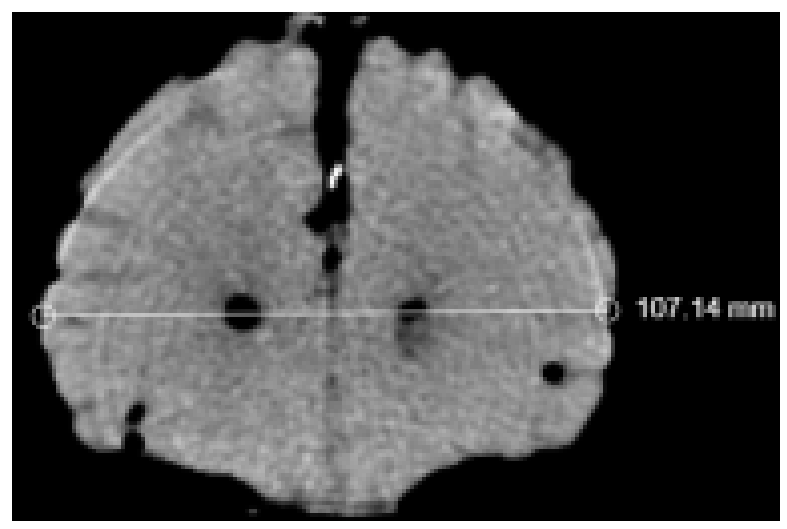</td>
<td>2/27/25:103.96 at 156
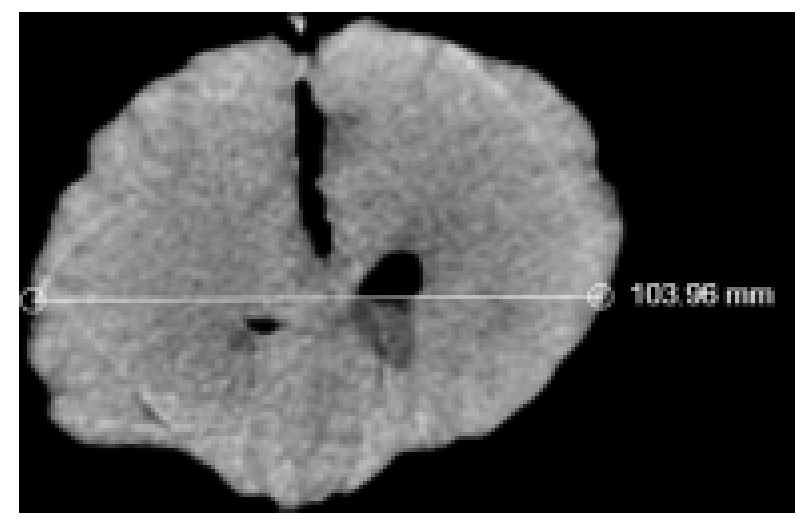</td>
</tr>
<tr>
<td>3/06/25:102.21 mm at 84
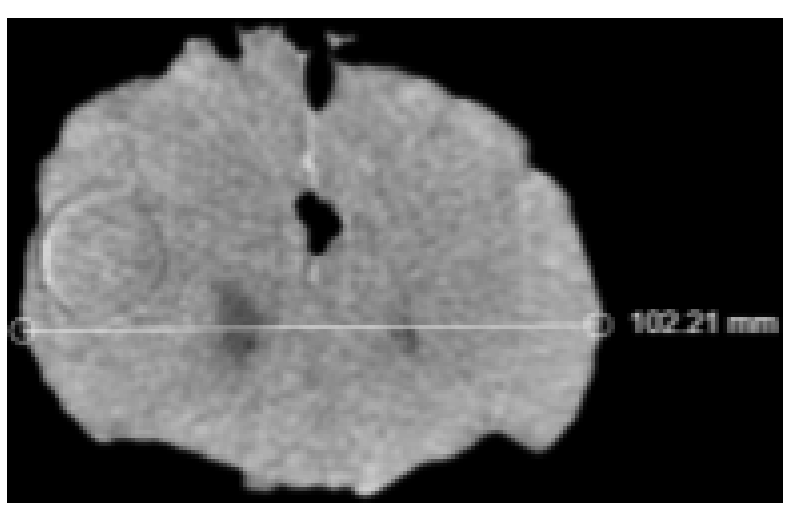</td>
<td>3/06/25:105.39 mm at 79
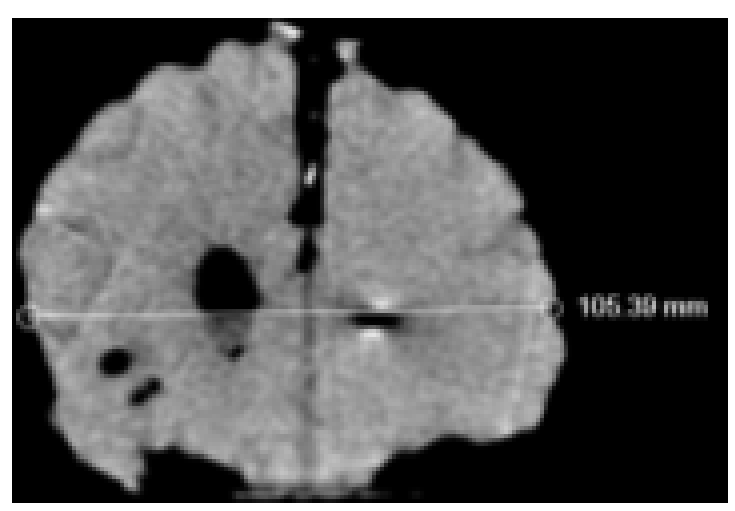</td>
<td>3/06/25:102.56 mm at 90
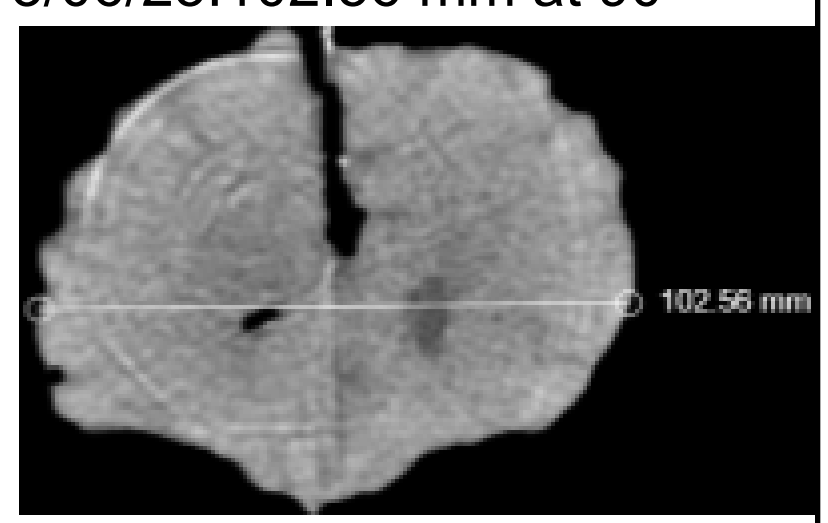</td>
</tr>
<tr>
<td>3/13/25:100.44 mm at 173
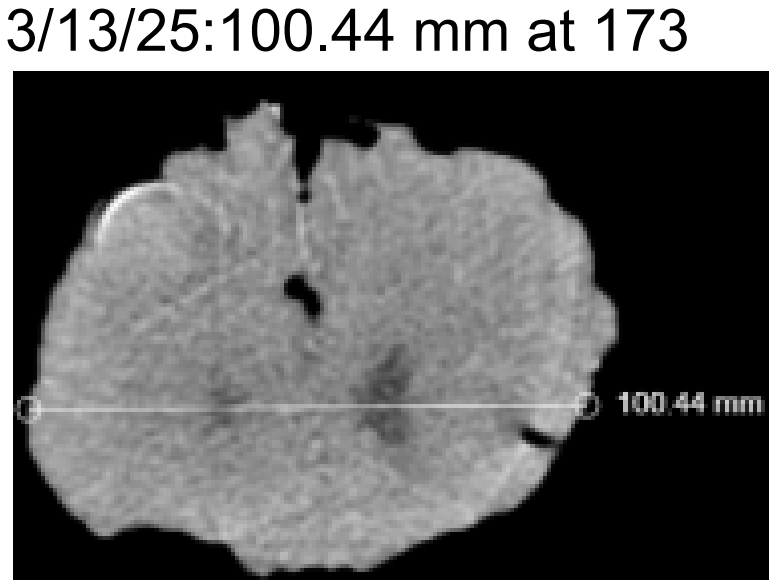</td>
<td>3/13/25:106.78 mm at 158
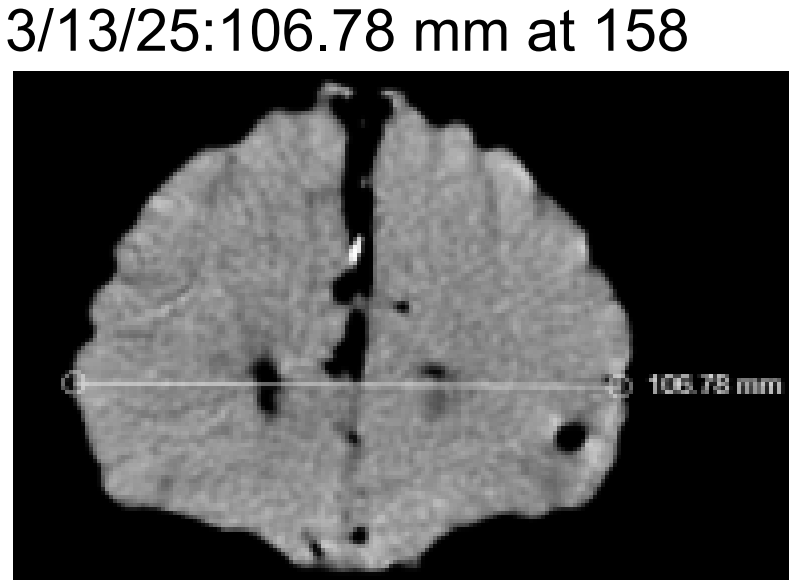</td>
<td>3/13/25:102.21 mm at 174
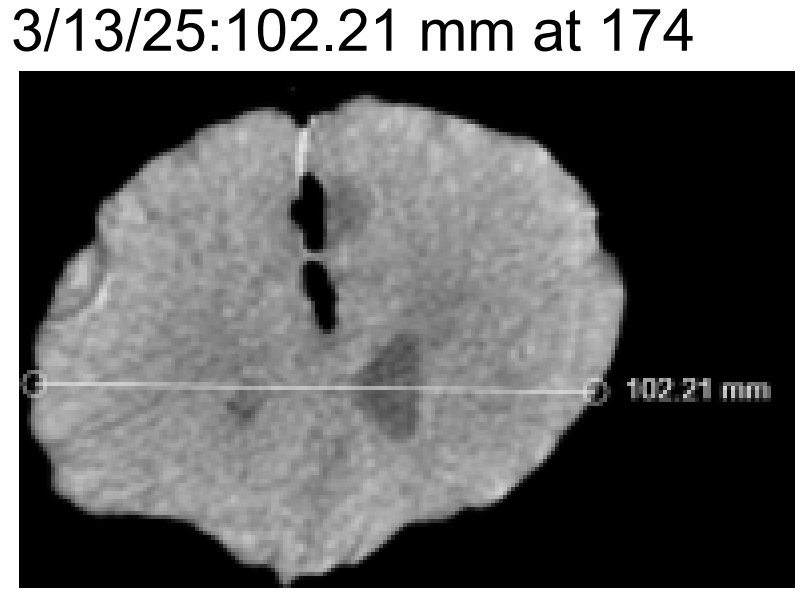</td>
</tr>
</table>

4/17/25:101.50 mm at 181

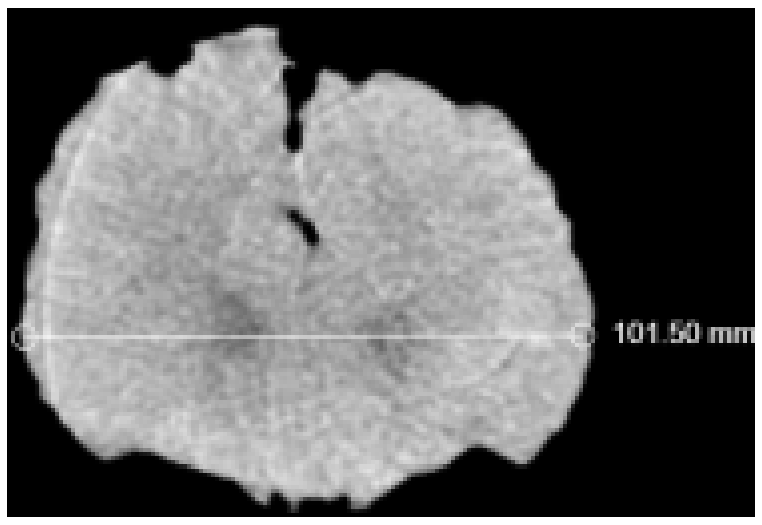

4/17/5:105.37 mm at 155

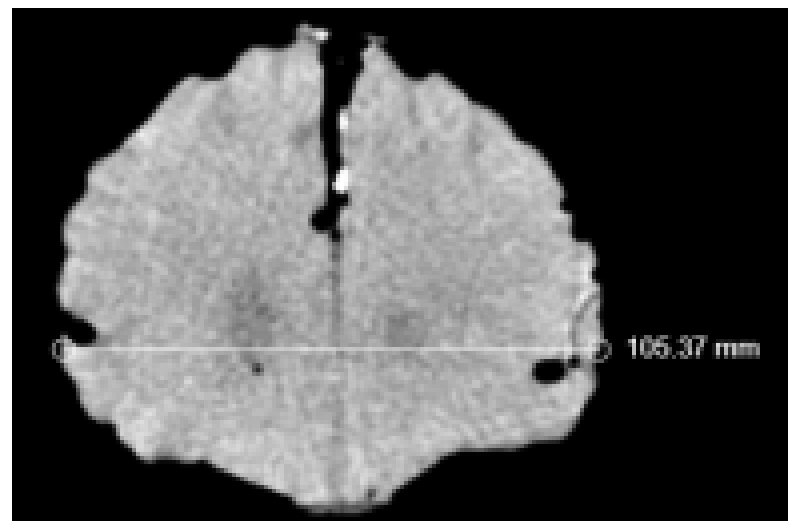

4/17/25:101.85 mm at 168

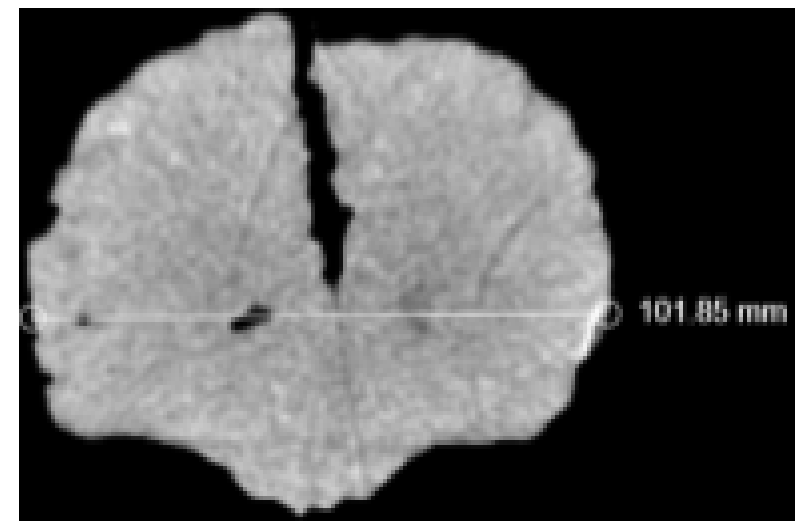

5/2/25:101.85 mm at 120

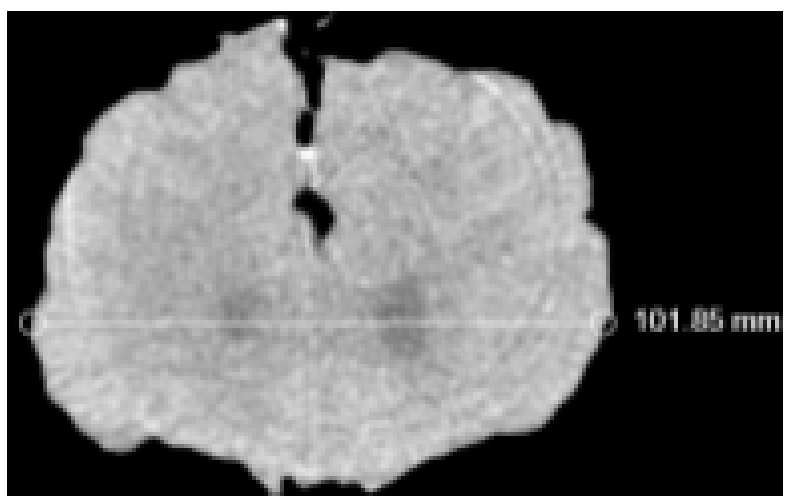

5/2/25:106.08 mm at 133

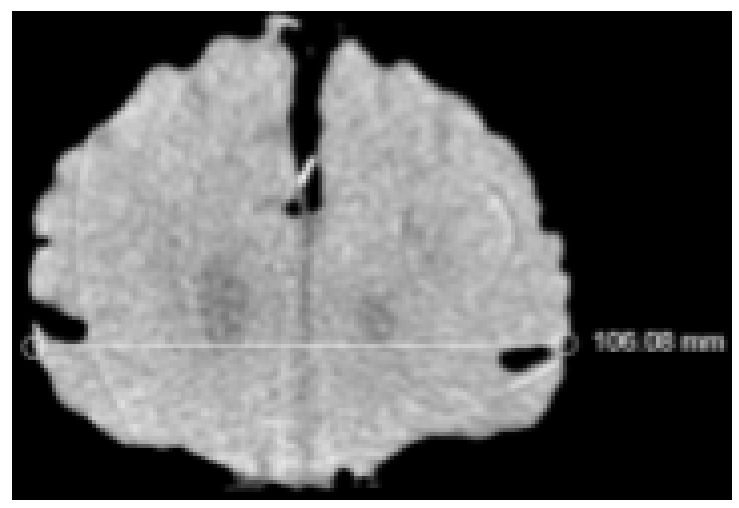

5/2/25:100.45 mm at 145

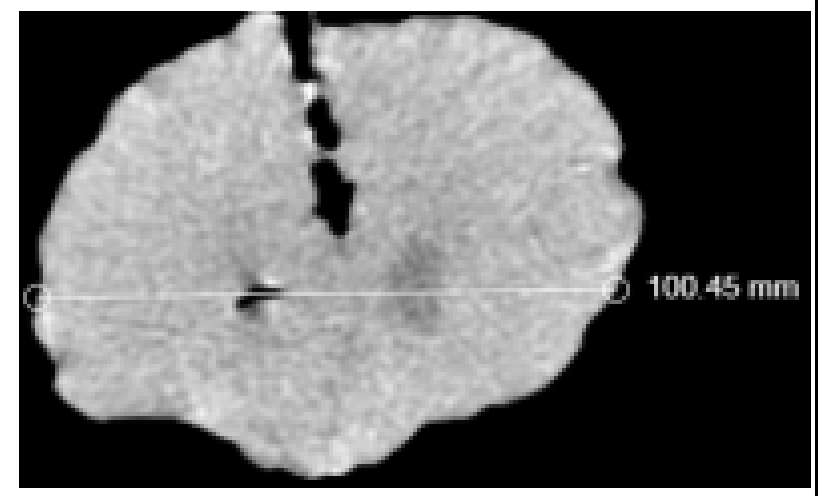

5/7/25:101.50 mm at 158

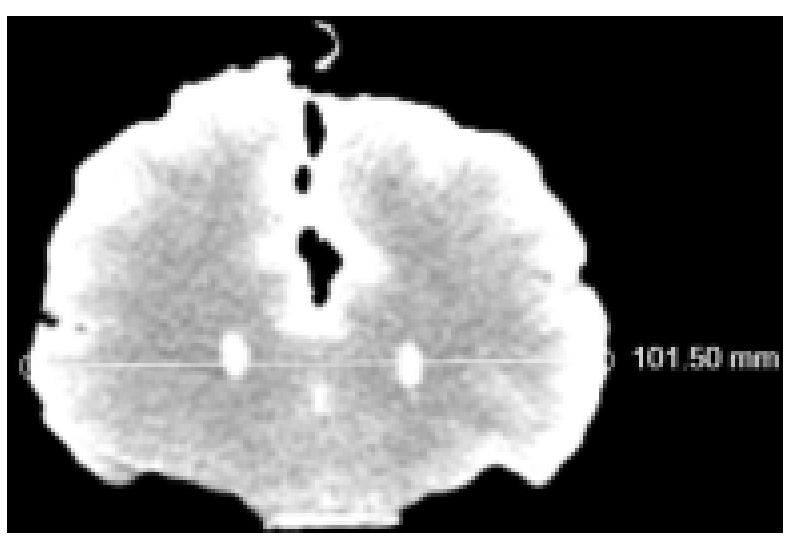

5/7/25:106.43 mm at 171

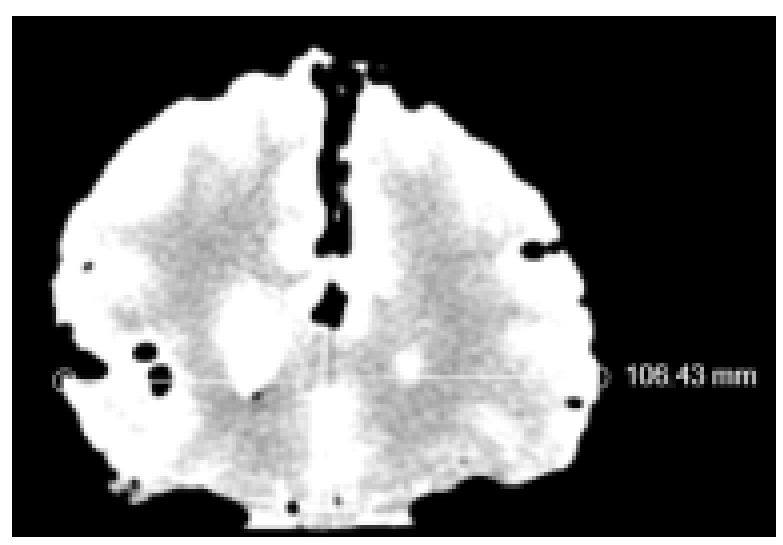

5/7/25:99.73 mm at 133

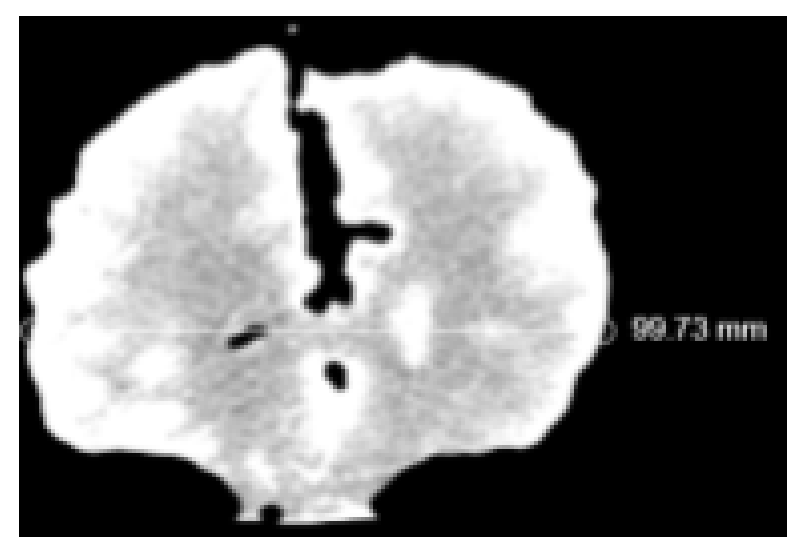

5/15/25:99.03 mm at 176

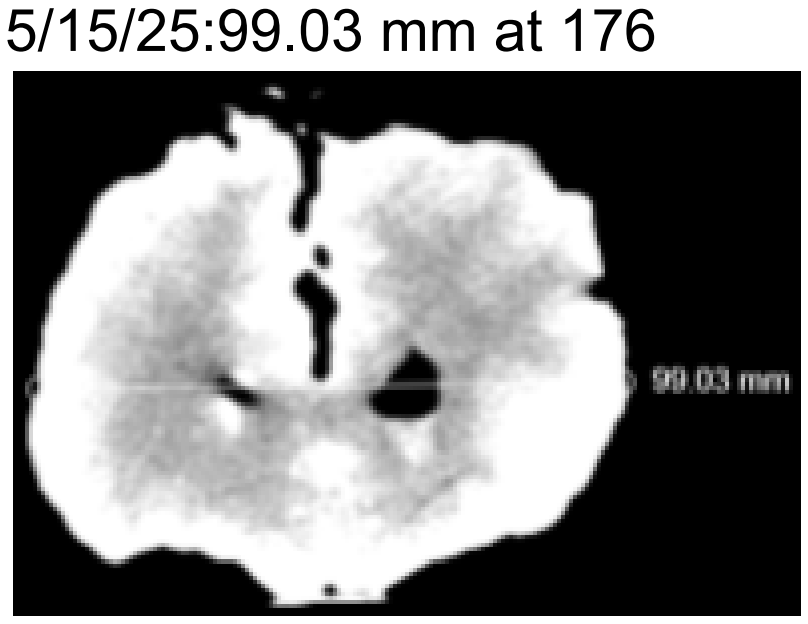

5/15/25:106.43 mm at 148

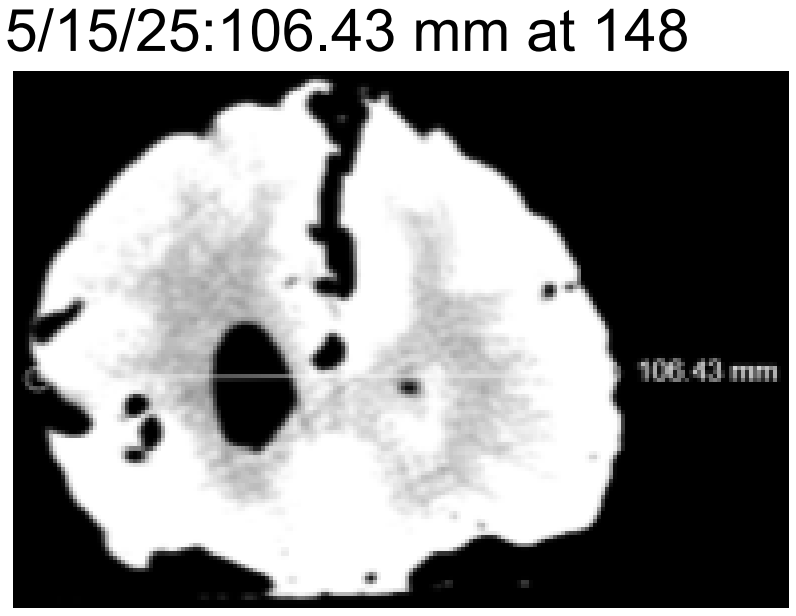

5/15/2025:108.19 mm at 141

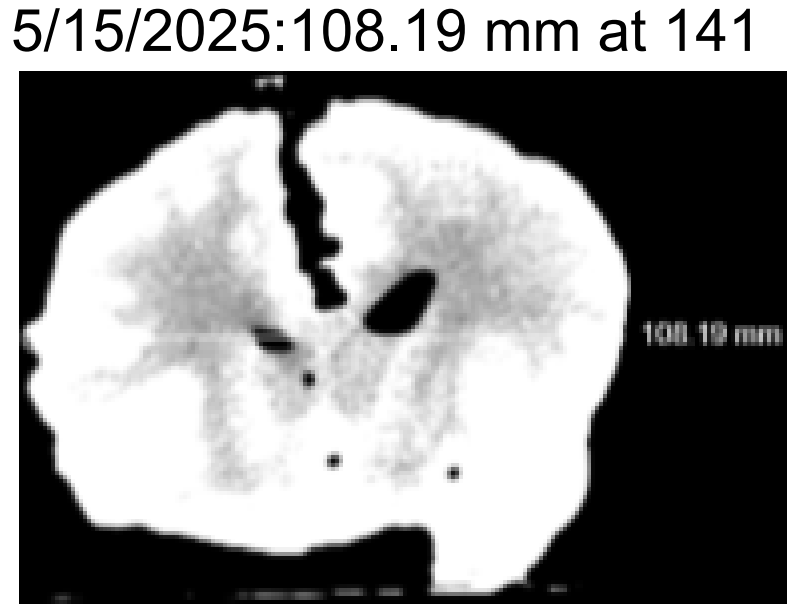

08/19/25:101.14 mm at 183 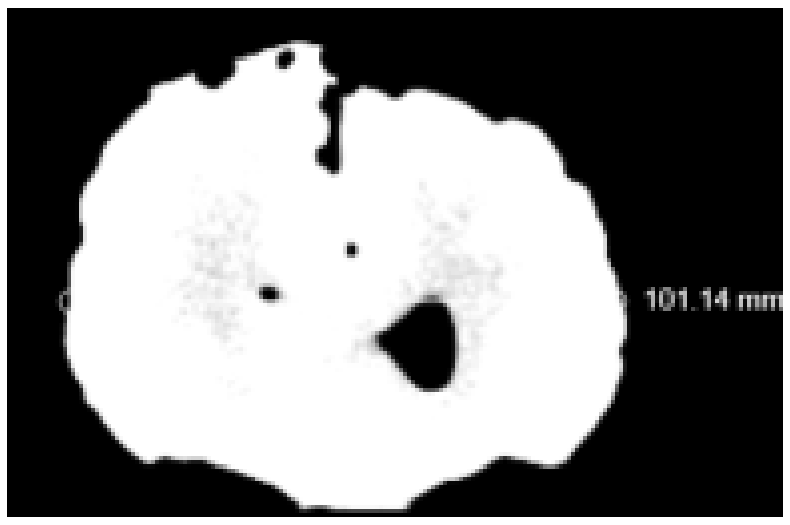	08/19/25:106.79 mm at 129 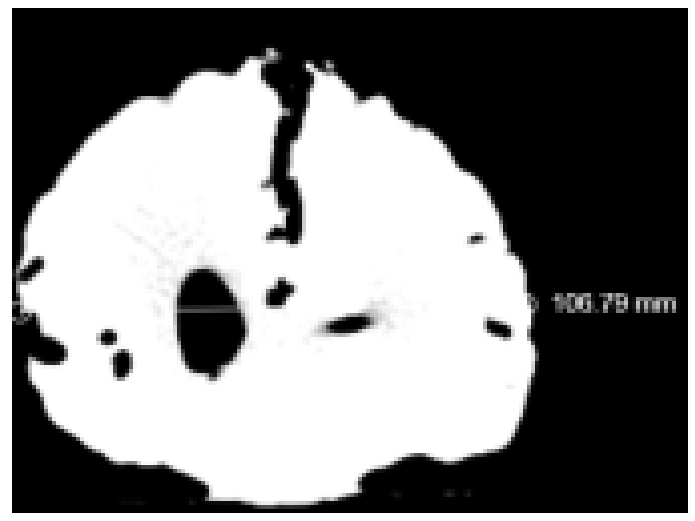	08/19/25:107.85 mm at 181 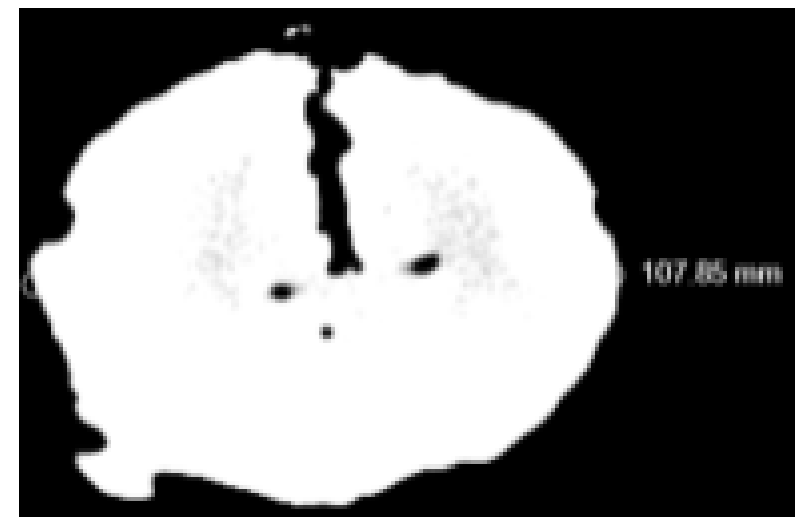
11/04/25:100.79 at 161 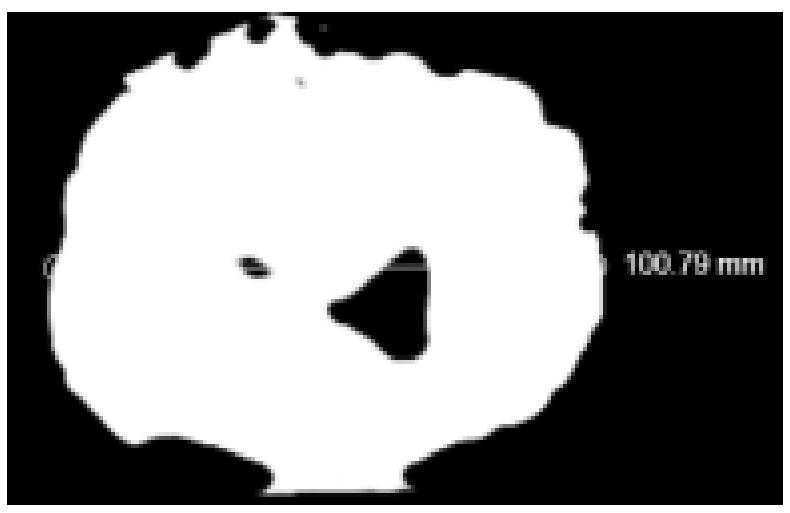	11/04/25:106.43 mm at 166 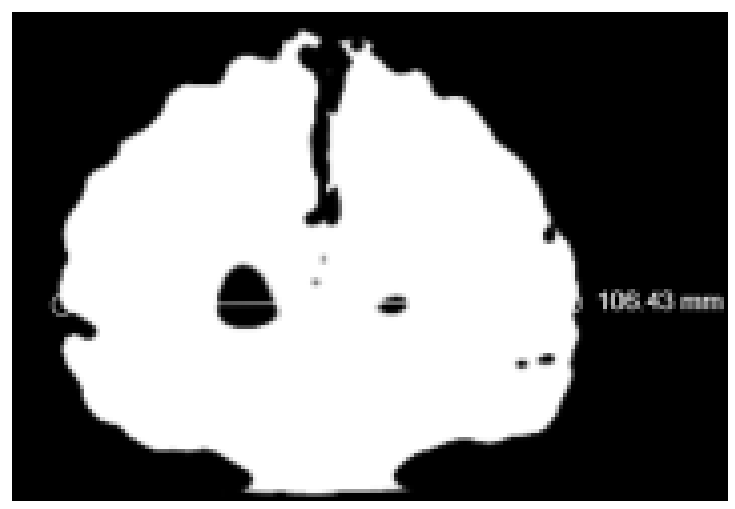	11/04/25:114.18 mm at 113 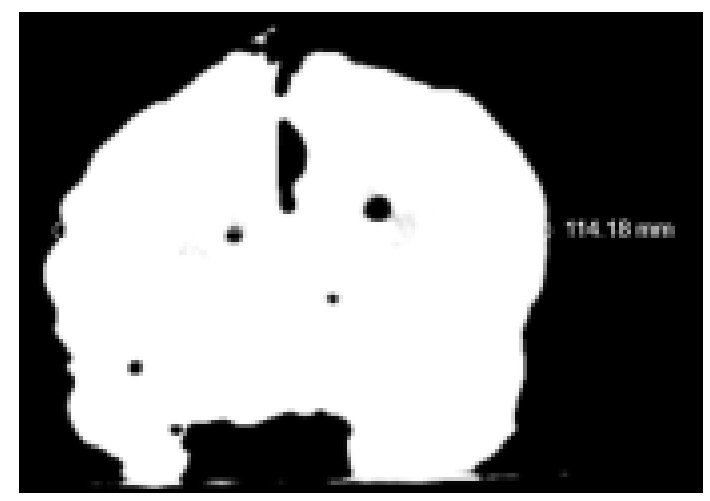
11/21/25:100.44 at 123 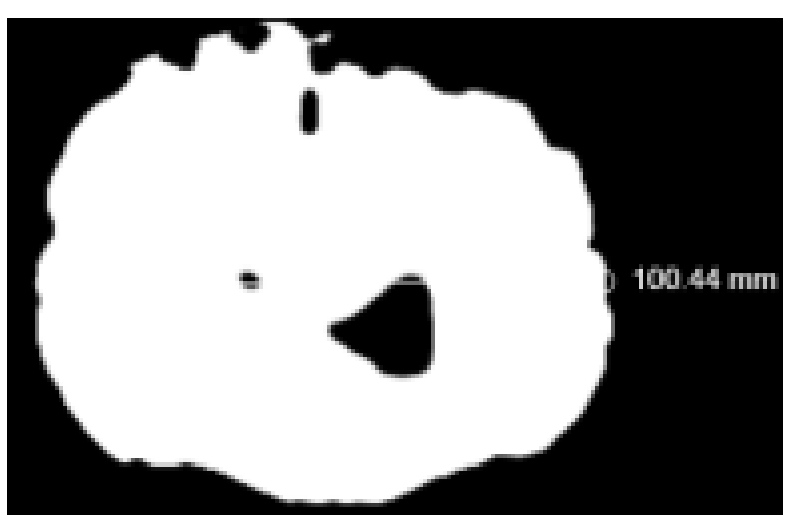	11/21/25:105.38 mm at 103 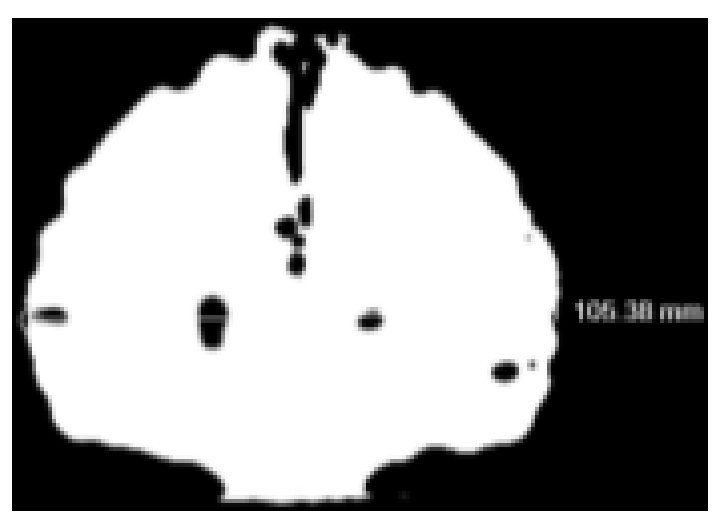	11/21/25:117.71 mm at 177 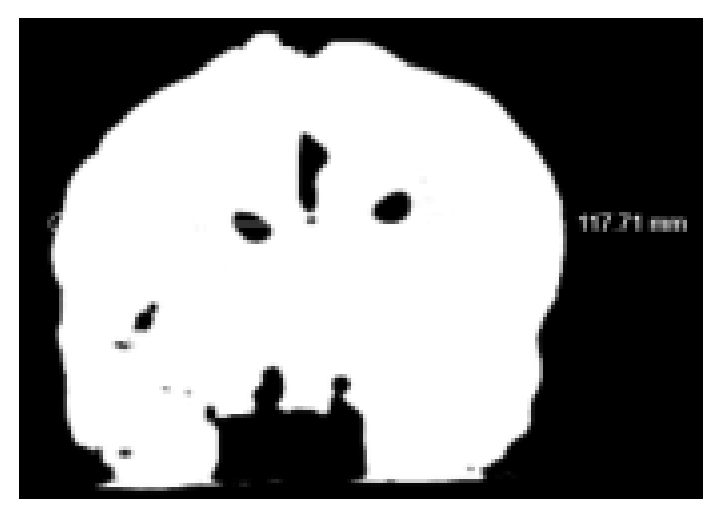
12/04/25 Placed in Freezer	12/04/25 Placed in Freezer	12/04/25 Placed in Freezer
