# Supplementary material for: Cryopreservation of aldehyde-fixed whole brains
Source: PLoS One. 2026 Aug 24;21(8):e0344932. doi: 10.1371/journal.pone.0344932 (PMC13502592; doi:10.1371/journal.pone.0344932)
Supplement: Supplementary file 2 [file pone.0344932.s002.pdf]

CT scans

Format: Date:measurement mm @ frame number

DN 54	DN 137	DN 180
2/18/25:162.90 mm at 395<br>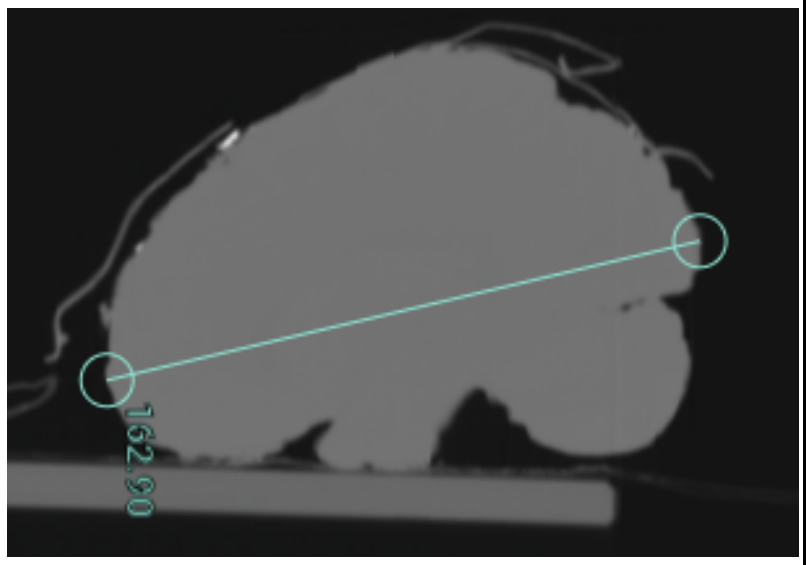	2/18/25:163.74 mm at 272<br>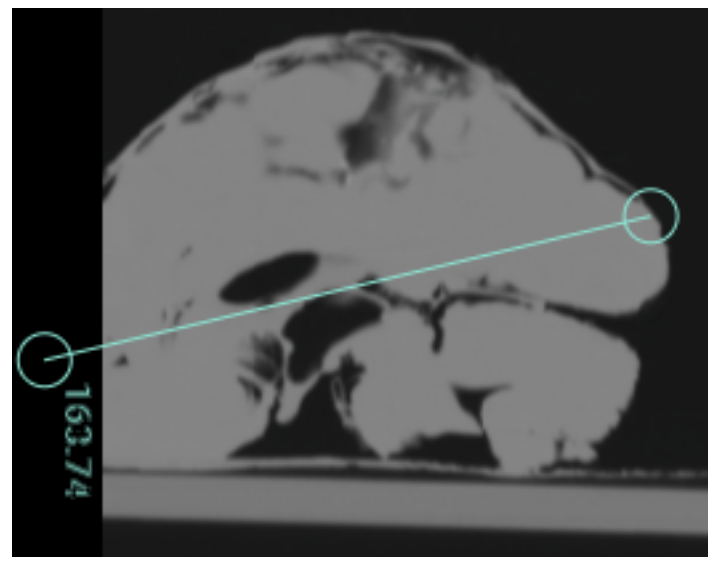	2/18/25:160.85 mm at 288<br>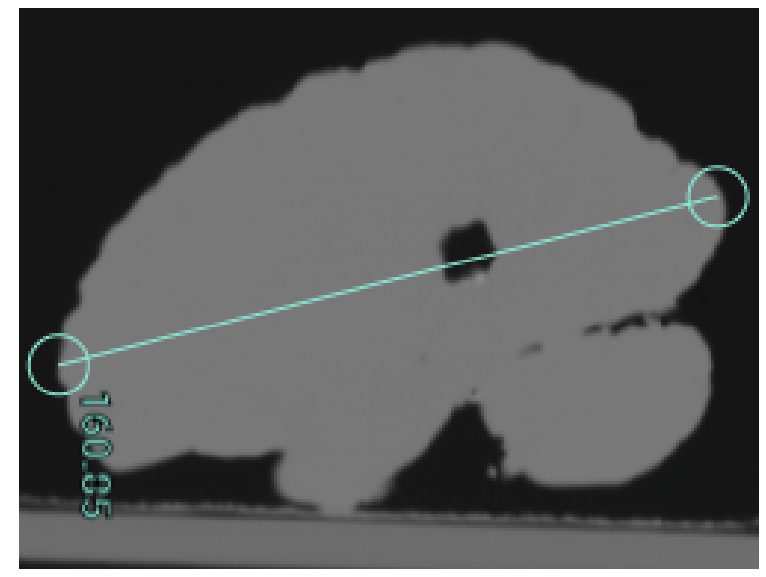
2/27/25:161.31 mm at 257<br>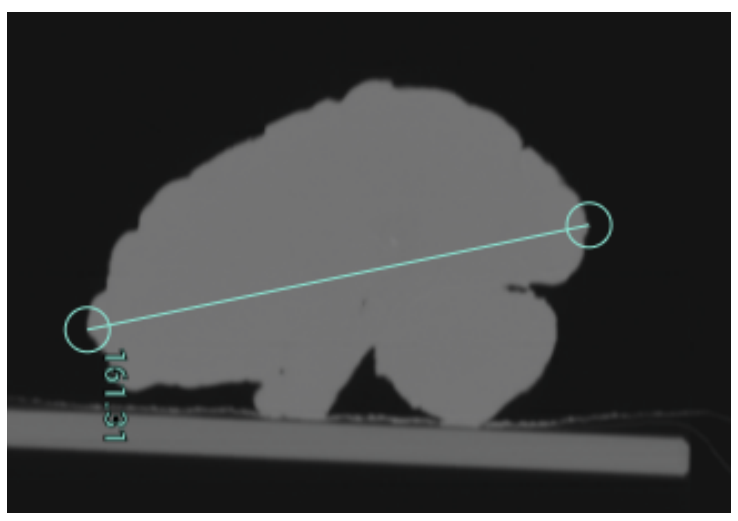	2/27/25:148.14 mm at 262<br>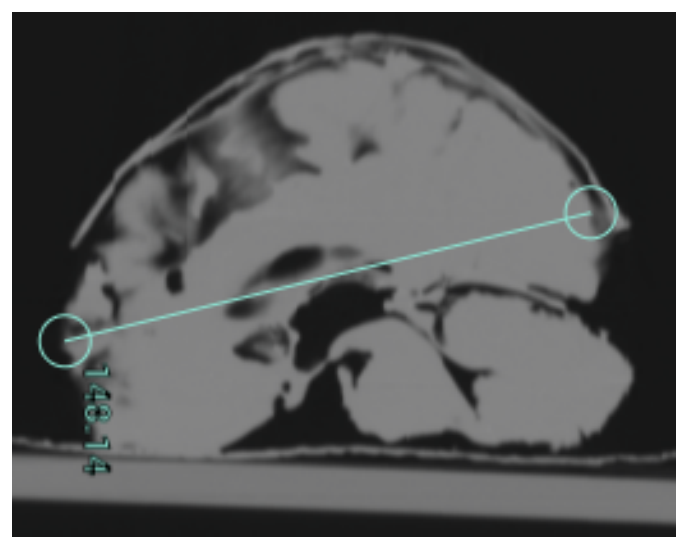	2/27/25:167.07 at 261<br>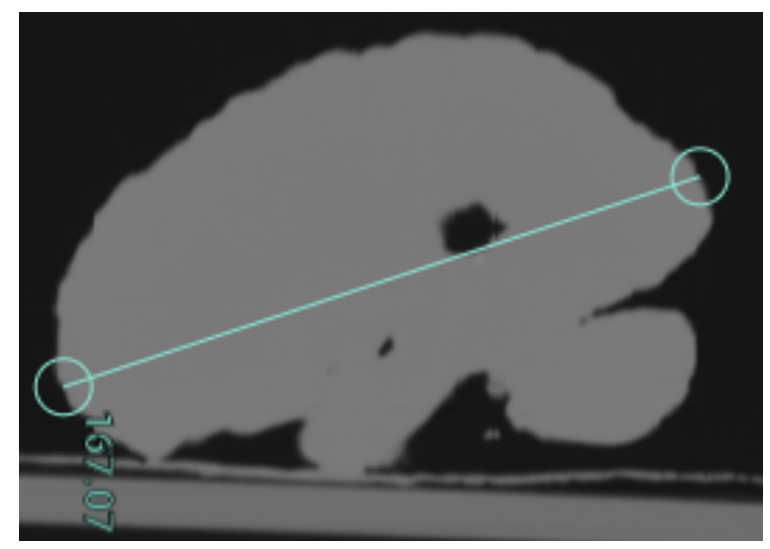
3/06/25:156.65 mm at 282<br>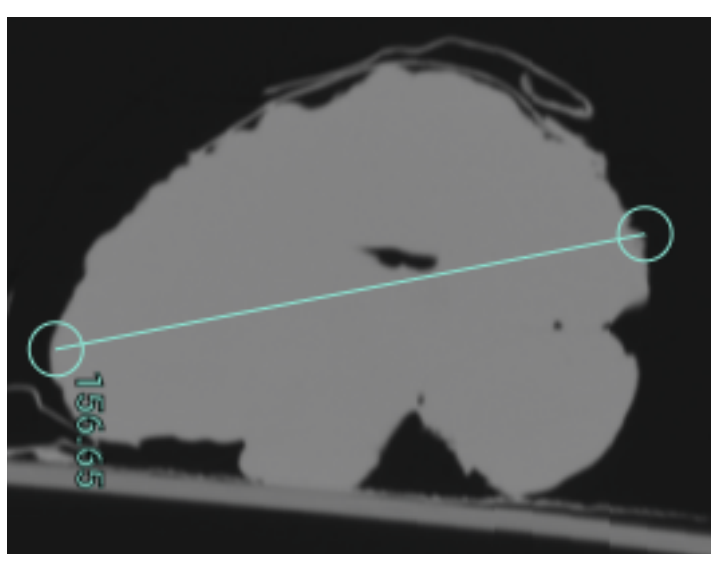	3/06/25:151.73 mm at 283<br>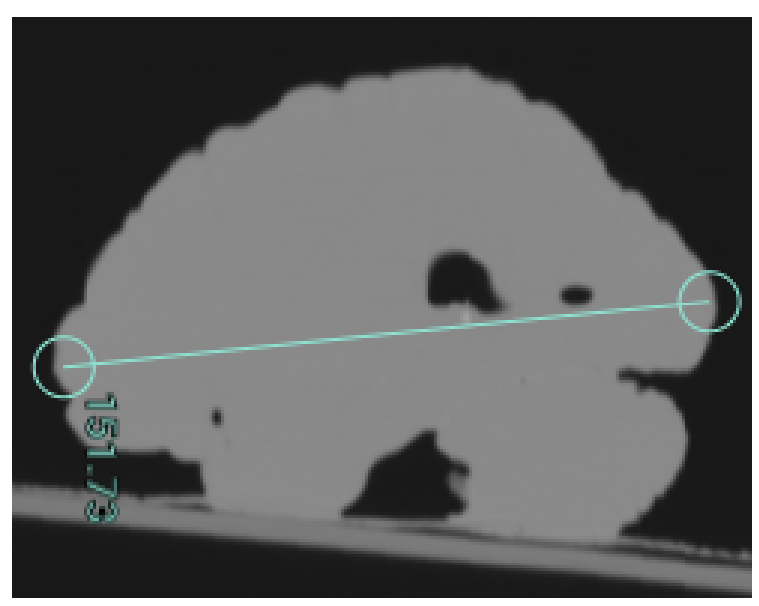	3/06/25:163.23 mm at 239<br>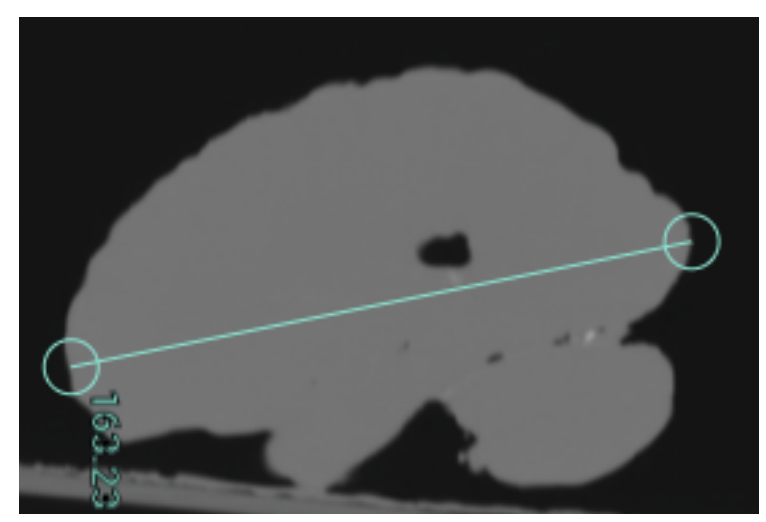
3/13/25:152.12 mm at 286<br>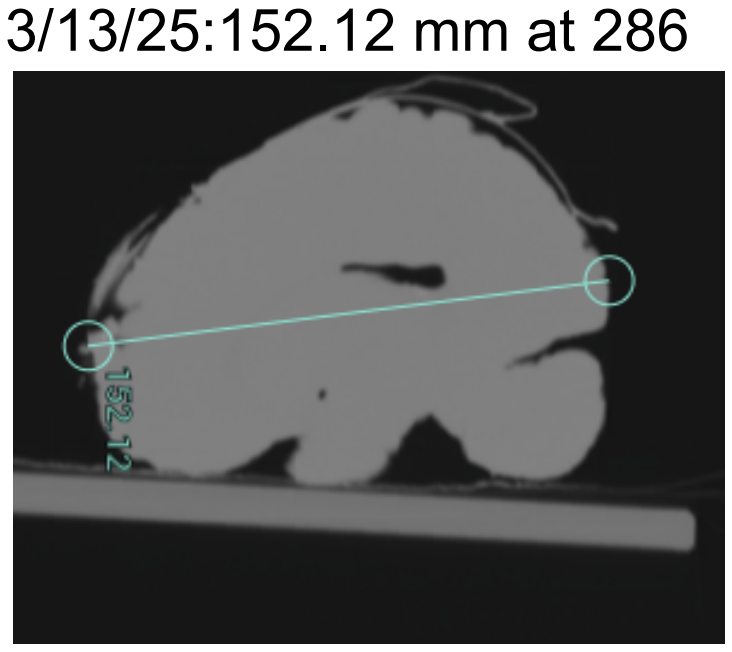	3/13/25:156.13 mm at 292<br>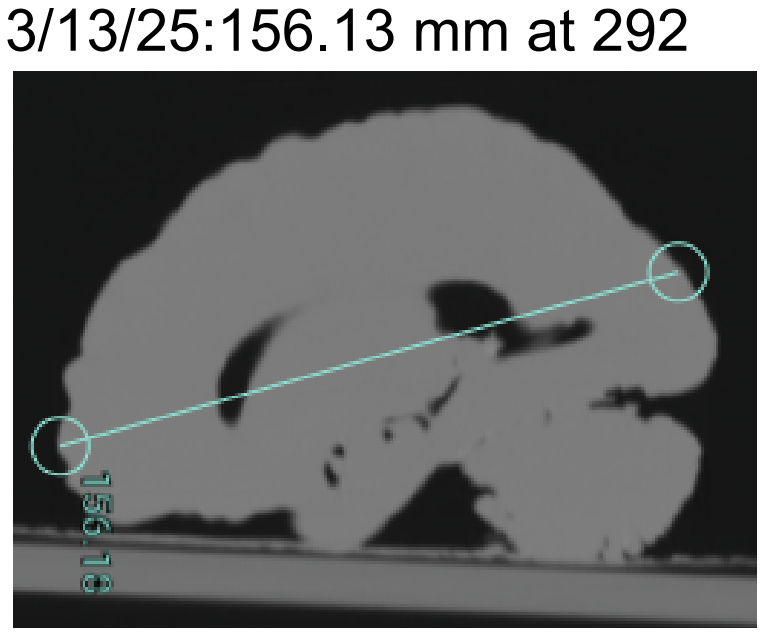	3/13/25:167.41 mm at 309<br>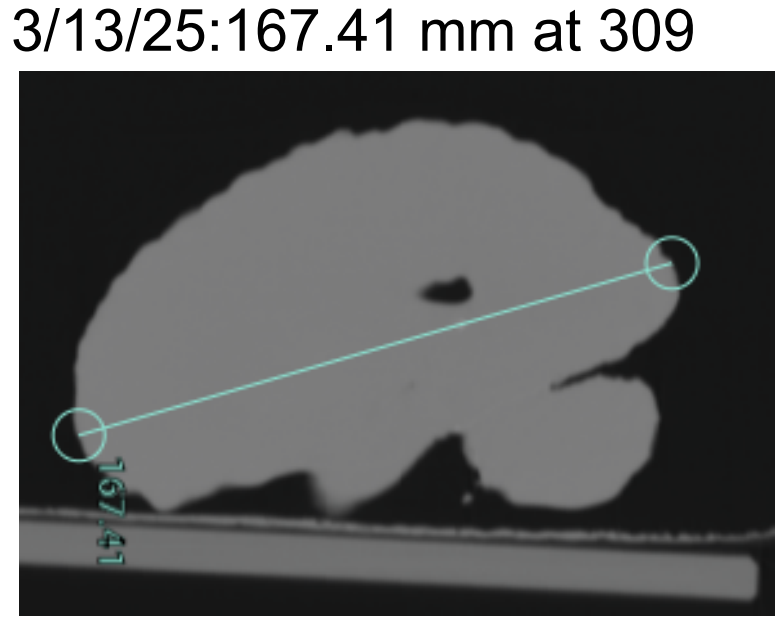

4/17/25:160.00 mm at 230<br>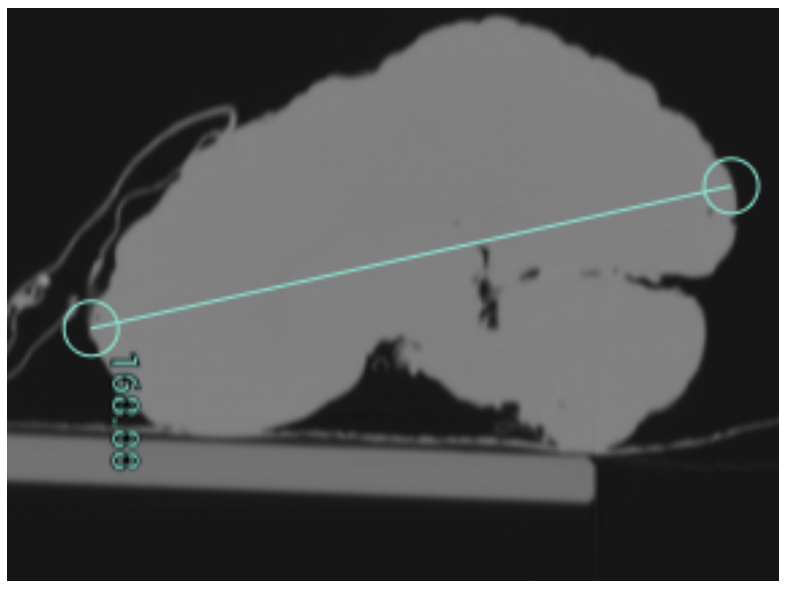	4/17/5:161.22 mm at 195<br>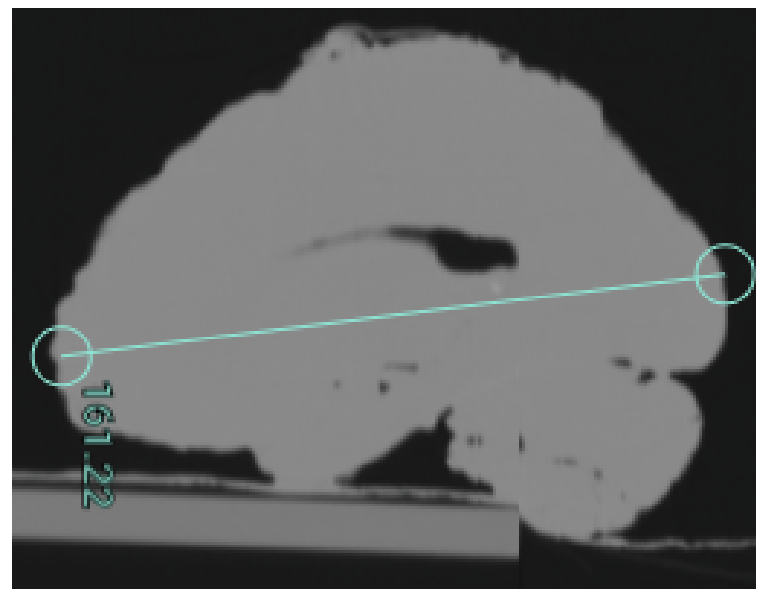	4/17/25:153.05 mm at 207<br>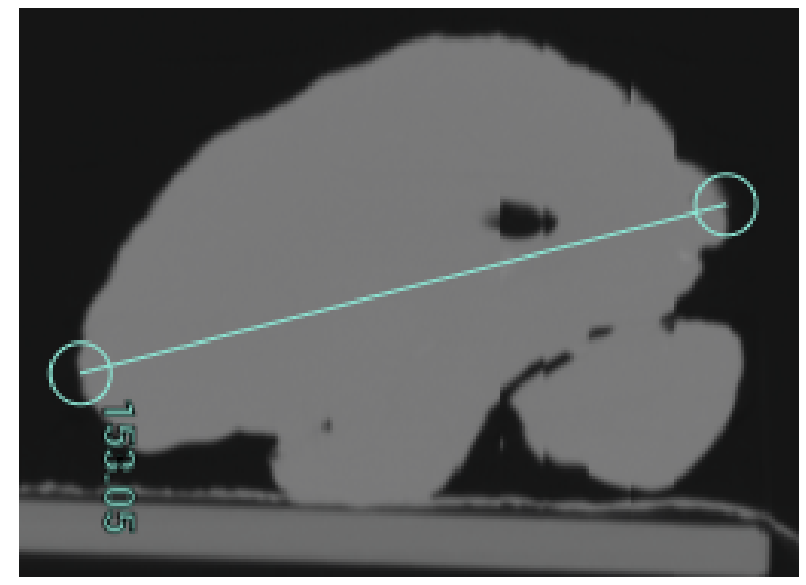
5/2/25:155.14 mm at 285<br>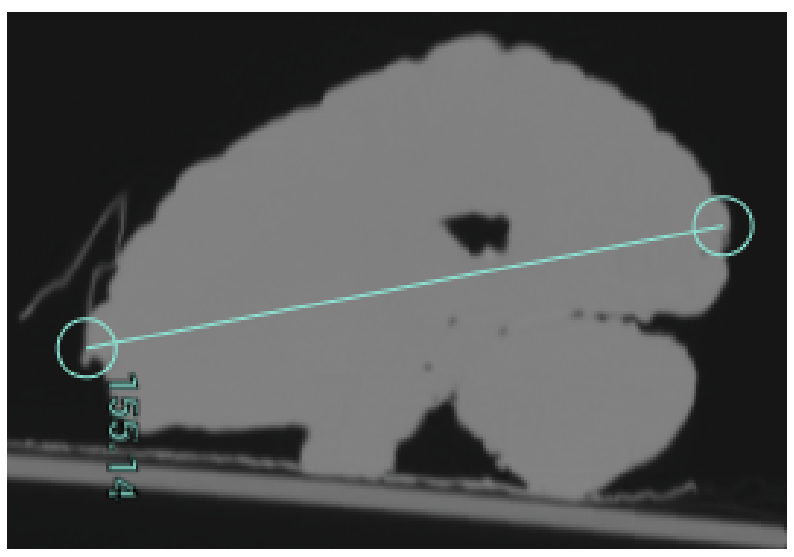	5/2/25:158.34 mm at 218<br>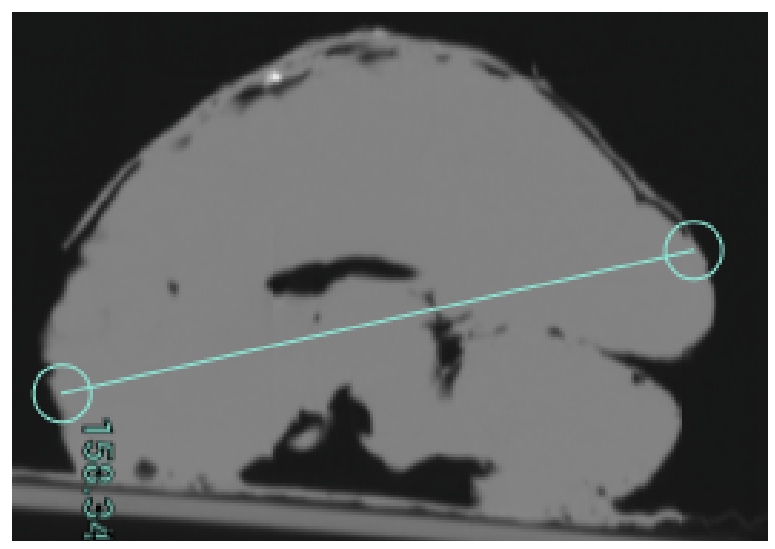	5/2/25:158.53 mm at 223<br>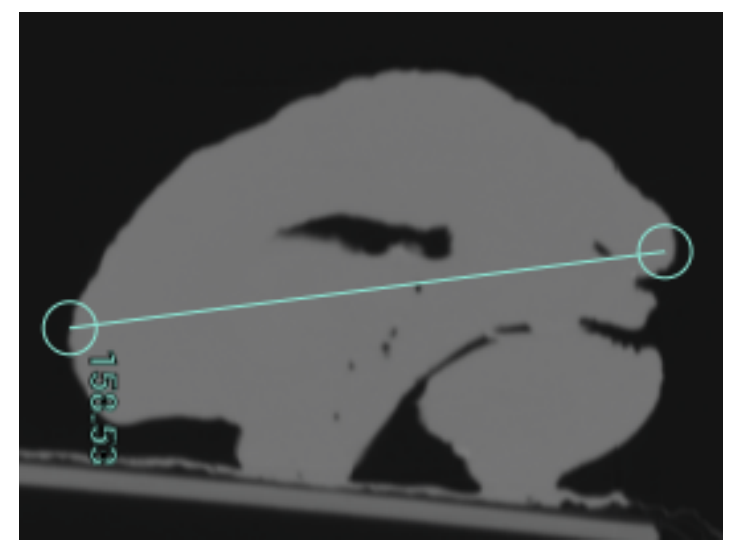
5/7/25:152.09 mm at 297<br>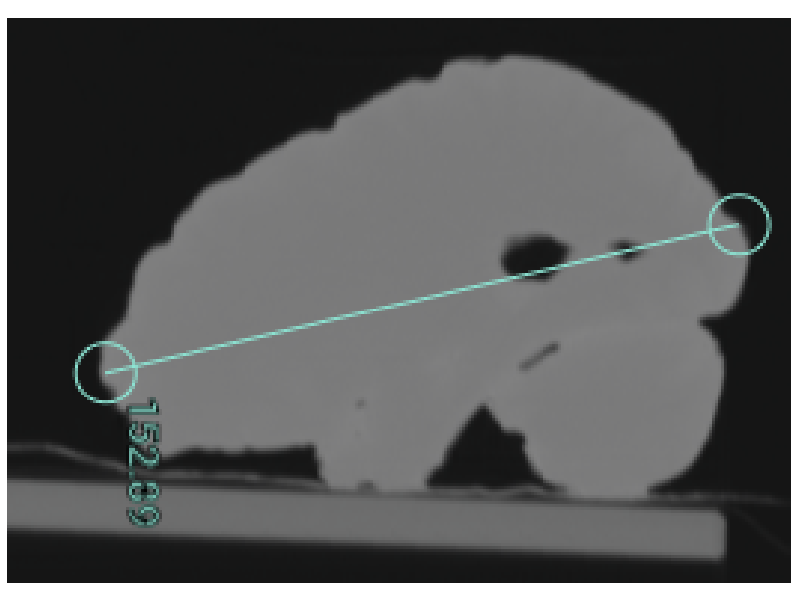	5/7/25:166.01 mm at 269<br>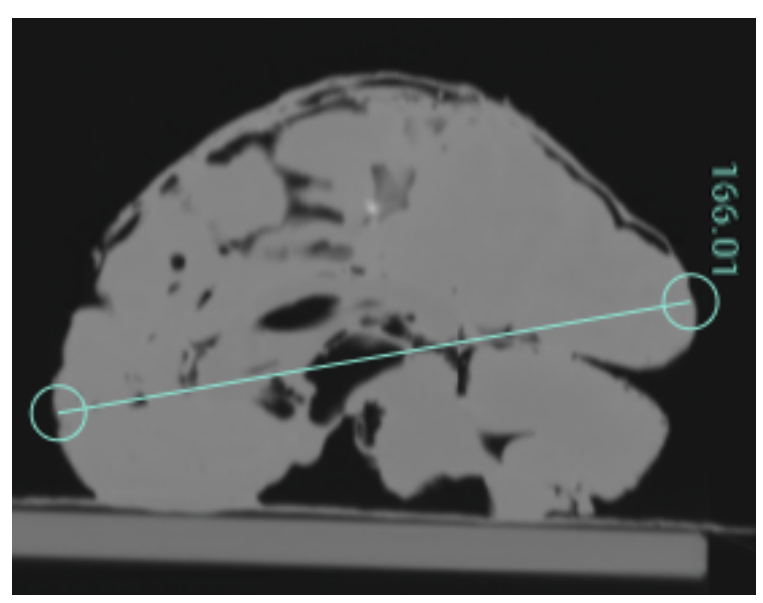	5/7/25: 158.46 mm at 283<br>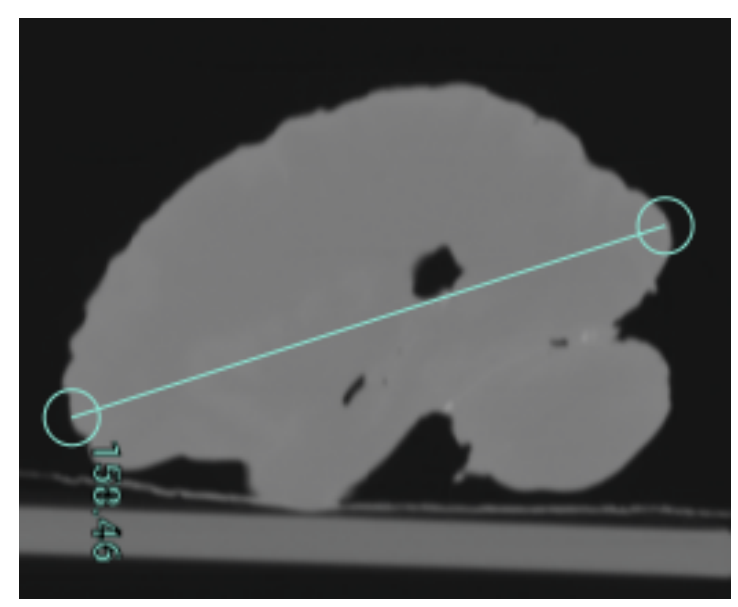
5/15/25:152.62 mm at 246<br>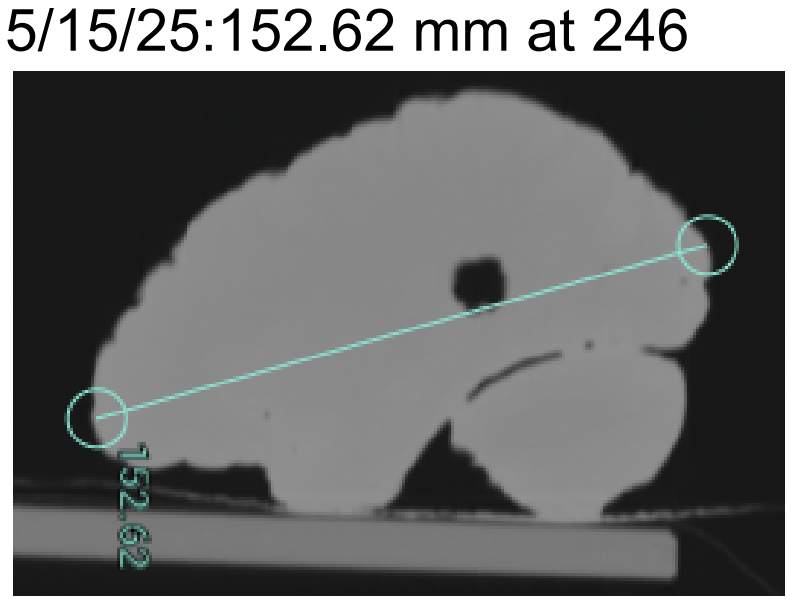	5/15/25:159.82 mm at 226<br>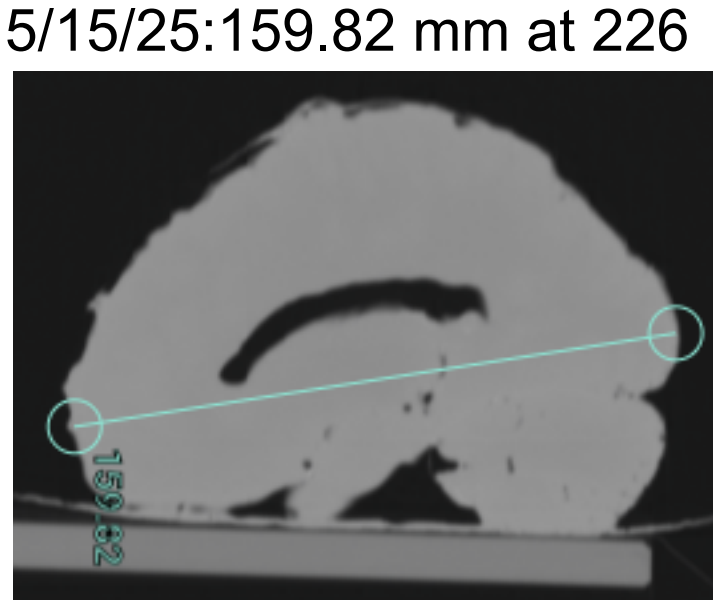	5/15/2025:157.62 mm at 249<br>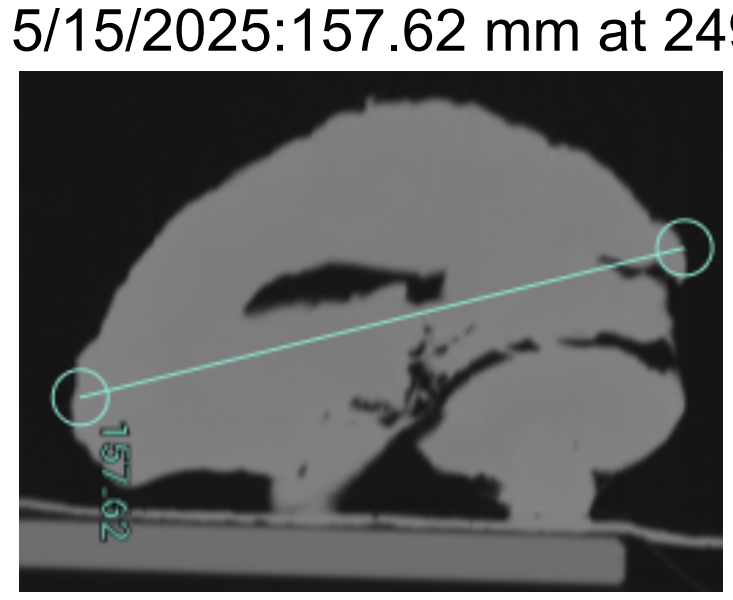

08/19/25: 159.35 mm at 271<br>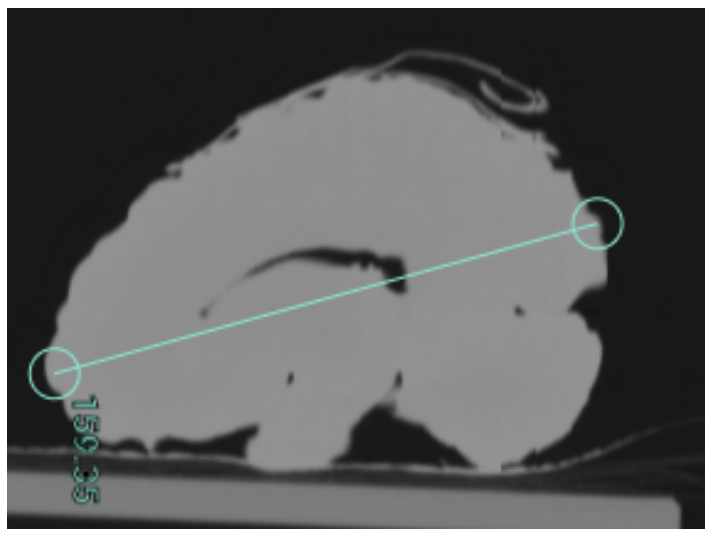	08/19/25:165.02 mm at 294<br>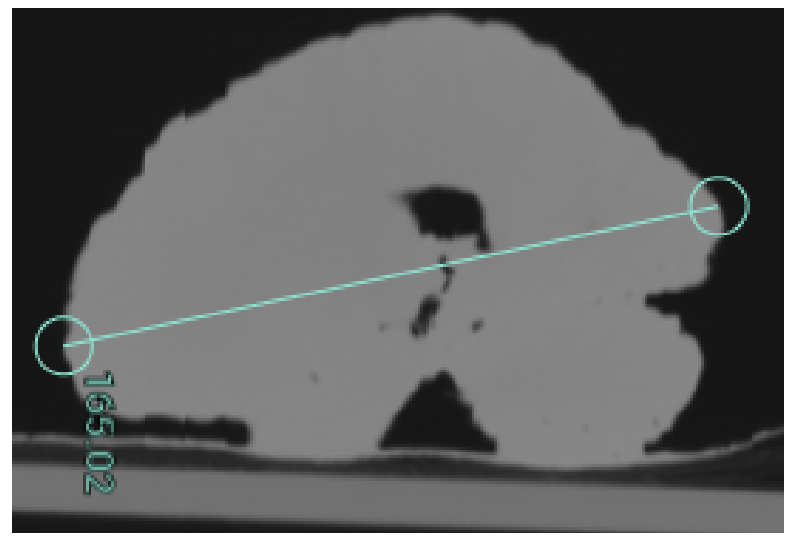	08/19/25:154.75 mm at 280<br>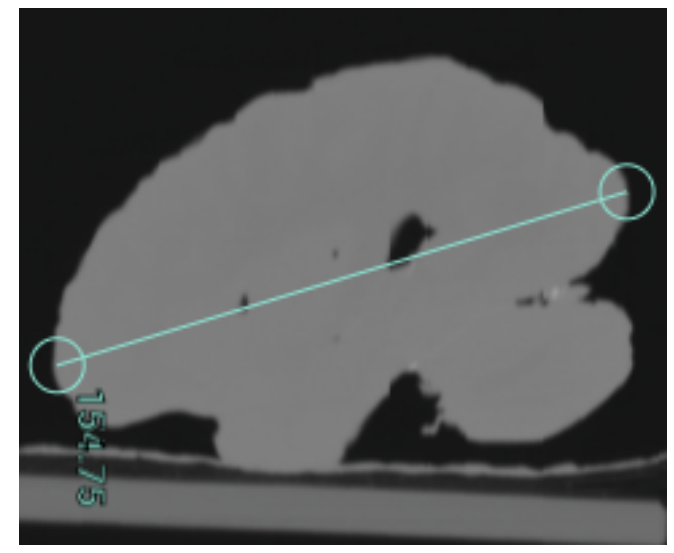
11/04/25:155.01 at 239<br>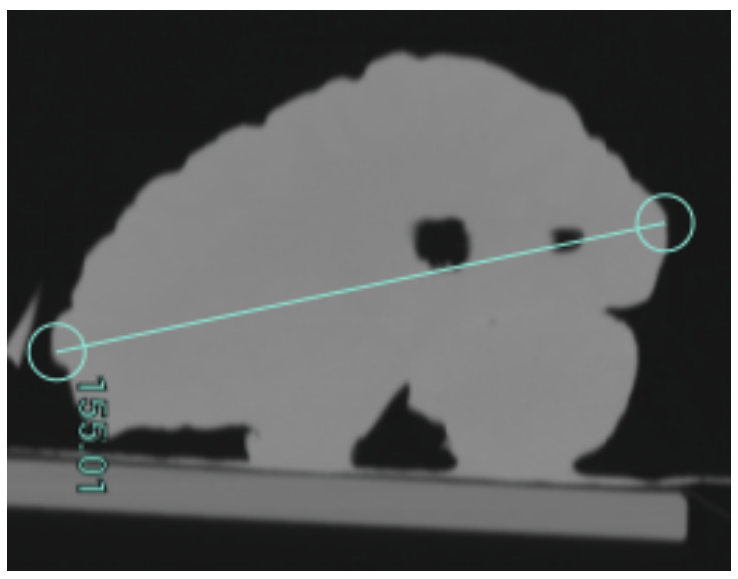	11/04/25:165.31 mm at 248<br>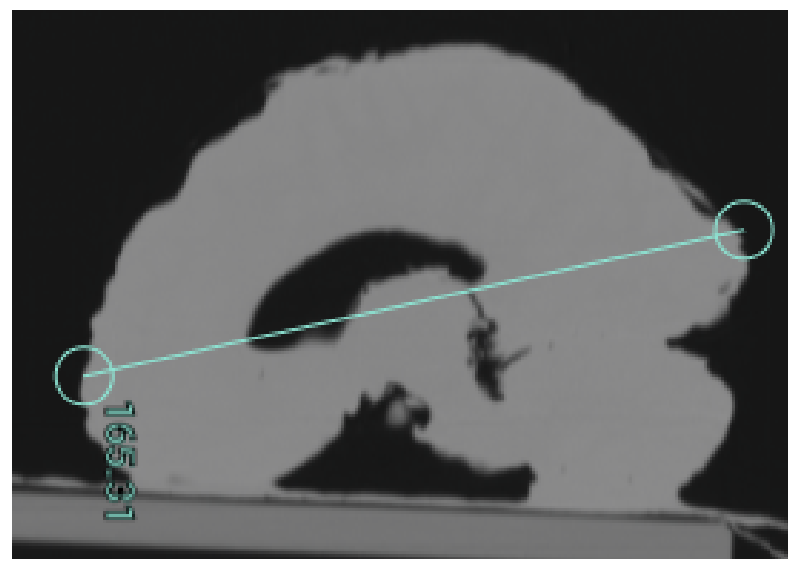	11/04/25:161.10 mm at 279<br>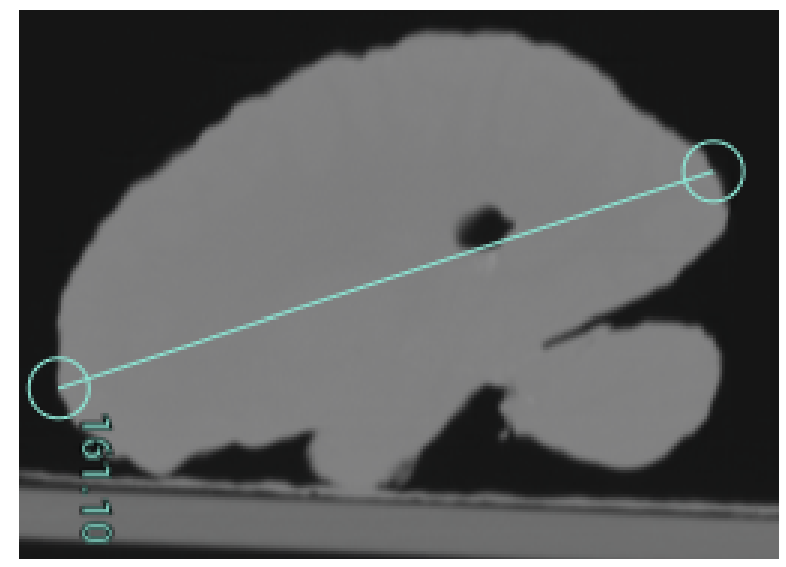
11/21/25:157.35 at 261<br>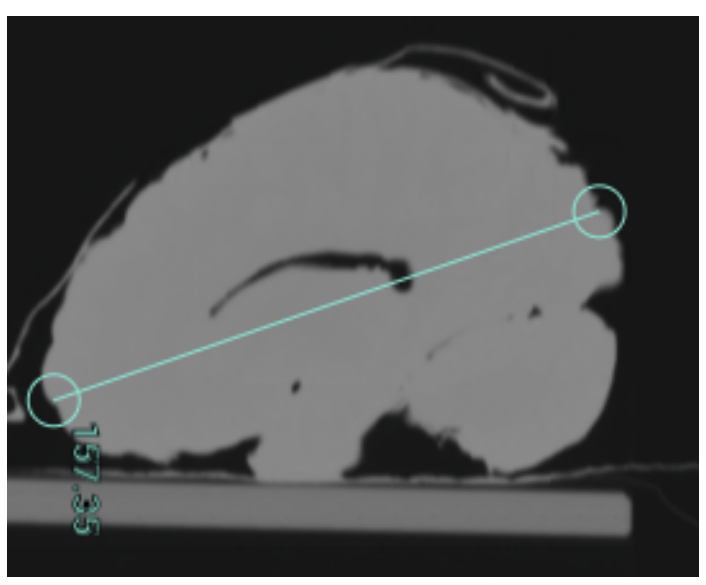	11/21/25:105.38 mm at 103<br>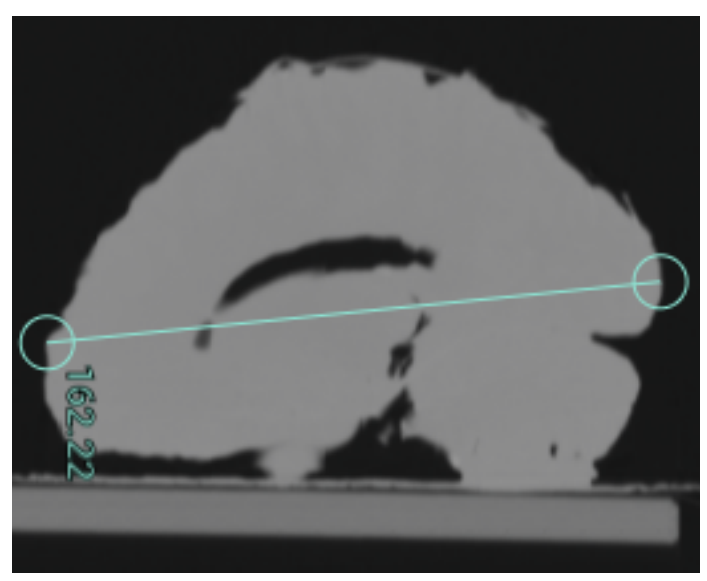	11/21/25:162.01 mm at 209<br>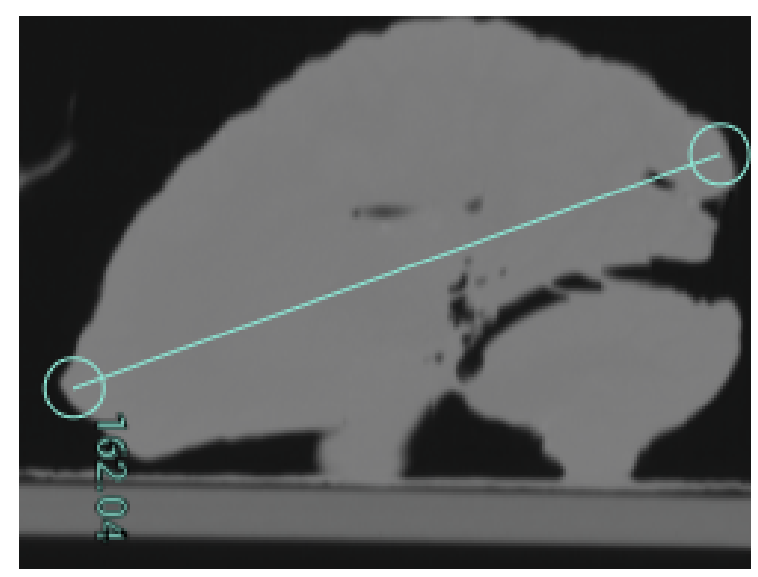
12/04/25 Placed in Freezer	12/04/25 Placed in Freezer	12/04/25 Placed in Freezer
