# Supplementary material for: Cryopreservation of aldehyde-fixed whole brains
Source: PLoS One. 2026 Aug 24;21(8):e0344932. doi: 10.1371/journal.pone.0344932 (PMC13502592; doi:10.1371/journal.pone.0344932)
Supplement: Supplementary file 3 [file pone.0344932.s004.pdf]

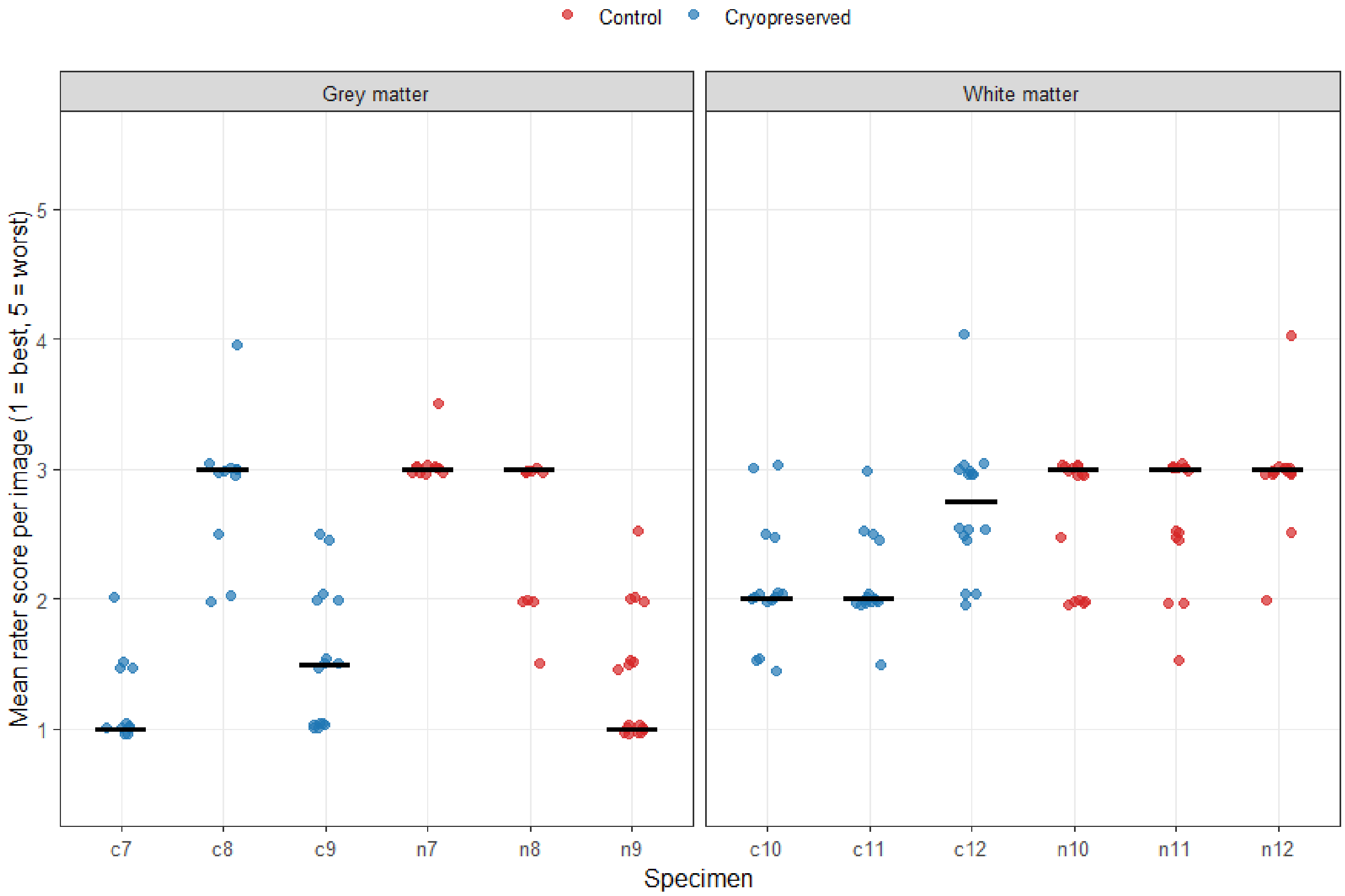

Control
Cryopreserved
Grey matter
White matter
Mean rater score per image (1 = best, 5 = worst)
5
4
3
2
1
c7
c8
c9
n7
n8
n9
c10
c11
c12
n10
n11
n12
Specimen
