# Supplementary material for: Cryopreservation of aldehyde-fixed whole brains
Source: PLoS One. 2026 Aug 24;21(8):e0344932. doi: 10.1371/journal.pone.0344932 (PMC13502592; doi:10.1371/journal.pone.0344932)
Supplement: Supplementary file 4 [file pone.0344932.s008.pdf]

Response to Editor and Reviewer Comments

We thank the Editor and Reviewers for their helpful comments. Below, we provide detailed responses.

Response to Editor

Editor Comment: *Dear Authors, thank you for submitting your manuscript. While the topic is important and potentially valuable, the current version has several significant limitations that affect its scientific rigor and clarity; therefore, a decision of Major Revision has been made. The study lacks a clear hypothesis-driven design, and the small sample size (n = 3) combined with the absence of adequate statistical analysis weakens the reliability of the conclusions. Additionally, the introduction does not clearly define the knowledge gap or novelty, and there is inconsistency between the stated aims and the methods and results presented. Many findings are described qualitatively without sufficient quantification, and the overall structure—particularly the distinction between Results and Discussion—requires reorganization. Methodological details are also insufficient to ensure reproducibility, and the quality of figures and tables needs improvement. Addressing these issues is essential before the manuscript can be reconsidered for publication.*

We thank the Editor for their kind comments and synthesis of the key concerns. We have addressed these issues through the following major revisions:

1. We have substantially revised the Introduction to more clearly articulate the two specific aims of this paper: (a) characterizing cryoprotectant diffusion kinetics in whole human brains, and (b) assessing whether the cryoprotectant loading and unloading process introduces structural artifacts. This better fits with the methods and results actually presented.
2. We have expanded the Methods section to improve reproducibility, including additional detail on solution preparation, bath-to-tissue volume ratios, and protocol modifications. While doing so, we noticed that the original 10 month figure for cryoprotectant loading inadvertently included the preceding fixation period. The loading protocol alone lasts approximately 9 months, and the final equilibration step approximately 7 months. We have corrected both throughout the manuscript.
3. We have added more discussion of statistical power limitations and additional effect size reporting. We have also tempered our claims regarding ultrastructural equivalence because of the inferential limits based on the sample size.
4. We have reorganized the Discussion by adding in points and limitations brought up by the reviewers.
5. We have improved the quality of the figures.

Response to Journal Requirements

Requirement 1: *Please ensure that your manuscript meets PLOS ONE's style requirements, including those for file naming.*

We have attempted to ensure that our manuscript is formatted according to the PLOS ONE style templates.

Requirement 2: *Please note that funding information should not appear in any section or other areas of your manuscript. We will only publish funding information present in the Funding Statement section of the online submission form. Please remove any funding-related text from the manuscript.*

We have removed the Funding section from the manuscript. All funding information is now provided only in the cover letter.

Requirement 3: *We note that the grant information you provided in the 'Funding Information' and 'Financial Disclosure' sections do not match.*

When you resubmit, please ensure that you provide the correct grant numbers for the awards you received for your study in the 'Funding Information' section.

We have reviewed the grant information to ensure the correct grant numbers are provided. This is provided in the cover letter.

Requirement 4: *Please expand the acronym "NIH" (as indicated in your financial disclosure) so that it states the name of your funders in full.*

This information should be included in your cover letter; we will change the online submission form on your behalf.

We have expanded "NIH" to "National Institutes of Health" in the Financial Disclosure, which is written in our cover letter.

Requirement 5: *Thank you for stating the following in the Competing Interests section:*

"I have read the journal's policy and the authors of this manuscript have the following competing interests: Macy Garrood, Alicia Keberle, Andria Slaughter, and Andrew McKenzie are or were employees of Sparks Brain Preservation, a non-profit brain preservation organization."

We note that one or more of the authors are employed by a commercial company: Sparks Brain Preservation

1. Please provide an amended Funding Statement declaring this commercial affiliation, as well as a statement regarding the Role of Funders in your study. If the funding organization did not play a role in the study design, data collection and analysis, decision to publish, or preparation of the manuscript and only provided financial support in the form of authors' salaries and/or research materials, please review your statements relating to the author contributions, and ensure you have specifically and accurately indicated the role(s) that these authors had in your study. You can update author roles in the Author Contributions section of the online submission form.

Please also include the following statement within your amended Funding Statement.

"The funder provided support in the form of salaries for authors [insert relevant initials], but did not have any additional role in the study design, data collection and analysis, decision to publish, or preparation of the manuscript. The specific roles of these authors are articulated in the 'author contributions' section."

If your commercial affiliation did play a role in your study, please state and explain this role within your updated Funding Statement.

We have provided amended Funding and Competing Interests Statements in the cover letter. The amended Funding Statement now includes the following: "The funder provided support in the form of salaries for authors MG, AK, AS, and ATM, but did not have any additional role in the study design, data collection and analysis, decision to publish, or preparation of the manuscript. The specific roles of these authors are articulated in the 'author contributions' section." The amended Competing Interests Statement includes: "This does not alter our adherence to PLOS ONE policies on sharing data and materials."

Requirement 6: *Please note that your Data Availability Statement is currently missing the repository name and/or the DOI/accession number of each dataset OR a direct link to access each database. If your manuscript is accepted for publication, you will be asked to provide these details on a very short timeline. We therefore suggest that you provide this information now, though we will not hold up the peer review process if you are unable.*

We have added the specific DOIs for each Zenodo repository to the Data Availability Statement.

Requirement 7: *We note that Figure 1 and S1 Data in your submission contain copyrighted images.*

We respectfully clarify that Figure 1 and S1 Data consist entirely of CT images and measurements generated by the authors as part of this study. No copyrighted material from third parties is included.

Requirement 8: *If the reviewer comments include a recommendation to cite specific previously published works, please review and evaluate these publications to determine whether they are relevant and should be cited. There is no requirement to cite these works unless the editor has indicated otherwise.*

We have evaluated the citations recommended by the reviewers and have incorporated only those ones that are directly relevant to the manuscript.

Requirement 9: *We note that you have declared the use of generative AI in the preparation of your manuscript. Please note that contributions by artificial intelligence (AI) tools and technologies to a study or to an article's contents must be clearly reported in a dedicated section of the Methods, or in the Acknowledgements section for article types lacking a Methods section. This section should include the name(s) of any tools used, a description of how the authors used the tool(s) and evaluated the validity of the tool's outputs, and a clear statement of which aspects of the study, article contents, data, or supporting files were affected/generated by AI tool usage.*

We have moved the "Use of artificial intelligence tools" section into the Methods as requested. This section specifies the tool used, describes how it was used, and how the outputs were validated.

Response to Reviewer #1

Major Points:

1-Antigenicity vs. Fixation Time: The study notes that full equilibration requires approximately 10 months. While the authors acknowledge that this period might cause some antigen masking, the manuscript would be strengthened by a more detailed discussion of which specific classes of biomolecules (e.g., lipid-anchored vs. cytosolic proteins) are most likely to be affected by this extended fluid storage prior to subzero stabilization.

We thank the reviewer for this comment. We have expanded the discussion of which biomolecular classes are most likely affected during the extended equilibration period in the Discussion: "During long-term fluid preservation in fixative, the most commonly affected biomolecular class is proteins with fixation-sensitive epitopes, whereby progressive crosslinking renders antigens increasingly inaccessible over time, while

other biomolecules such as a subset of lipids and small molecules may be extracted or chemically modified [1]. The effects have been found to be specific to the particular antigen rather than determined by subcellular localization, although there is limited data directly comparing susceptibility across subcellular compartments [1].”

2-Volumetric Analysis: The authors mention a 10% width increase in one donor (#180) and suggest it might be a measurement artifact. To meet PLOS ONE's standard for technical detail, please clarify if the measurement plane shift was confirmed by re-examining the 3D CT reconstructions.

We thank the reviewer for this comment. We performed a new analysis of the CT scan data using a sagittal approach: “To investigate this further, we performed a second analysis measuring the greatest anteroposterior brain length on sagittal CT slices, which captures an orthogonal dimension and does not require landmark identification (S2 Data). The abrupt increase seen in donor #180 was not replicated, and no consistent directional change was observed across any of the three donors. This supports the interpretation that the observed increase on coronal slice analysis was a measurement artifact rather than true swelling.” We also added to the limitations section, pointing out that 3D CT reconstruction-based analysis would be helpful: “Finally, our volumetric assessment used only two types of linear measurement on CT images, which have limited sensitivity for detecting small volume changes, and are susceptible to variability introduced by the positioning and tilt of the tissue on the scanner bed. Although this was not the primary focus of our study, future studies would benefit from larger sample sizes and more robust approaches to measuring volume changes, such as 3D reconstructions.”

Minor Points:

1-Species Generalizability: You used dog and pig brains for validation due to lower postmortem intervals. Explicitly state in the Discussion whether the denser human white matter might require even longer than the observed 10 months compared to the animal models used.

We thank the reviewer for this comment. We note that this equilibration time was observed in human brains. We expanded our discussion of potential species differences in the limitations: “For example, differences in white matter density or myelin composition between species could influence cryoprotectant diffusion kinetics, osmotic responses, and ultrastructural preservation outcomes.” (We also take this opportunity to clarify a correction made throughout the revised manuscript, which is that the previously reported figure of 10 months inadvertently included the preceding fixation period, and the cryoprotectant loading protocol alone requires approximately 9 months.)

2-Study Limitations: In line with the preference for transparency, please ensure the limitation regarding the lack of biomolecular labeling (immunoreactivity) is highlighted as a primary area for future validation, as this is the main justification for the protocol.

We thank the reviewer for this comment. We expanded our discussion of this in the limitations: “Future studies using other methods such as immunohistochemistry, RNA preservation analysis, and protein stability assays are primary areas for further validation of the molecular preservation benefits of this protocol.”

Response to Reviewer #2

1- Very Small Sample Size

• 3 human brains

• 2 dog brains

Such small sample sizes limit the statistical power and generalizability of the findings.

We thank the reviewer for this comment. We have strengthened the Limitations section to note this: “Second, our sample sizes were small. This limits the statistical power and generalizability of our findings. Larger studies will be needed to confirm these results.”

Lack of Long-Term Storage Data

Although the method is proposed for long-term preservation, the freezer storage experiment lasted only two months. This is insufficient to demonstrate the long-term stability claimed in the discussion.

We thank the reviewer for this comment. We have tempered our long-term preservation claims in the Discussion, by replacing “will maintain for many decades” with “is expected to support long-term preservation, potentially for decades”. We also added this as a Limitation: “Fifth, although our protocol closely parallels published methods that have demonstrated multi-decade preservation of fixed brain sections at -20°C (8), our freezer storage experiment lasted only two months, and further empirical validation of long-term storage in whole brains is an important area for future work.”

3. Limited Molecular Validation

The study evaluates morphological preservation, but does not test the primary motivation for the protocol: preservation of antigenicity or molecular markers.

Experiments such as:

• *immunohistochemistry*

• *RNA preservation analysis*

• *protein stability assays*

would significantly strengthen the study.

We thank the reviewer for this comment. Although additional experiments such as this are beyond the scope of the current study, we have expanded our acknowledgment of this limitation: “Fourth, we assessed only morphological preservation and did not evaluate biomolecular labeling outcomes such as immunoreactivity, which is the primary rationale for this approach over conventional room temperature or refrigerator storage. Future studies using other methods such as immunohistochemistry, RNA preservation analysis, and protein stability assays are primary areas for further validation of the molecular preservation benefits of this protocol.”

4. Mixed Species Samples

The experiments include:human brains, pig brains, dog brains

Species differences in tissue composition may influence cryoprotectant diffusion and ultrastructural preservation. This may reduce the generalizability of the results.

We thank the reviewer for this comment. We have expanded our acknowledgment of this limitation: “Third, we used donated dog tissue because it allowed for shorter postmortem intervals than we had available from human donations, providing better baseline ultrastructure for detecting any cryopreservation-induced artifacts. However, it is possible that species differences could affect the generalizability of our results. For example, differences in white matter density or myelin composition between species could influence cryoprotectant diffusion kinetics, osmotic responses, and ultrastructural preservation outcomes.”

Response to Reviewer #3

1) The full title should reflect the content, so it would logically be something like, "A method for preventing freezing damage in aldehyde fixed brains". Instead "Aldehyde-based cryopreservation of whole brains" is more easily interpreted as, using Aldehyde to cryopreserve brains.

We thank the reviewer for this comment. We considered alternative titles but believe the current title accurately reflects the content of the paper. The term “aldehyde-based cryopreservation” (ABC) describes a general strategy of combining initial aldehyde fixation with subsequent cryoprotectant loading and subzero storage, and we have used this terminology consistently throughout the manuscript. It is a previously used method that does not yet have an established term in the literature. To address the potential for misinterpretation, we have revised the wording at each introduction of the term to make the meaning of the term more clear. In the abstract, we now state: “We refer to this general strategy – aldehyde fixation followed by cryoprotectant loading and subzero storage – as aldehyde-based cryopreservation (ABC).” In the Introduction, we have similarly clarified: “We use the term aldehyde-based cryopreservation (ABC) to describe this general strategy, in which tissue that is already aldehyde fixed is subsequently cryoprotected and stored at subzero temperatures.”

2) After the abstracts introductory sentence one might expect the aim of the experiment in the second sentence "This has the potential to maintain morphology for many decades, but has been found to cause progressive loss of antigenicity over time for some biomolecules." While this problem is extensively addressed in the paper's introduction and references, no attempt in the papers methods or results is made to address it. In fact the discussion says near the end "Fourth, we assessed only morphological preservation and did not evaluate biomolecular labeling outcomes such as immunoreactivity, which is the primary rationale for this approach over conventional room temperature or refrigerator storage." So why is it so prominent in the abstract and introduction?

3) Over half the paper's introduction has emphasis on preservation of immunoreactivity as well as structure. It is not until the last portion it clarifies the two "open questions" the paper is attempting to address. "One question is the extent to which any given protocol for immersion cryoprotection may cause structural alterations as visualized by microscopy.". and "how long it takes for the solution to diffuse into the brain tissue, which determines how long of a waiting period is necessary before moving the brain to subzero storage.

We thank the reviewer for these comments. While the antigenicity issue motivates the general ABC approach, the reviewer is completely correct that this was not measured by us, nor was it the focus in this paper. Instead, the two aims of this study are (a) characterizing cryoprotectant diffusion kinetics in whole human brains and (b) assessing whether the cryoprotectant loading, subzero cooldown, rewarming, and unloading process introduces structural alterations. We therefore edited the abstract and introduction to make this more clear. For example, we removed the reference to antigenicity from the closing sentence of the abstract, which now reads: “Our approach

may be valuable for laboratories seeking a method for long-term subzero storage of fixed whole brain specimens.” In the Introduction, we also split the second paragraph into two so that the aims of our study are explained earlier, rather than appearing only in the final paragraph. The broader antigenicity rationale is still mentioned briefly in the Introduction, because we believe it helps to explain the rationale why some investigators may wish to use this method, but we attempted to make it clear that it was not a focus or an outcome of the work in our study. Additionally, the lack of additional immunoreactivity validation studies is acknowledged in the Limitations section and noted as a primary direction for future work. For more discussion of this topic, please also see our response to Reviewer #7.

4) The question posed in the introduction regarding structural preservation of the brain when certain cryprotectants are added is poorly addressed in the methods. While the blind review of micrographs is reasonably addressed, the basics of making the concentrations of cryoprotecants themselves known is poorly done. The table of concentrations should stand alone as a recipe, but using w/w and v/v without a clear indication of order or total volumes leaves it not reproducible.

We thank the reviewer for this comment. To improve clarity, we have added a sentence to the Methods specifying that sucrose was dissolved in the PBS component prior to combining with ethylene glycol, neutral buffered formalin, and glutaraldehyde. We have also specified the bath volumes for reproducibility.

Even when the supporting methods text is incorporated it is still not clear. Line 166 When was glutaraldehyde introduced previously in the loading steps?

We thank the reviewer for this comment. To make this more clear, we added an additional sentence: “Glutaraldehyde (2% v/v) was first introduced at this day 5 loading step and was maintained in all subsequent loading and unloading solutions.”

Line 176 On day 9, samples were transferred to the final solution: 50% ethylene glycol (v/v), . Is this still in 10% NBF?

We thank the reviewer for this comment. To make this more clear, we split this sentence into two: “On day 9, samples were transferred to the final solution, which contained 50% ethylene glycol (v/v), 30% sucrose (w/v), and 2% glutaraldehyde (v/v). The remaining aqueous component (38% v/v) of this final solution consisted of 10% NBF (10% v/v of the total, for a final concentration of 1% NBF) and 0.01M PBS (28% v/v of the total).”

And more fundamentally, what was the volume of the bath of cryoprotectant solutions relative to the volume of the brain?

We thank the reviewer for this comment. We added this information to the Methods section. For the whole brain portion, we added: “Given a human brain volume of approximately 1.3 L, the volume of the bath relative to the tissue volume of the brain was approximately 3:1 in step 1 (4 L solution) and 4:1 in steps 2 and 3 (5 L solution).” For the tissue biopsy portion, we added: “All solution changes for biopsy samples were performed in 20 mL conical tubes containing 10 mL of solution per sample, yielding a bath-to-tissue volume ratio of approximately 4.4:1 (10 mL solution to ~2.25 mL tissue).”

Was there any attempt to prevent the bath solution becoming diluted during the course of the experiment?

We thank the reviewer for this comment. We did not take steps to prevent dilution of the bath solution during the experiment. We agree this is a very important consideration. For clarity, we added a sentence on this to the Methods section, sub-section “Cryoprotectant penetration kinetics study”: “Solutions were not replaced or replenished within a given step.” We also added this as a new entry in the Limitations section and described possible ways to address it in future studies: “Sixth, in this study, the cryoprotectant solutions were not replenished within each loading step. As cryoprotectants diffuse from the bath into the tissue, the bath concentration will decrease over time, with the magnitude of this dilution depending on the bath-to-tissue solvent ratio and the duration of the step. This effect is most relevant for the final equilibration step, which lasted approximately 7 months. The resulting bath concentration at equilibrium would therefore be somewhat lower than the nominal starting concentration. Future studies could address this by periodically replacing the solution to maintain the nominal concentration throughout the equilibration period, and monitoring it via refractive index measurements.”

5) The use of CT scanning to track the penetration of the cryoprotectants was worth a try. Stating in the abstract "We used CT imaging to track cryoprotectant penetration, finding that with the use of our protocol, approximately 10 months is required to reach equilibration throughout whole human brains." , This totally misleading when contrasted with lines 479-486 in the discusion which indicate CT scans can't resolve the differential penetration rates of the cryoprotectants.

We thank the reviewer for this comment. We have revised the abstract to more precisely describe what the CT measurements show: “We used CT imaging to track cryoprotectant penetration, finding that approximately 9 months is required for the CT signal to stabilize throughout whole human brains.” This makes the Abstract consistent with the Discussion. (We note that we have also corrected this number from the previously reported 10 months to approximately 9 months throughout the manuscript, as the original duration had inadvertently included the preceding fixation period.)

Response to Reviewer #4

1. TITLE & ABSTRACT

• Title is clear, specific, and publishable

• Abstract is well-structured and informative

• Includes:

o Problem

o Method

o Key result (10 months equilibration)

o Validation (histology + EM)

Needs Revision

1. Too long and dense (typical PLOS ONE prefers slightly tighter abstracts)

2. Some sentences are overloaded with technical detail

3. "which is one type of a more general approach…" → wordy

4. Results could be more quantitatively emphasized

Suggested Improved Abstract

Long-term storage of aldehyde-fixed brain tissue in fluid preserves morphology but can lead to progressive loss of antigenicity. While cryoprotection has been widely used for tissue sections, its application to whole brains remains under-characterized.

Here, we present an aldehyde-based cryopreservation (ABC) protocol for intact brains using graded immersion in cryoprotectants followed by subzero storage. The protocol employs stepwise osmotic loading to a final solution containing 50% ethylene glycol and 30% sucrose.

Using CT imaging, we demonstrate that whole human brains require approximately 10 months to achieve full cryoprotectant equilibration. Insufficient equilibration resulted in ice crystal artifacts in white matter, while optimized protocols preserved cellular architecture at both light and electron microscopy levels.

This method enables long-term storage using standard laboratory freezers and offers resilience to freezer failure due to continued preservation in fluid state. The ABC approach may improve long-term antigenicity preservation in large brain specimens for research applications.

We thank the reviewer for this comment. We have made substantial edits in an attempt to improve the abstract, including condensing the part describing the initial histological findings and removing the sentence on implementation in standard laboratory freezers.

2. KEYWORDS

• *Relevant and appropriate but Add: Ultrastructure, Electron microscopy, Tissue preservation*

We thank the reviewer for this comment. We added these keywords, with the exception of “Ultrastructure” because we already have “Ultrastructural quality.”

3. INTRODUCTION

• *Excellent problem framing*

• *Clear gap identification*

• *Strong logical flow*

• *Good literature integration*

Needs Revision

a. Repetition

• *“irreplacable specimen” idea repeated → compress*

b. Some sentences too long

Example:

“Each banked brain is an irreplaceable specimen…”

→ Could be tightened

c. Aim statement slightly buried

• *Needs a clear final objective sentence*

Suggested Improved Ending Paragraph

To address these gaps, we developed and evaluated a protocol for whole-brain cryoprotectant immersion following aldehyde fixation. Using CT imaging, we quantified cryoprotectant diffusion kinetics in intact human brains. Additionally, we performed histological validation to determine whether cryoprotectant loading, storage, and unloading introduce structural artifacts. Together, these analyses provide a practical framework for long-term preservation of whole brain specimens.

We thank the reviewer for this comment. We made substantial edits to the Introduction to remove repetition, make sentences shorter, and make the aim statement more clear.

4. MATERIALS & METHODS

• *Very detailed and reproducible*

• *Strong experimental transparency*

• *Includes: Reagents, Protocol steps, Imaging, Statistics*

Needs Revision

a. Structure

• *Sections are too dense*

• *Improve readability by:*

o Adding subheadings

o Shortening paragraphs

b. Ethics Statement

• *Good, but should match journal format exactly*

Suggested Improvement (Ethics – more formal)

All animal tissues were obtained postmortem from animals euthanized for reasons unrelated to this study, with owner consent where applicable. No live animals were used; therefore, IACUC approval was not required. Human body donations were conducted under Oregon Health Authority regulations. The Apex Neuroscience Brain and Tissue Bank operates under an exemption determination from the Pearl Institutional Review Board (IRB ID #2023-0260).

We thank the reviewer for this comment. We have reviewed the Methods section and the ethics statement and made wording adjustments for clarity.

5. RESULTS

• Logical structure:

o Solution → kinetics → validation

• Strong use of:

o CT imaging

o Histology

• Excellent cause-effect reasoning

We thank the reviewer for these kind comments.

6. DISCUSSION

• Very strong section overall

• Deep Mechanistic reasoning and Literature integration

• Addresses well the Protocol rationale, Diffusion, Cryoprotectants

Needs Revision

a. Too long (major issue)

• Needs ~20–25% reduction

b. Redundancy

• Cryoprotectant explanation repeated

• It's strongly suggested to cite following most relevant studies:

https://rootspress.org/journals/agribiol/article/view/1176

https://europepmc.org/article/ppr/ppr493066

c. Some claims too strong

Example:

“will maintain for many decades”

d. Improved Version

“is expected to support long-term preservation, potentially for decades”

We thank the reviewer for these comments. We have edited the language regarding long-term preservation as suggested, revising the relevant passage to read “is expected to support long-term preservation, potentially for decades.” Regarding the suggestion to reduce the Discussion length, we reviewed the section for redundancy to make edits where possible. However, we were unable to decrease the length overall, because several other reviewers requested expanded discussion of additional topics. We have evaluated the two suggested citations and determined that they are not directly relevant to the specific topic of aldehyde-based cryopreservation of whole brains, so we have not incorporated them.

7. LIMITATIONS

• Honest and well-written

• Covers:

o Sample size

o Species differences

o Missing molecular validation

8. CONCLUSION

• Clear summary

• Matches results

Good Luck

We thank the reviewer for their many kind comments.

Response to Reviewer #5

This study addresses the critical challenge of cryopreserving whole aldehyde-fixed brains. It is the first to systematically characterize the penetration kinetics of

cryoprotective agents (CPAs) in intact human brains and resolve the core issue of ice crystal damage by optimizing the experimental protocol. The developed Aldehyde-based cryopreservation (ABC) method has the potential to preserve both tissue morphology and antigenicity, providing a novel and feasible solution for the long-term preservation of large-volume brain tissue in brain banks. The research topic is highly relevant to the practical needs of neuroscience research, with innovative and practical methodological design, relatively complete experimental data, and generally reliable conclusions. However, the study has notable limitations including small sample sizes, insufficient mechanistic exploration, and inadequate verification of practical application efficacy, which require further supplementation and improvement.

We thank the reviewer for their many kind comments. To address their first concern, we have expanded the section in the Limitations regarding the small sample size. We have also added a sentence to the Conclusion to address their other concerns as areas for future research: "Additional mechanistic characterization and practical verification of preservation efficacy will be important next steps."

Response to Reviewer #6

This manuscript presents a practical aldehyde‑based cryopreservation (ABC) protocol for fixed whole brains, combining graded cryoprotection and −20 °C storage. The authors use CT imaging to demonstrate that full equilibration in human brains takes approximately 10 months, with slower diffusion in white matter. Histological and ultrastructural analyses show that the refined protocol eliminates ice crystal artifacts and preserves tissue architecture comparably to controls. The method is simple, uses standard laboratory equipment, and offers resilience against freezer failure, making it highly valuable for brain banking. Minor revisions are needed to clarify solution composition, standardize figure labeling, and strengthen descriptions of protocol modifications. Overall, this is a rigorous, well‑designed study with clear translational impact; it is suitable for publication after these small adjustments.

We thank the reviewer for their many kind comments. Regarding solution composition and protocol modifications, we have substantially expanded the Methods section to clarify the composition of each solution and describe protocol modifications (see also our responses to Reviewer #3). Regarding figure labeling, see our responses to Reviewer #10.

Response to Reviewer #7

Reviewer #7: The manuscript "Aldehyde-based cryopreservation of whole brains" discusses the preservation of whole brains combining aldehyde-based fixation with graded immersion cryoprotection and subzero temperature storage to prevent loss of antigenicity over time for some biomolecules overtime. The paper is well written and the limitations of the study are clearly stated. To enable a thorough evaluation of the manuscript, it is essential that the authors provide data supporting their hypothesis of antigenicity preservation using this novel method. Without this data it is unclear how the integrated protocol supersedes current aldehyde based fixation protocols.

We thank the reviewer for this comment. We agree that direct experimental evidence of antigenicity preservation would strengthen the manuscript. The rationale for expecting improved antigenicity preservation with this approach is based on prior studies of similar cryoprotection-based protocols applied to fixed brain sections, which have demonstrated retention of immunoreactivity for years to decades (e.g. see references 8 and 11). Our study focused on two of the practical questions that were unresolved for applying the protocol whole brain application, i.e. (a) diffusion kinetics and (b) structural preservation through the loading/unloading process. We believe that both of these must be established before antigenicity studies on whole brains using this method can be meaningfully performed. We have revised the Abstract and Introduction of the manuscript to more clearly frame these as the aims of this paper, and to temper claims in the Discussion regarding antigenicity accordingly. We have also strengthened the Limitations section to highlight that biomolecular labeling validation (e.g., immunohistochemistry, RNA preservation, protein stability assays) is a primary area for future validation of this protocol.

Response to Reviewer #8

Revision: PONE-D-26-10146. Aldehyde-based cryopreservation of whole brains

This manuscript by Garrood and others addresses very valuable topic; the need for suitable methods of preservation of precious samples such as brains for diagnostic purposes, however, several aspects of the study design, data presentation, and overall structure limit its impact and clarity. Some sections of the manuscript would benefit from reorganization. For example, parts of the discussion are overly long and could be streamlined or partially moved to the results section to improve clarity and flow. The work doesn't seem to follow the structure of a hypothesis-driven research study. Instead, it reads more as an observational or descriptive report. While the topic is of potential interest, the manuscript would benefit from substantial revisions to improve its scientific rigor, clarity, and structure. Strengthening the statistical analysis, clarifying the

study design, and improving data presentation will be essential for supporting the conclusions. Below are my main comments and suggestions for improvement.

We thank the reviewer for this comment. We have made revisions to address the points regarding study framing, structure, statistical analysis, and data presentation, as detailed in our responses to the specific comments below.

Major Comments

1. Introduction and Rationale

The introduction does not clearly articulate the need for improving preservation methods for brain to sufficiently justify the study. The authors should better define the knowledge gap and explain why this work is necessary in the context of existing literature, and why this work is novel. I wonder if this method would also be applicable to preservation of other whole organs or tissues.

We thank the reviewer for this comment. We have revised the Introduction to more clearly articulate the knowledge gap, specifically that while cryoprotection and subzero storage has been characterized for fixed brain sections and blocks, its application to whole brains has not been well characterized, and to state the two main aims of this work more clearly. We agree with the reviewer that in future work it could be applied to other organs or tissues, and noted this in an expanded sentence in the Conclusion: “Future work should also evaluate the long-term biomolecular preservation outcomes, determine whether the protocol can be simplified without compromising tissue quality, and explore its applicability to other organs and tissues.”

2. Sample Size and Statistical Analysis

The small sample size (n = 3) is insufficient to support strong conclusions, particularly regarding volumetric changes in human brains (e.g., line 279 onward). With only one out of three samples showing changes, it is difficult to determine whether this reflects biological variability, measurement error, or an effect of the preservation method.

Additionally, there is a lack of statistical analysis for most measurements, which further limits interpretability. The only section that includes statistical treatment is the preservation score analysis of six samples at the end of the paper.

We thank the reviewer for this comment. We have strengthened the Limitations section to note this: "Second, our sample sizes were small. This limits the statistical power and generalizability of our findings. Larger studies will be needed to confirm these results." Regarding the volumetric change in donor #180, we performed an additional analysis using sagittal CT slices, which did not replicate the abrupt width increase, supporting

the interpretation that it was a measurement artifact rather than a true volume change. This has been added to the Results section.

3. Presentation of Results vs. Preliminary Observations

The initial results section (e.g., browning of brains due to glycerol) lacks supporting images and appears more like preliminary or observational data. This material may be more appropriate for inclusion in the introduction rather than the results section.

Similarly, the choice of cryoprotective solution should be justified upfront in the introduction rather than presented as a primary result since there is no experimental data for this.

We thank the reviewer for this comment. To better convey the qualitative and preliminary nature of these observations, we have renamed the section heading from "Choice of cryoprotective solution" to "Preliminary observations on cryoprotectant selection" and changed the third sentence of the paragraph to: "As an initial qualitative pilot test, we tested glycerol on a single pig brain specimen, which we reasoned would be desirable due to its long established history as a fluid preservation agent and its high viscosity."

4. Quantification of Observations

Several observations are described qualitatively. The manuscript would benefit from quantitative analyses wherever possible to strengthen the conclusions.

We thank the reviewer for this comment. We have added additional quantitative analysis, such as the Hodges-Lehmann shift and Mann-Whitney AUC calculation for the electron microscopy image data analysis. Please see our response to Reviewer #9 as well.

Minor Comments

1. Figures and Tables

o The overall quality of the figures, particularly H&E figure is very low and should be improved.

We thank the reviewer for this comment. We re-exported this at higher resolution.

o Table 1 may be more appropriate as supplementary material. Additionally, the legend refers to "MW," which is not used or explained in the table.

We thank the reviewer for this comment. We have addressed the MW issue by expanding "Average MW 40,000" to "Average Molecular Weight 40,000" within the table and removing the now-unnecessary abbreviation note from the legend. Although we agree that it could also be present in the supplementary material, we have kept Table 1 in the main text, as reagent details are important to protocol reproducibility, which was emphasized by the Editor and other reviewers.

Response to Reviewer #9

This manuscript describes an aldehyde-based cryopreservation (ABC) workflow for whole brains, combining graded immersion in cryoprotectants and storage at −20 °C. The protocol culminates in a solution of 50% (v/v) ethylene glycol and 30% (w/v) sucrose (with adjustments regarding PVP and fixatives across stages). The first part quantifies diffusion kinetics in intact human brains by CT, reporting ~10 months to achieve equilibration and noting substantially slower penetration in white matter compared with gray matter. The second part evaluates histological and ultrastructural integrity on canine brain biopsies, contrasting an initial protocol that generated white-matter artifacts (consistent with ice), with a refined protocol that eliminated those artifacts; blinded EM ratings showed no significant difference between cryopreserved and control samples (ICC = 0.76; Wilcoxon p = 0.30). The authors argue this approach can be implemented in standard freezers and may better preserve antigenicity long-term than fluid storage at higher temperatures.

Overall, the work addresses a practical and under-documented challenge in brain banking. The study is thoughtfully designed, the limitations are candidly acknowledged, and the discussion is well-informed. The principal weaknesses are (i) limited biological validation of the claimed antigenicity benefit (no immunohistochemistry is provided), (ii) the feasibility of a ~10-month equilibration for routine human brain banking, (iii) species mismatch between human (CT kinetics only) and dog (ultrastructural validation), and (iv) relatively coarse volumetric assessment during CT monitoring. These issues are addressable. I recommend Major Revision.

Major Comments

M1. Practical feasibility and optimization of diffusion timeline / The CT data in human whole brains indicate ~10 months are required to reach equilibration in the final solution, with notably slower diffusion in white matter. While compelling scientifically, this timeline is burdensome operationally for brain banks. Please (i) expand the discussion of

practical strategies to accelerate equilibration (e.g., mild elevation of temperature with biochemical risk controls, solution agitation/recirculation, intermittent partial dissection, vascular pathway facilitation post-fixation), (ii) provide estimates or pilot data on temperature-dependence for diffusion to bound plausible timelines, and (iii) clarify whether partial equilibration suffices for −20 °C storage given that ethylene glycol alone (at the stated level) may preclude ice formation before sucrose fully equilibrates.

We thank the reviewer for this comment. We agree the timeline is substantial. (We note that the figure has been corrected from the previously reported 10 months to approximately 9 months throughout the manuscript, as the original duration had inadvertently included the preceding fixation period.) We have added a paragraph to the Discussion outlining practical strategies that could be explored to accelerate equilibration: "Several strategies could also potentially shorten the equilibration period in future protocols, including a modest elevation of loading temperature (which would need to be balanced against any resulting biochemical changes), stirring the bath to keep the cryoprotectant concentration uniform at the tissue surface, sectioning of the brain prior to loading to shorten diffusion path lengths at the cost of losing whole-brain integrity, or leveraging vascular pathways to accelerate the diffusion of cryoprotectant solutions. Empirical evaluation of these strategies is an important area for future work." Regarding partial equilibration tolerance, please see our response to Q2 below.

M2. Biological validation of antigen preservation / The study's stated long-term value proposition is enhanced antigenicity retention relative to conventional fluid storage, yet no immunohistochemical (IHC) assays are reported. Given PLOS ONE's focus on methodological soundness, it would substantially strengthen the manuscript to add a minimal IHC panel (e.g., NeuN, GFAP, Iba1, synaptophysin) comparing cryopreserved vs. fluid-stored controls after representative storage intervals; if currently infeasible, provide a clear rationale and a concrete plan for future validation, and temper claims accordingly.

We thank the reviewer for this comment, which overlaps with comments from Reviewers #1, #2, and #7. We agree that direct experimental evidence of antigenicity preservation would strengthen the manuscript, although additional IHC assays are beyond the scope of the current study. We have revised the Abstract and Introduction to clarify that the aims of this paper are (a) characterizing diffusion kinetics and (b) assessing structural preservation through the loading/unloading process, rather than antigenicity preservation. We have tempered the relevant claims about antigenicity in the Discussion. We have also strengthened the relevant part of the Limitations section: "Fourth, we assessed only morphological preservation and did not evaluate biomolecular labeling outcomes such as immunoreactivity, which is the primary rationale for this approach over conventional room temperature or refrigerator storage. Future

studies using other methods such as immunohistochemistry, RNA preservation analysis, and protein stability assays are primary areas for further validation of the molecular preservation benefits of this protocol."

M3. Species and tissue-state differences / Human brains were used for CT diffusion kinetics, whereas ultrastructural validation was performed on canine biopsies with shorter PMIs and potentially distinct extracellular architectures. Please expand on the limitations in extrapolating canine biopsy outcomes to whole human brains, especially regarding white-matter packed myelinated fibers and postmortem changes that may affect permeability/osmotic responses. Consider explicitly stating that ultrastructural preservation in whole human brains remains to be demonstrated.

We thank the reviewer for this comment. We have expanded the Limitations section to point out this important limitation of our study: "Third, we used donated dog tissue because it allowed for shorter postmortem intervals than we had available from human donations, providing better baseline ultrastructure for detecting any cryopreservation-induced artifacts. However, it is possible that species differences could affect the generalizability of our results. For example, differences in white matter density or myelin composition between species could influence cryoprotectant diffusion kinetics, osmotic responses, and ultrastructural preservation outcomes. On a related note, it is important to point out that ultrastructural preservation of whole human brains following the full ABC protocol has not yet been empirically demonstrated and is a clear priority for future research."

M4. Volumetric change assessment / The manuscript reports a single linear width measure on serial CT slices and acknowledges its limitations, including a plausible landmark shift artifact. More robust volumetric or surface-based metrics (even retrospectively from existing CT datasets) would be preferable; if not possible, please strengthen the limitations and suggest concrete methods for future work (e.g., 3D segmentation, fiducial-based registration, serial photography with scale).

We thank the reviewer for this comment. This point overlaps with a comment from Reviewer #1, so please see our response there as well. We performed an additional analysis using sagittal CT slices to measure greatest anteroposterior length, which did not replicate the abrupt width increase seen in donor #180, supporting the interpretation that the original observation was a measurement artifact. We have also expanded the Limitations section to note that 3D reconstruction would be a preferable approach for future research.

M5. Freezer-failure resilience claim / The manuscript asserts that morphology would remain preserved in the fluid state upon rewarming after a freezer failure. Please qualify this claim with explicit time-at-temperature tolerances (based on prior literature or internal experience) and clarify which biomolecular features are most vulnerable during any such excursions.

We thank the reviewer for this comment. We have tempered our claims about this, by removing it from the abstract. We also added a qualifying sentence to the Discussion: "The specific time-temperature tolerances of the protocol during freezer failure, and the corresponding effects on the tissue, have not been characterized empirically and are an area for future work."

M6. Statistical power and inference / The image-level ratings were appropriately collapsed to the sample level (mean of two blinded raters per image, then averaged per specimen) and compared between groups using a two-sided Wilcoxon rank-sum test, with inter-rater reliability quantified via ICC(2,1)—an analysis plan that is methodologically sound. However, with $n = 6$ specimens per group, the study is underpowered to detect modest or even moderate differences in ultrastructural quality. Using the observed dispersion of the sample-level means (pooled SD ≈ 0.62), Monte Carlo power analyses indicate that ≈26 samples per group would be needed to achieve ~80% power for a $\Delta = 0.5$-point shift on the 1–5 scale, whereas the current design only reaches adequate power for very large effects ($\Delta \geq 1.2$). Thus, the non-significant result ($p \approx 0.30$) should not be interpreted as evidence of equivalence between conditions; rather, it reflects insufficient sensitivity to rule out practically relevant effects. I recommend (i) explicitly stating this limitation, (ii) reporting effect sizes with confidence intervals at the sample level (e.g., Hodges–Lehmann shift, AUC for Mann–Whitney), and (iii) tempering claims regarding the absence of cryopreservation-induced ultrastructural alterations. If the authors wish to exploit the full image-level dataset in future work, a hierarchical mixed-effects ordinal model (images nested within specimens; random intercepts for raters) could be considered, while retaining the specimen as the unit of inference to avoid pseudoreplication.

We thank the reviewer for this comment. We have updated the relevant Results section, including adding a Hodges-Lehmann shift and Mann-Whitney AUC to the analysis. We added the following text for clarification: "With $n = 6$ per group, the confidence intervals are wide and the study is powered only to detect large effects." We also tempered our concluding sentence to refer to the absence of a "large burden of ultrastructural artifacts," since that is all that we are able to detect with this data set.

M7. Statistical analysis transparency / The EM image scoring is clearly described, with blinded raters and ICC reporting (0.76). Please specify: (i) exact N at the level of images and samples for each condition and region, (ii) any pre-registration of the scoring rubric (if applicable), and (iii) provide per-sample distributions (e.g., in a supplemental figure) to complement the Wilcoxon summary statistics.

We thank the reviewer for this comment. (i) We have added image- and specimen-level counts per condition and region to the Methods: "Scoring was performed on the images from both grey matter (specimens c7–c9, n7–n9) and white matter (specimens c10–c12, n10–n12) samples, with three specimens per condition per region. A total of 83 cryopreserved images (37 grey matter, 46 white matter) and 83 non-cryopreserved control images (38 grey matter, 45 white matter) were scored." (ii) There was no pre-registration of the scoring rubric. We added this to the Methods: "The scoring rubric was not pre-registered." (iii) We have added a supplementary figure (S4 Data) showing per-specimen distributions of image-level scores.

M8. Citation quality and physical chemistry / Where the melting point or phase behavior of the 50% ethylene glycol solution is discussed, ensure the reference is peer-reviewed or from a handbook-grade source. If a non-peer-reviewed web resource is cited, please consider replacing or supplementing with more authoritative data and discuss the combined colligative effects of sucrose on the depression of the freezing point, acknowledging current uncertainties.

We thank the reviewer for this comment. We have changed this citation to a peer-reviewed source: "Cordray DR, Kaplan LR, Woyciesjes PM, Kozak TF. Solid-liquid phase diagram for ethylene glycol + water. Fluid Phase Equilib. 1996 Mar;117(1–2):146–52. doi:10.1016/0378-3812(95)02947-8". We note that this also led to a slight change in the reported freezing point, from -36.8°C to -38.3°C, which we also updated in the Discussion text. We updated the Discussion text about sucrose as well: "The sucrose in the solution would depress the freezing point further through colligative effects, but the magnitude is, to the best of our knowledge, not well characterized for this ternary system, nor in the context of cryopreserving brain tissue."

M9. Scope of the Discussion / The permafrost and mammoth DNA passages are interesting but somewhat speculative relative to the present data and could be condensed to keep focus on what is demonstrated here and on immediately testable extensions (e.g., −40 °C/−80 °C with alternative CPA mixtures, practical loading/unloading schedules, and IHC outcomes).

We thank the reviewer for this comment. We condensed this portion of the Discussion text, reducing the length of the text on this topic.

Minor Comments

- Terminology: Consistently define ABC early. A brief schematic of the workflow would improve readability.

We thank the reviewer for this comment. We have edited the text to ensure that ABC (aldehyde-based cryopreservation) is defined at first use and applied consistently throughout (see also our response to Reviewer #3). Regarding a schematic, we felt that the detailed step-by-step description in the Methods conveys the workflow without the need for an additional figure.

- Protocol clarity: The refined biopsy protocol includes 2% glutaraldehyde during loading/unloading. Please clarify the rationale (osmotic stabilization vs. crosslink enhancement) and whether similar inclusion is advisable or feasible for whole brains.

We thank the reviewer for this comment. We have edited the Methods to note this: “The rationale for the addition of glutaraldehyde was to reduce the osmotic reactivity of the tissue and thereby help cells to maintain their shape when exposed to and removed from hyperosmotic cryoprotectant solutions.” We also noted that we intend to add glutaraldehyde for whole brains: “Based on our biopsy-scale results, we intend to include 2% glutaraldehyde in the loading and unloading solutions for whole brains in our future research, which will at least help stabilize the tissue at the surface where cryoprotectant concentration changes occur most rapidly.”

- Figure legends: Ensure every EM panel includes scale bars in the legend and that stain/en bloc contrasting steps are unambiguously mapped to figures; the text is clear, but the figure legends could be more self-contained.

We thank the reviewer for this comment. We edited the figure legends to describe the contrasting steps more clearly and ensured that every EM panel includes the scale bars in the legend.

- White-matter artifacts, initial protocol: It would help to quantify the frequency/area fraction of voids in affected white matter (even semi-quantitatively) to support the qualitative interpretation of ice-like morphology.

We thank the reviewer for this comment. We considered quantifying the void area fraction but did not, as the initial protocol was superseded by the refined protocol and the ice-crystal interpretation rests on morphological features, such as sharp boundaries, that do not depend on quantification.

- Data availability: The manuscript references Zenodo (WSI and EM) and GitHub (code). Please provide direct DOIs and specific repository record links (not only a community URL) and ensure that raw/derived data and analysis scripts are sufficient for full reproduction of statistics and figures.

We thank the reviewer for this comment. We have added specific DOIs to ensure that the data is available for full reproduction.

- COI transparency: The ethics statement and IRB exemption are clearly stated; the competing interests note employment at a brain preservation organization. Consider adding a brief sentence in the Discussion affirming how study design, data analysis, and interpretation were insulated from these interests.

We thank the reviewer for this comment. We have added the following sentence to the Methods to make this aspect more clear: “All electron microscopy imaging was performed at core facilities, and all raw image data that was generated by them has been made publicly available (see Data Availability Statement).” Additionally, we have updated the conflict of interest section (please see Response to Journal Requirements).

- Limitations section: Already strong; consider adding a succinct note that human whole-brain ultrastructural validation (post-ABC) is a key next step.

We thank the reviewer for this comment. We added this to the Limitations section: “On a related note, it is important to point out that ultrastructural preservation of whole human brains following the full ABC protocol has not yet been empirically demonstrated and is a clear priority for future research.”

Point-by-Point Questions for the Authors

1. Equilibration feasibility: Can you provide quantitative estimates or pilot data on how modest temperature increases (e.g., 4 °C → 10 °C) or controlled agitation/recirculation would alter diffusion times for ethylene glycol and sucrose, and whether such changes introduce detectable morphological risks?

We thank the reviewer for this comment. We do not have pilot data on temperature-dependence or agitation effects for cryoprotectant diffusion in whole human brains, and generating such data would require a substantial new experimental series beyond the scope of this revision. Such computational modeling would, however, be very useful. We have noted these as priority directions for future work in the Discussion (see also our response to M1), but we are not able to provide quantitative estimates here without speculation.

2. Partial equilibration tolerance: Given that 50% ethylene glycol alone may suffice to avoid ice at −20 °C, is full sucrose equilibration necessary before freezer transfer? Could you propose decision criteria (e.g., CT thresholds) for safe transfer under partial sucrose penetration?

We thank the reviewer for this comment. We agree that 50% ethylene glycol alone is likely sufficient to prevent ice formation at -20°C, and we note this point in the Discussion in the context of our decision to remove PVP-40. However, while this is an excellent idea, we do not currently have the empirical data needed to propose specific CT thresholds for safe transfer under partial sucrose penetration. Establishing such criteria would require systematic comparison of ultrastructural outcomes across brains transferred to -20°C at varying degrees of sucrose equilibration, which is beyond the scope of this revision, but is an important direction for future work.

3. Human applicability: Do you have any preliminary biopsy-level EM from human brains after full ABC (loading → −20 °C storage → unloading) to corroborate canine findings? If not, please outline a concrete plan or acknowledge this as a priority limitation.

We thank the reviewer for this comment. We added this to the Limitations section: “On a related note, it is important to point out that ultrastructural preservation of whole human brains following the full ABC protocol has not yet been empirically demonstrated and is a clear priority for future research.”

4. IHC validation: Can you add a minimal IHC panel (NeuN, GFAP, Iba1, synaptophysin or equivalent) comparing ABC vs. conventional fluid storage after a defined interval? If not, please justify and temper claims regarding antigen preservation.

We thank the reviewer for this comment, which overlaps with M2 above. Please see our response there.

5. Statistical Power: Given the small sample size (n=6), how do you justify the claim of "no significant difference" as evidence of ultrastructural preservation rather than a lack of statistical power?

We thank the reviewer for this comment, which overlaps with M6 above. Please see our response there.

6. Unloading steps: The refined protocol shows a gradual 18-day unloading with 2% glutaraldehyde. Could you provide reasoning and any comparative data supporting the chosen decrement schedule (and whether this regimen is scalable to whole brains)?

We thank the reviewer for this comment. We have added to the methods to clarify: “The timing of the unloading schedule was chosen as a conservative reduction in step size relative to the initial protocol.” We also added to the Limitations section regarding one option for unloading cryoprotectants in whole brains: “For whole-brain applications, one practical approach would be to take biopsy samples for ultrastructural assessment rather than unloading the entire brain, given the long diffusion path lengths that would otherwise be involved.”

7. White- vs. gray-matter kinetics: Beyond qualitative CT observations, can you estimate effective diffusion coefficients (or relative rates) for white vs. gray matter, even if approximate, to aid protocol planning?

We thank the reviewer for this comment. We noted this as an area for future research in the Discussion: “Estimating effective diffusion coefficients for white versus grey matter from these CT data is an important direction for future work and would aid protocol planning.”

8. Volumetric assessment: Is it possible to perform retrospective 3D segmentation on existing CT datasets to provide volumetric change estimates (or, minimally, add orthogonal linear measurements and show their consistency)?

We thank the reviewer for this comment, which overlaps with M4 above. Please see our response there.

9. Freezer-failure resilience: Please specify time-temperature excursion limits under which morphology and antigenicity remain robust, and indicate any corrective actions (e.g., solution replacement) recommended after such events.

We thank the reviewer for this comment, which overlaps with M5 above. Please see our response there.

10. Solution composition and references: For the phase behavior of the final solution, can you provide a peer-reviewed or handbook source and discuss the combined colligative effects of sucrose with ethylene glycol (and implications for −40/−80 °C)?

We thank the reviewer for this comment, which overlaps with M8 above. Please see our response there.

11. Data and code access: Please supply permanent DOIs (not only a community page) for Zenodo datasets and ensure the GitHub repository contains scripts/notebooks sufficient to reproduce figures and statistics (including ICC and Wilcoxon analyses).

We thank the reviewer for this comment, which overlaps with one of the minor comments above ("Data availability"). Please see our response there.

12. COI safeguards: Beyond disclosure, were any safeguards in place (e.g., independent data analysis, blinded figure selection) to mitigate bias given the authors' affiliations with a brain preservation organization? A brief description would enhance transparency

We thank the reviewer for this comment, which overlaps with one of the minor comments above ("COI transparency"). Please see our response there.

Response to Reviewer #10

The paper demonstrates the optimal time of submersion in cryoprotectant solution for the adequate biobanking of whole brains, which is essential to ensure the validity of cryopreservation protocols and will surely serve as base for future research in the area. The results provided form a important stepping stone to address future refinement in cryopreservation of whole brains, with possible alternative protocols to wield better results. Even though the solid results, it is important to take a conservative view in extrapolating the results, better highlighting if the results derive from the human or dog experiments, with cautious generalization to all brains. Since the aim of the study is to present a protocol for broad usage, some minor revisions are necessary for better understanding of the procedures, as well as some methodological questions that might be valid to include in the discussion. For the questions, if the changes applied were designed to refine the protocol based on previous results and not just due to laboratory

routines, it could be relevant to explicit discuss that, emphasizing the refinement conducted along the study. Finally, some suggestions are made as final comments regarding the figures that do not impact directly in the manuscript itself but may help to improve the interpretation of the results for future readers.

We thank the reviewer for their many kind comments. Regarding the recommendation to take a conservative view in extrapolating between human and dog experiments, we have expanded the Limitations section to address this point (see also our responses to Reviewers #1, #2, and #9). Regarding the rationale for protocol modifications made during the study, we have addressed each specific change in our responses to the individual questions below.

Minor revisions

- Include glutaraldehyde and formalin in some of the descriptions of the final solution, specially because is a modification in comparison to more traditional cryopreservation solutions described in the literature.

We thank the reviewer for this comment. We agree that this should be highlighted, as it is a difference from other protocols. We have revised the abstract to describe the final solution as "50% (v/v) ethylene glycol and 30% (w/v) sucrose in fixative," which covers both the diluted neutral buffered formalin we used for whole brains, and our planned inclusion of glutaraldehyde for future whole brain storage. The rationale for including fixative as a departure from traditional formulations is already discussed in the "Cryoprotectant formulation" section of the Discussion.

- In figure 3, review the figure legend regarding which images are form the cryopreserved or non-cryopreserved group.

We thank the reviewer for this comment. We updated the figure legend to make this accurate.

- In the discussion, it said that the PVP was removed in the final protocol, which is clearly demonstrated in the methodology for the processing of the 177 donor. But in the methodology for the humans brains, the PVP is still present, making it rather unclear if it was used or not in the human brain cryopreservation. Since the main result in a protocol intended for human usage is important to specify if the removal of PVP was done only for the 177 donor, or for all the human donors as well.

We thank the reviewer for this comment. We agree that the current wording was confusing. PVP-40 was in fact used in all of the whole brain human cryoprotectant

kinetics study (donors #54, #137, and #180), and was only omitted in the refined biopsy protocol (donor #177). When we wrote that PVP was “removed from our final protocol,” we were referring to our planned protocol going forward, including for future whole-brain work, but this was not clearly stated. We have revised the Discussion to make this more clear: “Third, although PVP-40 was included in the original formulation (4) and in our whole brain cryoprotectant kinetics study, we omitted it from the refined biopsy protocol and intend to omit it from future whole-brain work as well. We found it impractical for our whole brain application, as it was difficult to prepare large volumes of solution containing it, because PVP dissolves so slowly.”

- In line 537, DMSO is directly cited with an abbreviation, without the full description before, and is also absent in the abbreviations list.

We thank the reviewer for this comment. We have updated this in the text.

Questions
- Why was PBS used in the refined protocol for the second dog brain instead of only NBF like in the previous solutions?

We thank the reviewer for this question. We have added this explanation to the Methods: “The substitution of PBS for most of the NBF was made to reduce the total formaldehyde load through the loading, storage, and unloading process, which we judged to be unnecessary at the original concentration once 2% glutaraldehyde was added earlier and maintained throughout the solution changes.”

- Why was the electron microscopy processing modified?

We thank the reviewer for this question. The EM processing was performed at a different core facility for the refined protocol samples (as noted in the Acknowledgements), and we used the enhanced-contrast NCMIR-adapted protocol that is standard at that facility, which we have found to result in slightly more crisp structures. The change in EM processing does not affect the within-experiment comparison of interest, as the cryopreserved vs. non-cryopreserved samples were processed identically.

- Why was the ethylene glycol the only cryoprotectant that had a stepwise increasing concentration? Why was not the sucrose also increased in a stepwise way?

We thank the reviewer for this question. We have clarified this in the Discussion: “Notably, of the cryoprotectants we used, only ethylene glycol was loaded in a stepwise

manner. Our reasoning is that ethylene glycol is the dominant contributor to the osmolarity of the final solution, so its gradual introduction is what primarily matters for mitigating osmotic stress during loading. Sucrose was introduced only at the final step, by which point the tissue is already loaded with a high concentration of ethylene glycol, such that the marginal osmotic contribution from adding sucrose would be secondary."

- In the results, the authors say that the human brains were easily able to be sectioned for extracting a biopsy sample, was any tissue extracted? If so, was it submitted to any histological evaluation like the procedures done to the dogs brains? If not, it may be important to include this lack of analysis in the limitation section.

We thank the reviewer for this question. No tissue from the human brains was submitted for histological evaluation in this study. We have added this point to the Limitations section: "Although the human brain tissue in the present study was sectioned to confirm that it could be cleanly cut, no tissue from these brains was submitted for histological or ultrastructural evaluation, and doing so is a clear next step."

- Regarding the volumetric change observed in one of the human brain, is there any medical condition from the donor that might contribute to this alteration?

We thank the reviewer for this question. We are not aware of any medical condition in this donor that would account for the observed change. We would also note that, as discussed in our response to Reviewer #1, we performed an additional analysis using sagittal CT slices to measure anteroposterior brain length, which did not replicate the abrupt width increase seen in donor #180. This supports the interpretation that the original observation reflects a measurement artifact, rather than true tissue swelling.

Suggestions
- In figure 1, add the solution change in each ramp up, to a better visualisation of the stepping protocol.

We thank the reviewer for this comment. Instead of adding it to the figure, we have included this in the figure legend. We think that adding all of that text to the figure might make it too crowded and distracting for readers.

- In figure 3, add a title above the panels to indicate the groups, similar to the previous figures.

We thank the reviewer for this comment. We have updated this figure as suggested.

- In figures 2 and 4, add the donor number in the figure legend.

We thank the reviewer for this comment. We have added the donor numbers to the figure legends.

- If possible, adding images of the browning of the pig and human brains in the supplementary material may be interesting for visualisation of the process.

We thank the reviewer for this suggestion. We have added a photograph of the human brains showing the browning effect as supplementary material (S3 Data). Unfortunately, we do not have a comparable photograph of the pig brain available.
