# Supplementary material for: Cryopreservation of aldehyde-fixed whole brains
Source: PLoS One. 2026 Aug 24;21(8):e0344932. doi: 10.1371/journal.pone.0344932 (PMC13502592; doi:10.1371/journal.pone.0344932)
Supplement: Supplementary file 5 [file pone.0344932.s005.docx]

### 1- Very Small Sample Size

- 3 human brains
- 2 dog brains

Such small sample sizes limit the statistical power and generalizability of the findings.

### 2. Lack of Long-Term Storage Data

Although the method is proposed for **long-term preservation**, the freezer storage experiment lasted only **two months**. This is insufficient to demonstrate the long-term stability claimed in the discussion.

### 3. Limited Molecular Validation

The study evaluates **morphological preservation**, but does not test the primary motivation for the protocol: preservation of **antigenicity** or molecular markers.

Experiments such as:

- immunohistochemistry
- RNA preservation analysis
- protein stability assays

would significantly strengthen the study.

### 4. Mixed Species Samples

The experiments include:human brains, pig brains, dog brains

Species differences in tissue composition may influence cryoprotectant diffusion and ultrastructural preservation. This may reduce the generalizability of the results.

### 5. Diffusion Time May Be Impractical

The protocol requires approximately **10 months** for full equilibration of cryoprotectants in whole human brains. Such a long preparation period may limit the practical adoption of the method in some brain banks
