# Supplementary material for: Cryopreservation of aldehyde-fixed whole brains
Source: PLoS One. 2026 Aug 24;21(8):e0344932. doi: 10.1371/journal.pone.0344932 (PMC13502592; doi:10.1371/journal.pone.0344932)
Supplement: Supplementary file 6 [file pone.0344932.s006.docx]

1. **TITLE & ABSTRACT**

- Title is **clear, specific, and publishable**
- Abstract is **well-structured and informative**
- Includes:
  - Problem
  - Method
  - Key result (10 months equilibration)
  - Validation (histology + EM)

**Needs Revision**

1. **Too long and dense** (typical PLOS ONE prefers slightly tighter abstracts)
2. Some sentences are **overloaded with technical detail**
3. “which is one type of a more general approach…” → wordy
4. Results could be more **quantitatively emphasized**

**Suggested Improved Abstract**

Long-term storage of aldehyde-fixed brain tissue in fluid preserves morphology but can lead to progressive loss of antigenicity. While cryoprotection has been widely used for tissue sections, its application to whole brains remains under-characterized.

Here, we present an aldehyde-based cryopreservation (ABC) protocol for intact brains using graded immersion in cryoprotectants followed by subzero storage. The protocol employs stepwise osmotic loading to a final solution containing 50% ethylene glycol and 30% sucrose.

Using CT imaging, we demonstrate that whole human brains require approximately 10 months to achieve full cryoprotectant equilibration. Insufficient equilibration resulted in ice crystal artifacts in white matter, while optimized protocols preserved cellular architecture at both light and electron microscopy levels.

This method enables long-term storage using standard laboratory freezers and offers resilience to freezer failure due to continued preservation in fluid state. The ABC approach may improve long-term antigenicity preservation in large brain specimens for research applications.

2. **KEYWORDS**

- Relevant and appropriate but Add: *Ultrastructure*, *Electron microscopy*, *Tissue preservation*

3. **INTRODUCTION**

- Excellent **problem framing**
- Clear **gap identification**
- Strong **logical flow**
- Good literature integration

**Needs Revision**

a. **Repetition**

- “irreplacable specimen” idea repeated → compress

b. **Some sentences too long**

Example:

“Each banked brain is an irreplaceable specimen…”

→ Could be tightened

c. **Aim statement slightly buried**

- Needs a **clear final objective sentence**

**Suggested Improved Ending Paragraph**

To address these gaps, we developed and evaluated a protocol for whole-brain cryoprotectant immersion following aldehyde fixation. Using CT imaging, we quantified cryoprotectant diffusion kinetics in intact human brains. Additionally, we performed histological validation to determine whether cryoprotectant loading, storage, and unloading introduce structural artifacts. Together, these analyses provide a practical framework for long-term preservation of whole brain specimens.

4. **MATERIALS & METHODS**

- Very detailed and reproducible
- Strong **experimental transparency**
- Includes: Reagents, Protocol steps, Imaging, Statistics

**Needs Revision**

a. **Structure**

- Sections are **too dense**
- Improve readability by:
  - Adding **subheadings**
  - Shortening paragraphs

b. **Ethics Statement**

- Good, but **should match journal format exactly**

**Suggested Improvement (Ethics – more formal)**

All animal tissues were obtained postmortem from animals euthanized for reasons unrelated to this study, with owner consent where applicable. No live animals were used; therefore, IACUC approval was not required. Human body donations were conducted under Oregon Health Authority regulations. The Apex Neuroscience Brain and Tissue Bank operates under an exemption determination from the Pearl Institutional Review Board (IRB ID #2023-0260).

5. **RESULTS**

- Logical structure:
  - Solution → kinetics → validation
- Strong use of:
  - CT imaging
  - Histology
- Excellent **cause-effect reasoning**

6. **DISCUSSION**

- Very strong section overall
- Deep Mechanistic reasoning and Literature integration
- Addresses well the Protocol rationale, Diffusion, Cryoprotectants

**Needs Revision**

a. **Too long (major issue)**

- Needs **~20–25% reduction**

b. **Redundancy**

- Cryoprotectant explanation repeated
- It’s strongly suggested to cite following most relevant studies:
- <https://rootspress.org/journals/agribiol/article/view/1176>
- <https://europepmc.org/article/ppr/ppr493066>

c. **Some claims too strong**

Example:

“will maintain for many decades”

d. **Improved Version**

“is expected to support long-term preservation, potentially for decades”

7. **LIMITATIONS**

- Honest and well-written
- Covers:
  - Sample size
  - Species differences
  - Missing molecular validation

8. **CONCLUSION**

- Clear summary
- Matches results

*Good Luck*
