# Supplementary material for: Cryopreservation of aldehyde-fixed whole brains
Source: PLoS One. 2026 Aug 24;21(8):e0344932. doi: 10.1371/journal.pone.0344932 (PMC13502592; doi:10.1371/journal.pone.0344932)
Supplement: Supplementary file 7 [file pone.0344932.s007.docx]

# Review Comments: Aldehyde-based cryopreservation of whole brains

**Manuscript ID**: PONE-D-26-10146

## I. Overall Evaluation

This study addresses the critical challenge of cryopreserving whole aldehyde-fixed brains. It is the first to systematically characterize the penetration kinetics of cryoprotective agents (CPAs) in intact human brains and resolve the core issue of ice crystal damage by optimizing the experimental protocol. The developed Aldehyde-based cryopreservation (ABC) method has the potential to preserve both tissue morphology and antigenicity, providing a novel and feasible solution for the long-term preservation of large-volume brain tissue in brain banks. The research topic is highly relevant to the practical needs of neuroscience research, with innovative and practical methodological design, relatively complete experimental data, and generally reliable conclusions. However, the study has notable limitations including small sample sizes, insufficient mechanistic exploration, and inadequate verification of practical application efficacy, which require further supplementation and improvement.

## II. Key Innovations

1. **Methodological Innovation**: Combining aldehyde fixation with graded CPA loading, the study optimized a CPA formulation consisting of 50% (v/v) ethylene glycol and 30% (w/v) sucrose, abandoning glycerol (prone to tissue browning) and PVP-40 (difficult to dissolve). This formulation fulfills multiple requirements: anti-ice crystal formation at subzero temperatures, microbial growth inhibition during liquid storage, and long-term morphological stability of tissue.
2. **Technical Breakthrough**: Using CT imaging to quantitatively track CPA penetration in intact human brains, the study for the first time confirmed that **approximately 10 months** are required for complete CPA equilibration in whole human brains, with a significantly slower penetration rate in white matter than in gray matter. This provides quantitative evidence for formulating protocols for whole-brain cryopreservation.
3. **Practical Value**: The optimized protocol can be implemented with standard laboratory freezers and confers resilience against freezer failure—fixed tissue can maintain long-term morphological integrity in the fluid state even if rewarmed. This adaptability to the hardware conditions of ordinary brain banks ensures its ease of popularization and application.
4. **Comprehensive Validation System**: Multi-dimensional verification of preservation efficacy from tissue architecture to ultrastructure was conducted via light microscopy, electron microscopy, and blinded scoring (intraclass correlation coefficient, ICC=0.76, indicating good interrater reliability). The results confirmed that the optimized protocol completely eliminates ice crystal damage in white matter, with preservation efficacy not significantly different from non-cryopreserved controls.

## III. Critical Issues and Revision Suggestions

### (I) Experimental Design and Sample Size Limitations

1. **Small and homogeneous human sample size**: Only 3 human brain samples were included in the penetration kinetics study, all from elderly donors (71, 75, and 90+ years old). There is a lack of samples from young and middle-aged donors and those with different brain pathologies (e.g., Alzheimer’s disease, cerebrovascular disease), making it impossible to verify whether CPA penetration rate is affected by age or pathological changes (e.g., brain atrophy, gliosis).**Suggestion**: Supplement 2-3 human brain samples of different ages and pathological types; or clearly state the sample limitations in the Discussion and note that subsequent studies need to expand the sample size to verify the generalizability of the penetration kinetics.
2. **Need for further justification of animal model selection**: Canine brains (rather than non-human primate brains, which are more similar to humans) were used for histological validation. Although the study mentions that canine brains have shorter postmortem intervals and better baseline ultrastructure, it does not compare the differences in white matter density and myelin structure between canine and human brains.**Suggestion**: Add a comparison of relevant histological characteristics between canine and human brains in the Materials and Methods section; or indicate in the Discussion that validation with non-human primate models is a key direction for future research.
3. **Unverified long-term preservation efficacy**: Only short-term (days to months) cryopreservation and rewarming experiments were conducted, with no assessment of tissue morphology and antigenicity changes after storage at -20°C for 1 year or longer—**long-term preservation** is the core objective of this study.**Suggestion**: Supplement long-term cryopreservation validation experiments for at least 6 months and 1 year, detecting tissue ultrastructure and immunoreactivity of key antigens (e.g., NeuN, GFAP); or clearly state in the Discussion that long-term preservation efficacy is a core to-be-verified point of this method.

### (II) Insufficient Methodological and Mechanistic Exploration

1. **Unelucidated molecular mechanism of CPA penetration**: The study only observed slower CPA penetration in white matter than in gray matter and speculated that lipid-rich myelin sheaths impede the diffusion of water-soluble CPAs, but no experimental verification was performed (e.g., correlating myelin content with CPA penetration rate).**Suggestion**: Supplement relevant molecular experiments or literature evidence to clarify the influence of myelin, cell density, and other factors on CPA diffusion; or delete the unvalidated speculation and only objectively describe the experimental phenomena.
2. **Lack of single-factor verification for protocol optimization**: Three factors were adjusted simultaneously in the optimized protocol (**equilibration time, unloading gradient, and glutaraldehyde addition timing**). No single-factor experiments were conducted to identify the key factor eliminating ice crystal damage.**Suggestion**: Supplement single-factor gradient experiments to clarify the independent role of each optimization measure, providing a basis for further simplification of the protocol (e.g., whether equilibration time can be appropriately shortened).
3. **Crude method for assessing volume changes**: Brain volume changes were only evaluated by measuring the maximum transverse diameter at the level of the frontal horns on coronal CT images, which is susceptible to CT scanning slice deviation and artifacts caused by CPA filling in the ventricles.**Suggestion**: Adopt 3D reconstruction technology for quantitative volumetric analysis of the whole brain, supplement volume change data of different brain regions (gray matter, white matter, ventricles), and improve the accuracy of results.

### (III) Inadequate Result Demonstration and Data Presentation

1. **Unverified antigenicity preservation efficacy**: The core advantage of the ABC method proposed in the study is **reducing antigenicity loss**, but the entire manuscript only verifies morphological and ultrastructural preservation without detecting the immunoreactivity of any proteins, which is inconsistent with the research background and objectives.**Suggestion**: Supplement immunohistochemical/immunoelectron microscopy experiments for key markers (neuronal, glial, and synapse-related proteins), and compare the antigenicity preservation efficacy of the ABC method with traditional aldehyde-based liquid storage and conventional cryopreservation to provide core evidence for the research conclusions.
2. **Insufficient quantitative analysis of CT imaging**: The stabilization of Hounsfield Unit (HU) values was only used as the criterion for equilibration, without quantitative analysis of the HU value change trends and equilibrium time differences in different brain regions (cortex, thalamus, basal ganglia, white matter).**Suggestion**: Supplement dynamic quantitative data of HU values in each brain region, plot penetration kinetic curves, and clarify the equilibrium time window for different brain regions.
3. **Need for improved statistical analysis**: Some results only describe qualitative phenomena (e.g., "no obvious volume changes") without statistical tests; the Wilcoxon rank-sum test was only used to compare ultrastructural scores between two groups, with no analysis of quantitative data from multiple time points and multiple brain regions.**Suggestion**: Perform appropriate statistical tests (e.g., repeated measures analysis of variance, multiple comparisons) on all quantitative data (e.g., HU values, brain volume, ultrastructural scores), and clearly state the statistical methods and significance level in the figure legends.

### (IV) Practical Application and Protocol Feasibility Issues

1. **Need to explore the practical feasibility of the 10-month equilibration period**: The 10-month equilibration time required for complete CPA penetration in whole human brains is excessively long, which may increase the storage cost and operational difficulty of brain banks.**Suggestion**: Supplement potential strategies for optimizing the equilibration time in the Discussion (e.g., moderately increasing temperature, combining mild perfusion-assisted penetration) and analyze their feasibility and potential risks; simultaneously evaluate the impact of shortening the equilibration time on preservation efficacy to provide a compromise for practical application.
2. **Unspecified bulk preparation and stability of CPAs**: The CPA used in the study is a mixed formulation, but the methods for bulk preparation (suitable for whole brains), storage conditions, and stability (e.g., whether solute precipitation or ethylene glycol volatilization occurs) are not described.**Suggestion**: Supplement the bulk preparation process and stability test results of CPAs in the Materials and Methods section (e.g., component changes after 10 months of storage at 4°C) to improve the repeatability of the protocol.
3. **Unclear operational details of rewarming and CPA unloading**: The optimized 18-day gradient CPA unloading protocol lacks detailed operational information such as temperature control, solution replacement frequency, and light protection requirements.**Suggestion**: Supplement complete operational procedures and clarify key operational parameters to ensure reproducibility in other laboratories.

### (V) Discussion and Limitation Analysis

1. **Insufficient comparison with existing whole-brain preservation methods**: No systematic comparison was made between the ABC method and existing large-volume brain tissue preservation methods (e.g., perfusion-based cryoprotection, ultra-low temperature cryopreservation) in terms of **preservation efficacy, operational difficulty, cost, and time consumption**.**Suggestion**: Add a comparison table in the Discussion to clarify the advantages and disadvantages of the ABC method, providing a reference for method selection in brain banks.
2. **Incomplete limitation analysis**: Only sample size, species differences, and the crudeness of volume assessment methods were mentioned as limitations, with no reference to core limitations such as **unverified antigenicity, lack of long-term preservation efficacy, and excessively long equilibration period**.**Suggestion**: Supplement a comprehensive limitation analysis, objectively point out the unresolved problems of the study, and clearly define the key directions for future research.

## IV. Minor Revision and Format Suggestions

1. **Figure and Table Optimization**: The legends of Fig 2 and Fig 4 are unclear, with no specific values for scale bars; S1 Data only provides transverse diameter measurement data, without original CT images and statistical analysis results.**Suggestion**: Improve figure and table annotations, and supplement original images and statistical analysis plots (e.g., box plots, line charts).
2. **Unification of Abbreviations and Terminology**: Inconsistent capitalization (e.g., PVP-40/Pvp) and unannotated first occurrences of abbreviations (e.g., WSI) are present in the manuscript.**Suggestion**: Unify the format of terminology and provide full names for all abbreviations at their first occurrence.
3. **Reference Supplementation**: Some key viewpoints (e.g., the anti-ice crystal mechanism of ethylene glycol, osmotic sensitivity of aldehyde-fixed tissue) are supported by relatively outdated references.**Suggestion**: Supplement relevant research literature from the past 5 years to enhance the timeliness and reliability of the viewpoints.
4. **Author Contributions and Competing Interests**: The competing interests section mentions that some authors are employees of a non-profit brain preservation organization but does not explain the specific association between the organization and the study.**Suggestion**: Further clarify the specific content of competing interests to ensure transparency.

## V. Review Conclusion

This study has important methodological innovation and practical application value, and the developed ABC method provides a new feasible solution for whole-brain long-term preservation. The experimental design is basically rational with relatively complete data. However, there are core issues including **unverified antigenicity, small sample size, lack of long-term preservation efficacy, and insufficient mechanistic exploration**, which require **major revisions** to supplement key experimental data and analyses, and improve methodological details and result demonstration.

The manuscript may be considered for acceptance after the above core issues are resolved. If key experiments (e.g., long-term preservation, antigenicity detection) cannot be supplemented, the study limitations should be clearly stated in the Discussion, and the research conclusion should be repositioned as "a feasible method for short-term cryopreservation of whole brains, with long-term efficacy to be further verified".

**Reviewer’s Recommendation**: Revise majorly and resubmit for re-review
