# Supplementary material for: Cryopreservation of aldehyde-fixed whole brains
Source: PLoS One. 2026 Aug 24;21(8):e0344932. doi: 10.1371/journal.pone.0344932 (PMC13502592; doi:10.1371/journal.pone.0344932)
Supplement: Supplementary file 8 [file pone.0344932.s009.docx]

**Response to Reviewers**

We thank the Editor and the Reviewers for their positive and constructive comments, which have greatly improved the manuscript. Below, we provide point-by-point responses to the comments from Reviewer #3.

*The authors have improved their manuscript considerably. Some might be interested to know how long it takes for cryo-protectants to penetrate fixed brain, even if the paper in only indicative rather than precise about the process.*

*There are still two major problems already raised that have not been addressed at all or not fully addressed.*

*1)The title is still aimed at being an expanded acronym rather than descriptive of the paper’s content. As much as ABC for Aldehyde-based cryopreservation .... might be useful as a short cut descriptor in a lab it does not appropriately reflect the contents of the paper. There is no “Aldehyde-based cryopreservation” of whole brains in the paper. There is cryopreservation of aldehyde fixed brains, a significantly different meaning. To sacrifice the meaning of the paper’s title for the sake of fitting an illogical acronym is more than inconcise. It is significantly flawed paper writing style. Running against Plone’s requirements for a title ‘Titles must be specific, descriptive, concise, and understandable to readers outside your exact field’.*

We thank the reviewer for this comment. We agree with the reviewer. To make the title more accurately reflect the contents of the paper, we have changed the title to “Cryopreservation of aldehyde-fixed whole brains”.

*2)While the formulae used for cryopreservatives have been enourmously improved they still appear incomplete and technically non-reproducible without assumptions being made. Around line 198 Formula in the text and table 2 seem to assume incorrectly that 30% sucrose w/v adds no volume to the cryopreservatives? Table 2 40%+ 30%+ 28%+ 2%= 100% plus 25% sucrose =125% for example? While not directly proportional it does add at least 13% to the volume. How is this accounted for?*

We thank the reviewer for this comment. This comment prompted us to revisit our original solution preparation records, which led us to identify four errors in the manuscript that have now been corrected.

First, for both the loading and unloading portions of the refined protocol, the volume contribution of dissolved sucrose was not accounted for during solution preparation. Because the final solution volume was not measured after sucrose dissolution, the exact final concentrations cannot be determined retrospectively. Therefore, we added clarifications throughout the Methods that the reported concentrations should be regarded as nominal concentrations based on the intended formulation rather than measured final solution volumes. For example, when describing the loading protocol we noted: “Notably, the additional volume contributed by the dissolved sucrose after adding it to PBS was not accounted for, so these percentages should be considered nominal concentrations, because they are based on the intended formulation rather than the measured final solution volume.” We also added a paragraph on this to the Limitations section: “Seventh, in the refined protocol for cryoprotectant loading and unloading of samples, the volume contribution of the dissolved sucrose in PBS was not accounted for when preparing the cryoprotectant solutions. As a result, the reported concentrations are nominal rather than true final concentrations, and the actual concentrations of the solution components were slightly lower than stated for those solutions.”

Second, we determined that for the whole brain protocol and the initial protocol of biopsy sample cryoprotection, the sucrose and PVP were dissolved in the ethylene glycol component of the solution rather than in NBF. This error has been corrected.

Third, we identified an error in the reported composition of the final cryoprotectant loading solution. The percentage of PBS was incorrectly listed as 28% rather than 38%. This error has been corrected.

Fourth, in reviewing our preparation records in response to the reviewer's comments, we discovered that the manuscript had incorrectly described the glutaraldehyde concentration. The protocol used 2% (v/v) of a 50% glutaraldehyde stock solution, corresponding to a final glutaraldehyde concentration of 1%, rather than 2%. This error has been corrected.

*A minor point. In terms of clarity of the somewhat lengthy sentences at times, one that seems worth revising:*

*183 "The substitution of PBS for most of the NBF was made to reduce the total formaldehyde load through the loading, storage, and unloading process, which we judged to be unnecessary at the original concentration once 2% glutaraldehyde was added earlier and maintained throughout the solution changes."*

*The sentence so long it becomes confusing. The subject of the sentence is changed without making it clear which subjects are being referred to at any one point. I presume it means :*

*The substitution of PBS for most of the NBF was made to reduce the total formaldehyde load through the loading, storage, and unloading processes. We judged formaldehyde unnecessary at the original concentration due to the effect of adding 2% glutaraldehyde earlier in this protocol than in other protocols, and maintaining glutaraldehyde throughout the solution changes.*

We thank the reviewer for this comment. We have adopted the reviewer’s suggested revision for this sentence, which we agree makes it clearer.
