# Supplementary material for: Cryopreservation of aldehyde-fixed whole brains
Source: PLoS One. 2026 Aug 24;21(8):e0344932. doi: 10.1371/journal.pone.0344932 (PMC13502592; doi:10.1371/journal.pone.0344932)
Supplement: Peer review history

# Peer review: Decision Letter 0

**DOI:** 10.1371/journal.pone.0344932.r001

**Authors:** Ghadeer Sabah Bustani

24 Apr 2026

Aldehyde-based cryopreservation of whole brains

PLOS One

Dear Dr. McKenzie,

Thank you for submitting your manuscript to PLOS ONE. After careful consideration, we feel that it has merit but does not fully meet PLOS ONE’s publication criteria as it currently stands. Therefore, we invite you to submit a revised version of the manuscript that addresses the points raised during the review process.

Please submit your revised manuscript by Jun 07 2026 11:59PM. If you will need more time than this to complete your revisions, please reply to this message or contact the journal office at plosone@plos.org. When you're ready to submit your revision, log on to https://www.editorialmanager.com/pone/ and select the 'Submissions Needing Revision' folder to locate your manuscript file.

Please include the following items when submitting your revised manuscript:

- A letter that responds to each point raised by the academic editor and reviewer(s). You should upload this letter as a separate file labeled 'Response to Reviewers'.
- A marked-up copy of your manuscript that highlights changes made to the original version. You should upload this as a separate file labeled 'Revised Manuscript with Track Changes'.
- An unmarked version of your revised paper without tracked changes. You should upload this as a separate file labeled 'Manuscript'.

If you would like to make changes to your financial disclosure, please include your updated statement in your cover letter. Guidelines for resubmitting your figure files are available below the reviewer comments at the end of this letter.

If applicable, we recommend that you deposit your laboratory protocols in protocols.io to enhance the reproducibility of your results. Protocols.io assigns your protocol its own identifier (DOI) so that it can be cited independently in the future. For instructions see: https://journals.plos.org/plosone/s/submission-guidelines#loc-laboratory-protocols. Additionally, PLOS ONE offers an option for publishing peer-reviewed Lab Protocol articles, which describe protocols hosted on protocols.io. Read more information on sharing protocols at https://plos.org/protocols?utm_medium=editorial-email&utm_source=authorletters&utm_campaign=protocols.

As the corresponding author, your ORCID iD is verified in the submission system and will appear in the published article. PLOS supports the use of ORCID, and we encourage all coauthors to register for an ORCID iD and use it as well. Please encourage your coauthors to verify their ORCID iD within the submission system before final acceptance, as unverified ORCID iDs will not appear in the published article. *Only*  the individual author can complete the verification step; PLOS staff *cannot*  verify ORCID iDs on behalf of authors.

We look forward to receiving your revised manuscript.

Kind regards,

Ghadeer Sabah Bustani, Ph.D

Academic Editor

PLOS One

Journal Requirements:

When submitting your revision, we need you to address these additional requirements.

1.Please ensure that your manuscript meets PLOS ONE's style requirements, including those for file naming. The PLOS ONE style templates can be found at

https://journals.plos.org/plosone/s/file?id=wjVg/PLOSOne_formatting_sample_main_body.pdf and

https://journals.plos.org/plosone/s/file?id=ba62/PLOSOne_formatting_sample_title_authors_affiliations.pdf

2. Please note that funding information should not appear in any section or other areas of your manuscript. We will only publish funding information present in the Funding Statement section of the online submission form. Please remove any funding-related text from the manuscript.

3. We note that the grant information you provided in the ‘Funding Information’ and ‘Financial Disclosure’ sections do not match.

When you resubmit, please ensure that you provide the correct grant numbers for the awards you received for your study in the ‘Funding Information’ section.

4. Please expand the acronym “NIH” (as indicated in your financial disclosure) so that it states the name of your funders in full.

This information should be included in your cover letter; we will change the online submission form on your behalf.

5. Thank you for stating the following in the Competing Interests section:

“I have read the journal's policy and the authors of this manuscript have the following competing interests: Macy Garrood, Alicia Keberle, Andria Slaughter, and Andrew McKenzie are or were employees of Sparks Brain Preservation, a non-profit brain preservation organization.”

We note that one or more of the authors are employed by a commercial company: Sparks Brain Preservation

1. Please provide an amended Funding Statement declaring this commercial affiliation, as well as a statement regarding the Role of Funders in your study. If the funding organization did not play a role in the study design, data collection and analysis, decision to publish, or preparation of the manuscript and only provided financial support in the form of authors' salaries and/or research materials, please review your statements relating to the author contributions, and ensure you have specifically and accurately indicated the role(s) that these authors had in your study. You can update author roles in the Author Contributions section of the online submission form.

Please also include the following statement within your amended Funding Statement.

“The funder provided support in the form of salaries for authors [insert relevant initials], but did not have any additional role in the study design, data collection and analysis, decision to publish, or preparation of the manuscript. The specific roles of these authors are articulated in the ‘author contributions’ section.”

If your commercial affiliation did play a role in your study, please state and explain this role within your updated Funding Statement.

2. Please also provide an updated Competing Interests Statement declaring this commercial affiliation along with any other relevant declarations relating to employment, consultancy, patents, products in development, or marketed products, etc.

Within your Competing Interests Statement, please confirm that this commercial affiliation does not alter your adherence to all PLOS ONE policies on sharing data and materials by including the following statement: "This does not alter our adherence to  PLOS ONE policies on sharing data and materials.” (as detailed online in our guide for authors http://journals.plos.org/plosone/s/competing-interests) . If this adherence statement is not accurate and  there are restrictions on sharing of data and/or materials, please state these. Please note that we cannot proceed with consideration of your article until this information has been declared.

Please include both an updated Funding Statement and Competing Interests Statement in your cover letter. We will change the online submission form on your behalf.

6. Please note that your Data Availability Statement is currently missing the repository name and/or the DOI/accession number of each dataset OR a direct link to access each database. If your manuscript is accepted for publication, you will be asked to provide these details on a very short timeline. We therefore suggest that you provide this information now, though we will not hold up the peer review process if you are unable.

7. We note that Figure 1 and S1 Data in your submission contain copyrighted images. All PLOS content is published under the Creative Commons Attribution License (CC BY 4.0), which means that the manuscript, images, and Supporting Information files will be freely available online, and any third party is permitted to access, download, copy, distribute, and use these materials in any way, even commercially, with proper attribution. For more information, see our copyright guidelines: http://journals.plos.org/plosone/s/licenses-and-copyright.

We require you to either (1) present written permission from the copyright holder to publish these figures specifically under the CC BY 4.0 license, or (2) remove the figures from your submission:

1. You may seek permission from the original copyright holder of Figure 1 and S1 Data to publish the content specifically under the CC BY 4.0 license.

We recommend that you contact the original copyright holder with the Content Permission Form (http://journals.plos.org/plosone/s/file?id=7c09/content-permission-form.pdf) and the following text:

“I request permission for the open-access journal PLOS ONE to publish XXX under the Creative Commons Attribution License (CCAL) CC BY 4.0 (http://creativecommons.org/licenses/by/4.0/). Please be aware that this license allows unrestricted use and distribution, even commercially, by third parties. Please reply and provide explicit written permission to publish XXX under a CC BY license and complete the attached form.”

Please upload the completed Content Permission Form or other proof of granted permissions as an "Other" file with your submission.

In the figure caption of the copyrighted figure, please include the following text: “Reprinted from [ref] under a CC BY license, with permission from [name of publisher], original copyright [original copyright year].”

2. If you are unable to obtain permission from the original copyright holder to publish these figures under the CC BY 4.0 license or if the copyright holder’s requirements are incompatible with the CC BY 4.0 license, please either i) remove the figure or ii) supply a replacement figure that complies with the CC BY 4.0 license. Please check copyright information on all replacement figures and update the figure caption with source information. If applicable, please specify in the figure caption text when a figure is similar but not identical to the original image and is therefore for illustrative purposes only.

8. If the reviewer comments include a recommendation to cite specific previously published works, please review and evaluate these publications to determine whether they are relevant and should be cited. There is no requirement to cite these works unless the editor has indicated otherwise.

9. We note that you have declared the use of generative AI in the preparation of your manuscript. Please note that contributions by artificial intelligence (AI) tools and technologies to a study or to an article’s contents must be clearly reported in a dedicated section of the Methods, or in the Acknowledgements section for article types lacking a Methods section. This section should include the name(s) of any tools used, a description of how the authors used the tool(s) and evaluated the validity of the tool’s outputs, and a clear statement of which aspects of the study, article contents, data, or supporting files were affected/generated by AI tool usage (https://journals.plos.org/plosone/s/ethical-publishing-practice#loc-artificial-intelligence-tools-and-technologies).

Please therefore revise your manuscript to provide all necessary information to meet these requirements. We appreciate your attention to these queries and look forward to your response.

Additional Editor Comments:

Dear Authors, thank you for submitting your manuscript. While the topic is important and potentially valuable, the current version has several significant limitations that affect its scientific rigor and clarity; therefore, a decision of Major Revision has been made. The study lacks a clear hypothesis-driven design, and the small sample size (n = 3) combined with the absence of adequate statistical analysis weakens the reliability of the conclusions. Additionally, the introduction does not clearly define the knowledge gap or novelty, and there is inconsistency between the stated aims and the methods and results presented. Many findings are described qualitatively without sufficient quantification, and the overall structure—particularly the distinction between Results and Discussion—requires reorganization. Methodological details are also insufficient to ensure reproducibility, and the quality of figures and tables needs improvement. Addressing these issues is essential before the manuscript can be reconsidered for publication.

[Note: HTML markup is below. Please do not edit.]

Reviewers' comments:

Reviewer's Responses to Questions

**Comments to the Author**

1. Is the manuscript technically sound, and do the data support the conclusions?

Reviewer #1: Yes

Reviewer #2: Partly

Reviewer #3: Partly

Reviewer #4: Yes

Reviewer #5: Yes

Reviewer #6: Yes

Reviewer #7: Yes

Reviewer #8: Partly

Reviewer #9: Partly

Reviewer #10: Yes

**********

2. Has the statistical analysis been performed appropriately and rigorously?

Reviewer #1: Yes

Reviewer #2: Yes

Reviewer #3: N/A

Reviewer #4: Yes

Reviewer #5: Yes

Reviewer #6: Yes

Reviewer #7: N/A

Reviewer #8: N/A

Reviewer #9: Yes

Reviewer #10: Yes

**********

3. Have the authors made all data underlying the findings in their manuscript fully available?

The PLOS Data policy

Reviewer #1: Yes

Reviewer #2: Yes

Reviewer #3: Yes

Reviewer #4: Yes

Reviewer #5: Yes

Reviewer #6: Yes

Reviewer #7: Yes

Reviewer #8: Yes

Reviewer #9: Yes

Reviewer #10: Yes

**********

4. Is the manuscript presented in an intelligible fashion and written in standard English?

Reviewer #1: Yes

Reviewer #2: Yes

Reviewer #3: No

Reviewer #4: Yes

Reviewer #5: Yes

Reviewer #6: Yes

Reviewer #7: Yes

Reviewer #8: Yes

Reviewer #9: Yes

Reviewer #10: Yes

**********

Reviewer #1: Major Points:

1-Antigenicity vs. Fixation Time: The study notes that full equilibration requires approximately 10 months. While the authors acknowledge that this period might cause some antigen masking, the manuscript would be strengthened by a more detailed discussion of which specific classes of biomolecules (e.g., lipid-anchored vs. cytosolic proteins) are most likely to be affected by this extended fluid storage prior to subzero stabilization.

2-Volumetric Analysis: The authors mention a 10% width increase in one donor (#180) and suggest it might be a measurement artifact. To meet PLOS ONE's standard for technical detail, please clarify if the measurement plane shift was confirmed by re-examining the 3D CT reconstructions.

Minor Points:

1-Species Generalizability: You used dog and pig brains for validation due to lower postmortem intervals. Explicitly state in the Discussion whether the denser human white matter might require even longer than the observed 10 months compared to the animal models used.

2-Study Limitations: In line with the preference for transparency, please ensure the limitation regarding the lack of biomolecular labeling (immunoreactivity) is highlighted as a primary area for future validation, as this is the main justification for the protocol.

Reviewer #2: 1- Very Small Sample Size

• 3 human brains

• 2 dog brains

Such small sample sizes limit the statistical power and generalizability of the findings.

2. Lack of Long-Term Storage Data

Although the method is proposed for long-term preservation, the freezer storage experiment lasted only two months. This is insufficient to demonstrate the long-term stability claimed in the discussion.

3. Limited Molecular Validation

The study evaluates morphological preservation, but does not test the primary motivation for the protocol: preservation of antigenicity or molecular markers.

Experiments such as:

• immunohistochemistry

• RNA preservation analysis

• protein stability assays

would significantly strengthen the study.

4. Mixed Species Samples

The experiments include:human brains, pig brains, dog brains

Species differences in tissue composition may influence cryoprotectant diffusion and ultrastructural preservation. This may reduce the generalizability of the results.

Reviewer #3: While the language used is grammatically correct, ambiguity and obfuscation by other means can easily mislead an average reader. As a result the aim of the paper methods and results of the paper are all unclear. Here are some examples:

1) The full title should reflect the content, so it would logically be something like, "A method for preventing freezing damage in aldehyde fixed brains". Instead "Aldehyde-based cryopreservation of whole brains" is more easily interpreted as, using Aldehyde to cryopreserve brains.

2) After the abstracts introductory sentence one might expect the aim of the experiment in the second sentence "This has the potential to maintain morphology for many decades, but has been found to cause progressive loss of antigenicity over time for some biomolecules." While this problem is extensively addressed in the paper's introduction and references, no attempt in the papers methods or results is made to address it. In fact the discussion says near the end "Fourth, we assessed only morphological preservation and did not evaluate biomolecular labeling outcomes such as immunoreactivity, which is the primary rationale for this approach over conventional room temperature or refrigerator storage." So why is it so prominent in the abstract and introduction?

3) Over half the paper's introduction has emphasis on preservation of immunoreactivity as well as structure. It is not until the last portion it clarifies the two "open questions" the paper is attempting to address. "One question is the extent to which any given protocol for immersion cryoprotection may cause structural alterations as visualized by microscopy.". and "how long it takes for the solution to diffuse into the brain tissue, which determines how long of a waiting period is necessary before moving the brain to subzero storage.

4) The question posed in the introduction regarding structural preservation of the brain when certain cryprotectants are added is poorly addressed in the methods. While the blind review of micrographs is reasonably addressed, the basics of making the concentrations of cryoprotecants themselves known is poorly done. The table of concentrations should stand alone as a recipe, but using w/w and v/v without a clear indication of order or total volumes leaves it not reproducible. Even when the supporting methods text is incorporated it is still not clear. Line 166 When was glutaraldehyde introduced previously in the loading steps?Line 176 On day 9, samples were transferred to the final solution: 50% ethylene glycol (v/v), . Is this still in 10% NBF? And more fundamentally, what was the volume of the bath of cryoprotectant solutions relative to the volume of the brain? Was there any attempt to prevent the bath solution becoming diluted during the course of the experiment?

5) The use of CT scanning to track the penetration of the cryoprotectants was worth a try. Stating in the abstract "We used CT imaging to track cryoprotectant penetration, finding that with the use of our protocol, approximately 10 months is

required to reach equilibration throughout whole human brains." , This totally misleading when contrasted with lines 479-486 in the discusion which indicate CT scans can't resolve the differential penetration rates of the cryoprotectants.

Overall I find the paper is to imprecise in aims, methodolgy and results to be substantially useful.

Reviewer #4: 1. TITLE & ABSTRACT

• Title is clear, specific, and publishable

• Abstract is well-structured and informative

• Includes:

o Problem

o Method

o Key result (10 months equilibration)

o Validation (histology + EM)

Needs Revision

1. Too long and dense (typical PLOS ONE prefers slightly tighter abstracts)

2. Some sentences are overloaded with technical detail

3. “which is one type of a more general approach…” → wordy

4. Results could be more quantitatively emphasized

Suggested Improved Abstract

Long-term storage of aldehyde-fixed brain tissue in fluid preserves morphology but can lead to progressive loss of antigenicity. While cryoprotection has been widely used for tissue sections, its application to whole brains remains under-characterized.

Here, we present an aldehyde-based cryopreservation (ABC) protocol for intact brains using graded immersion in cryoprotectants followed by subzero storage. The protocol employs stepwise osmotic loading to a final solution containing 50% ethylene glycol and 30% sucrose.

Using CT imaging, we demonstrate that whole human brains require approximately 10 months to achieve full cryoprotectant equilibration. Insufficient equilibration resulted in ice crystal artifacts in white matter, while optimized protocols preserved cellular architecture at both light and electron microscopy levels.

This method enables long-term storage using standard laboratory freezers and offers resilience to freezer failure due to continued preservation in fluid state. The ABC approach may improve long-term antigenicity preservation in large brain specimens for research applications.

2. KEYWORDS

• Relevant and appropriate but Add: Ultrastructure, Electron microscopy, Tissue preservation

3. INTRODUCTION

• Excellent problem framing

• Clear gap identification

• Strong logical flow

• Good literature integration

Needs Revision

a. Repetition

• “irreplacable specimen” idea repeated → compress

b. Some sentences too long

Example:

“Each banked brain is an irreplaceable specimen…”

→ Could be tightened

c. Aim statement slightly buried

• Needs a clear final objective sentence

Suggested Improved Ending Paragraph

To address these gaps, we developed and evaluated a protocol for whole-brain cryoprotectant immersion following aldehyde fixation. Using CT imaging, we quantified cryoprotectant diffusion kinetics in intact human brains. Additionally, we performed histological validation to determine whether cryoprotectant loading, storage, and unloading introduce structural artifacts. Together, these analyses provide a practical framework for long-term preservation of whole brain specimens.

4. MATERIALS & METHODS

• Very detailed and reproducible

• Strong experimental transparency

• Includes: Reagents, Protocol steps, Imaging, Statistics

Needs Revision

a. Structure

• Sections are too dense

• Improve readability by:

o Adding subheadings

o Shortening paragraphs

b. Ethics Statement

• Good, but should match journal format exactly

Suggested Improvement (Ethics – more formal)

All animal tissues were obtained postmortem from animals euthanized for reasons unrelated to this study, with owner consent where applicable. No live animals were used; therefore, IACUC approval was not required. Human body donations were conducted under Oregon Health Authority regulations. The Apex Neuroscience Brain and Tissue Bank operates under an exemption determination from the Pearl Institutional Review Board (IRB ID #2023-0260).

5. RESULTS

• Logical structure:

o Solution → kinetics → validation

• Strong use of:

o CT imaging

o Histology

• Excellent cause-effect reasoning

6. DISCUSSION

• Very strong section overall

• Deep Mechanistic reasoning and Literature integration

• Addresses well the Protocol rationale, Diffusion, Cryoprotectants

Needs Revision

a. Too long (major issue)

• Needs ~20–25% reduction

b. Redundancy

• Cryoprotectant explanation repeated

• It’s strongly suggested to cite following most relevant studies:

� https://rootspress.org/journals/agribiol/article/view/1176

� https://europepmc.org/article/ppr/ppr493066

c. Some claims too strong

Example:

“will maintain for many decades”

d. Improved Version

“is expected to support long-term preservation, potentially for decades”

7. LIMITATIONS

• Honest and well-written

• Covers:

o Sample size

o Species differences

o Missing molecular validation

8. CONCLUSION

• Clear summary

• Matches results

Good Luck

Reviewer #5: This study addresses the critical challenge of cryopreserving whole aldehyde-fixed brains. It is the first to systematically characterize the penetration kinetics of cryoprotective agents (CPAs) in intact human brains and resolve the core issue of ice crystal damage by optimizing the experimental protocol. The developed Aldehyde-based cryopreservation (ABC) method has the potential to preserve both tissue morphology and antigenicity, providing a novel and feasible solution for the long-term preservation of large-volume brain tissue in brain banks. The research topic is highly relevant to the practical needs of neuroscience research, with innovative and practical methodological design, relatively complete experimental data, and generally reliable conclusions. However, the study has notable limitations including small sample sizes, insufficient mechanistic exploration, and inadequate verification of practical application efficacy, which require further supplementation and improvement.

Reviewer #6: This manuscript presents a practical aldehyde‑based cryopreservation (ABC) protocol for fixed whole brains, combining graded cryoprotection and −20 °C storage. The authors use CT imaging to demonstrate that full equilibration in human brains takes approximately 10 months, with slower diffusion in white matter. Histological and ultrastructural analyses show that the refined protocol eliminates ice crystal artifacts and preserves tissue architecture comparably to controls. The method is simple, uses standard laboratory equipment, and offers resilience against freezer failure, making it highly valuable for brain banking. Minor revisions are needed to clarify solution composition, standardize figure labeling, and strengthen descriptions of protocol modifications. Overall, this is a rigorous, well‑designed study with clear translational impact; it is suitable for publication after these small adjustments.

Reviewer #7: The manuscript "Aldehyde-based cryopreservation of whole brains" discusses the preservation of whole brains combining aldehyde-based fixation with graded immersion cryoprotection and subzero temperature storage to prevent loss of antigenicity over time for some biomolecules overtime. The paper is well written and the limitations of the study are clearly stated. To enable a thorough evaluation of the manuscript, it is essential that the authors provide data supporting their hypothesis of antigenicity preservation using this novel method. Without this data it is unclear how the integrated protocol supersedes current aldehyde based fixation protocols.

Reviewer #8: Revision: PONE-D-26-10146. Aldehyde-based cryopreservation of whole brains

This manuscript by Garrood and others addresses very valuable topic; the need for suitable methods of preservation of precious samples such as brains for diagnostic purposes, however, several aspects of the study design, data presentation, and overall structure limit its impact and clarity. Some sections of the manuscript would benefit from reorganization. For example, parts of the discussion are overly long and could be streamlined or partially moved to the results section to improve clarity and flow. The work doesn't seem to follow the structure of a hypothesis-driven research study. Instead, it reads more as an observational or descriptive report. While the topic is of potential interest, the manuscript would benefit from substantial revisions to improve its scientific rigor, clarity, and structure. Strengthening the statistical analysis, clarifying the study design, and improving data presentation will be essential for supporting the conclusions. Below are my main comments and suggestions for improvement.

Major Comments

1. Introduction and Rationale

The introduction does not clearly articulate the need for improving preservation methods for brain to sufficiently justify the study. The authors should better define the knowledge gap and explain why this work is necessary in the context of existing literature, and why this work is novel. I wonder if this method would also be applicable to preservation of other whole organs or tissues.

2. Sample Size and Statistical Analysis

The small sample size (n = 3) is insufficient to support strong conclusions, particularly regarding volumetric changes in human brains (e.g., line 279 onward). With only one out of three samples showing changes, it is difficult to determine whether this reflects biological variability, measurement error, or an effect of the preservation method.

Additionally, there is a lack of statistical analysis for most measurements, which further limits interpretability. The only section that includes statistical treatment is the preservation score analysis of six samples at the end of the paper.

3. Presentation of Results vs. Preliminary Observations

The initial results section (e.g., browning of brains due to glycerol) lacks supporting images and appears more like preliminary or observational data. This material may be more appropriate for inclusion in the introduction rather than the results section.

Similarly, the choice of cryoprotective solution should be justified upfront in the introduction rather than presented as a primary result since there is no experimental data for this.

4. Quantification of Observations

Several observations are described qualitatively. The manuscript would benefit from quantitative analyses wherever possible to strengthen the conclusions.

Minor Comments

1. Figures and Tables

o The overall quality of the figures, particularly H&E figure is very low and should be improved.

o Table 1 may be more appropriate as supplementary material. Additionally, the legend refers to “MW,” which is not used or explained in the table.

Reviewer #9: This manuscript describes an aldehyde‑based cryopreservation (ABC) workflow for whole brains, combining graded immersion in cryoprotectants and storage at −20 °C. The protocol culminates in a solution of 50% (v/v) ethylene glycol and 30% (w/v) sucrose (with adjustments regarding PVP and fixatives across stages). The first part quantifies diffusion kinetics in intact human brains by CT, reporting ~10 months to achieve equilibration and noting substantially slower penetration in white matter compared with gray matter. The second part evaluates histological and ultrastructural integrity on canine brain biopsies, contrasting an initial protocol that generated white‑matter artifacts (consistent with ice), with a refined protocol that eliminated those artifacts; blinded EM ratings showed no significant difference between cryopreserved and control samples (ICC = 0.76; Wilcoxon p = 0.30). The authors argue this approach can be implemented in standard freezers and may better preserve antigenicity long‑term than fluid storage at higher temperatures.

Overall, the work addresses a practical and under‑documented challenge in brain banking. The study is thoughtfully designed, the limitations are candidly acknowledged, and the discussion is well‑informed. The principal weaknesses are (i) limited biological validation of the claimed antigenicity benefit (no immunohistochemistry is provided), (ii) the feasibility of a ~10‑month equilibration for routine human brain banking, (iii) species mismatch between human (CT kinetics only) and dog (ultrastructural validation), and (iv) relatively coarse volumetric assessment during CT monitoring. These issues are addressable. I recommend Major Revision.

---

Major Comments

M1. Practical feasibility and optimization of diffusion timeline / The CT data in human whole brains indicate ~10 months are required to reach equilibration in the final solution, with notably slower diffusion in white matter. While compelling scientifically, this timeline is burdensome operationally for brain banks. Please (i) expand the discussion of practical strategies to accelerate equilibration (e.g., mild elevation of temperature with biochemical risk controls, solution agitation/recirculation, intermittent partial dissection, vascular pathway facilitation post‑fixation), (ii) provide estimates or pilot data on temperature‑dependence for diffusion to bound plausible timelines, and (iii) clarify whether partial equilibration suffices for −20 °C storage given that ethylene glycol alone (at the stated level) may preclude ice formation before sucrose fully equilibrates.

M2. Biological validation of antigen preservation / The study’s stated long‑term value proposition is enhanced antigenicity retention relative to conventional fluid storage, yet no immunohistochemical (IHC) assays are reported. Given PLOS ONE’s focus on methodological soundness, it would substantially strengthen the manuscript to add a minimal IHC panel (e.g., NeuN, GFAP, Iba1, synaptophysin) comparing cryopreserved vs. fluid‑stored controls after representative storage intervals; if currently infeasible, provide a clear rationale and a concrete plan for future validation, and temper claims accordingly.

M3. Species and tissue‑state differences / Human brains were used for CT diffusion kinetics, whereas ultrastructural validation was performed on canine biopsies with shorter PMIs and potentially distinct extracellular architectures. Please expand on the limitations in extrapolating canine biopsy outcomes to whole human brains, especially regarding white‑matter packed myelinated fibers and postmortem changes that may affect permeability/osmotic responses. Consider explicitly stating that ultrastructural preservation in whole human brains remains to be demonstrated.

M4. Volumetric change assessment / The manuscript reports a single linear width measure on serial CT slices and acknowledges its limitations, including a plausible landmark shift artifact. More robust volumetric or surface‑based metrics (even retrospectively from existing CT datasets) would be preferable; if not possible, please strengthen the limitations and suggest concrete methods for future work (e.g., 3D segmentation, fiducial‑based registration, serial photography with scale).

M5. Freezer‑failure resilience claim / The manuscript asserts that morphology would remain preserved in the fluid state upon rewarming after a freezer failure. Please qualify this claim with explicit time‑at‑temperature tolerances (based on prior literature or internal experience) and clarify which biomolecular features are most vulnerable during any such excursions.

M6. Statistical power and inference / The image‑level ratings were appropriately collapsed to the sample level (mean of two blinded raters per image, then averaged per specimen) and compared between groups using a two‑sided Wilcoxon rank‑sum test, with inter‑rater reliability quantified via ICC(2,1)—an analysis plan that is methodologically sound. However, with n = 6 specimens per group, the study is underpowered to detect modest or even moderate differences in ultrastructural quality. Using the observed dispersion of the sample‑level means (pooled SD ≈ 0.62), Monte Carlo power analyses indicate that ≈26 samples per group would be needed to achieve ~80% power for a Δ = 0.5‑point shift on the 1–5 scale, whereas the current design only reaches adequate power for very large effects (Δ ≥ 1.2). Thus, the non‑significant result (p ≈ 0.30) should not be interpreted as evidence of equivalence between conditions; rather, it reflects insufficient sensitivity to rule out practically relevant effects. I recommend (i) explicitly stating this limitation, (ii) reporting effect sizes with confidence intervals at the sample level (e.g., Hodges–Lehmann shift, AUC for Mann–Whitney), and (iii) tempering claims regarding the absence of cryopreservation‑induced ultrastructural alterations. If the authors wish to exploit the full image‑level dataset in future work, a hierarchical mixed‑effects ordinal model (images nested within specimens; random intercepts for raters) could be considered, while retaining the specimen as the unit of inference to avoid pseudoreplication.

M7. Statistical analysis transparency / The EM image scoring is clearly described, with blinded raters and ICC reporting (0.76). Please specify: (i) exact N at the level of images and samples for each condition and region, (ii) any pre‑registration of the scoring rubric (if applicable), and (iii) provide per‑sample distributions (e.g., in a supplemental figure) to complement the Wilcoxon summary statistics.

M8. Citation quality and physical chemistry / Where the melting point or phase behavior of the 50% ethylene glycol solution is discussed, ensure the reference is peer‑reviewed or from a handbook‑grade source. If a non‑peer‑reviewed web resource is cited, please consider replacing or supplementing with more authoritative data and discuss the combined colligative effects of sucrose on the depression of the freezing point, acknowledging current uncertainties.

M9. Scope of the Discussion / The permafrost and mammoth DNA passages are interesting but somewhat speculative relative to the present data and could be condensed to keep focus on what is demonstrated here and on immediately testable extensions (e.g., −40 °C/−80 °C with alternative CPA mixtures, practical loading/unloading schedules, and IHC outcomes).

---

Minor Comments

- Terminology: Consistently define ABC early. A brief schematic of the workflow would improve readability.

- Protocol clarity: The refined biopsy protocol includes 2% glutaraldehyde during loading/unloading. Please clarify the rationale (osmotic stabilization vs. crosslink enhancement) and whether similar inclusion is advisable or feasible for whole brains.

- Figure legends: Ensure every EM panel includes scale bars in the legend and that stain/en bloc contrasting steps are unambiguously mapped to figures; the text is clear, but the figure legends could be more self‑contained.

- White‑matter artifacts, initial protocol: It would help to quantify the frequency/area fraction of voids in affected white matter (even semi‑quantitatively) to support the qualitative interpretation of ice‑like morphology.

- Data availability: The manuscript references Zenodo (WSI and EM) and GitHub (code). Please provide direct DOIs and specific repository record links (not only a community URL) and ensure that raw/derived data and analysis scripts are sufficient for full reproduction of statistics and figures.

- COI transparency: The ethics statement and IRB exemption are clearly stated; the competing interests note employment at a brain preservation organization. Consider adding a brief sentence in the Discussion affirming how study design, data analysis, and interpretation were insulated from these interests.

- Limitations section: Already strong; consider adding a succinct note that human whole‑brain ultrastructural validation (post‑ABC) is a key next step.

---

Point‑by‑Point Questions for the Authors

1. Equilibration feasibility: Can you provide quantitative estimates or pilot data on how modest temperature increases (e.g., 4 °C → 10 °C) or controlled agitation/recirculation would alter diffusion times for ethylene glycol and sucrose, and whether such changes introduce detectable morphological risks?

2. Partial equilibration tolerance: Given that 50% ethylene glycol alone may suffice to avoid ice at −20 °C, is full sucrose equilibration necessary before freezer transfer? Could you propose decision criteria (e.g., CT thresholds) for safe transfer under partial sucrose penetration?

3. Human applicability: Do you have any preliminary biopsy‑level EM from human brains after full ABC (loading → −20 °C storage → unloading) to corroborate canine findings? If not, please outline a concrete plan or acknowledge this as a priority limitation.

4. IHC validation: Can you add a minimal IHC panel (NeuN, GFAP, Iba1, synaptophysin or equivalent) comparing ABC vs. conventional fluid storage after a defined interval? If not, please justify and temper claims regarding antigen preservation.

5. Statistical Power: Given the small sample size (n=6), how do you justify the claim of "no significant difference" as evidence of ultrastructural preservation rather than a lack of statistical power?

6. Unloading steps: The refined protocol shows a gradual 18‑day unloading with 2% glutaraldehyde. Could you provide reasoning and any comparative data supporting the chosen decrement schedule (and whether this regimen is scalable to whole brains)?

7. White‑ vs. gray‑matter kinetics: Beyond qualitative CT observations, can you estimate effective diffusion coefficients (or relative rates) for white vs. gray matter, even if approximate, to aid protocol planning?

8. Volumetric assessment: Is it possible to perform retrospective 3D segmentation on existing CT datasets to provide volumetric change estimates (or, minimally, add orthogonal linear measurements and show their consistency)?

9. Freezer‑failure resilience: Please specify time‑temperature excursion limits under which morphology and antigenicity remain robust, and indicate any corrective actions (e.g., solution replacement) recommended after such events.

10. Solution composition and references: For the phase behavior of the final solution, can you provide a peer‑reviewed or handbook source and discuss the combined colligative effects of sucrose with ethylene glycol (and implications for −40/−80 °C)?

11. Data and code access: Please supply permanent DOIs (not only a community page) for Zenodo datasets and ensure the GitHub repository contains scripts/notebooks sufficient to reproduce figures and statistics (including ICC and Wilcoxon analyses).

12. COI safeguards: Beyond disclosure, were any safeguards in place (e.g., independent data analysis, blinded figure selection) to mitigate bias given the authors’ affiliations with a brain preservation organization? A brief description would enhance transparency

Reviewer #10: The paper demonstrates the optimal time of submersion in cryoprotectant solution for the adequate biobanking of whole brains, which is essential to ensure the validity of cryopreservation protocols and will surely serve as base for future research in the area. The results provided form a important stepping stone to address future refinement in cryopreservation of whole brains, with possible alternative protocols to wield better results. Even though the solid results, it is important to take a conservative view in extrapolating the results, better highlighting if the results derive from the human or dog experiments, with cautious generalization to all brains. Since the aim of the study is to present a protocol for broad usage, some minor revisions are necessary for better understanding of the procedures, as well as some methodological questions that might be valid to include in the discussion. For the questions, if the changes applied were designed to refine the protocol based on previous results and not just due to laboratory routines, it could be relevant to explicit discuss that, emphasizing the refinement conducted along the study. Finally, some suggestions are made as final comments regarding the figures that do not impact directly in the manuscript itself but may help to improve the interpretation of the results for future readers.

Minor revisions

- Include glutaraldehyde and formalin in some of the descriptions of the final solution, specially because is a modification in comparison to more traditional cryopreservation solutions described in the literature.

- In figure 3, review the figure legend regarding which images are form the cryopreserved or non-cryopreserved group.

- In the discussion, it said that the PVP was removed in the final protocol, which is clearly demonstrated in the methodology for the processing of the 177 donor. But in the methodology for the humans brains, the PVP is still present, making it rather unclear if it was used or not in the human brain cryopreservation. Since the main result in a protocol intended for human usage is important to specify if the removal of PVP was done only for the 177 donor, or for all the human donors as well.

- In line 537, DMSO is directly cited with an abbreviation, without the full description before, and is also absent in the abbreviations list.

Questions

- Why was PBS used in the refined protocol for the second dog brain instead of only NBF like in the previous solutions?

- Why was the electron microscopy processing modified?

- Why was the ethylene glycol the only cryoprotectant that had a stepwise increasing concentration? Why was not the sucrose also increased in a stepwise way?

- In the results, the authors say that the human brains were easily able to be sectioned for extracting a biopsy sample, was any tissue extracted? If so, was it submitted to any histological evaluation like the procedures done to the dogs brains? If not, it may be important to include this lack of analysis in the limitation section.

- Regarding the volumetric change observed in one of the human brain, is there any medical condition from the donor that might contribute to this alteration?

Suggestions

- In figure 1, add the solution change in each ramp up, to a better visualisation of the stepping protocol.

- In figure 3, add a title above the panels to indicate the groups, similar to the previous figures.

- In figures 2 and 4, add the donor number in the figure legend.

- If possible, adding images of the browning of the pig and human brains in the supplementary material may be interesting for visualisation of the process.

**********

what does this mean?). If published, this will include your full peer review and any attached files.

If you choose “no”, your identity will remain anonymous but your review may still be made public.

**Do you want your identity to be public for this peer review?**  For information about this choice, including consent withdrawal, please see our Privacy Policy

Reviewer #1: **Yes:** Haider Abbas Hadi Al-Mhanaa

Reviewer #2: No

Reviewer #3: **Yes:** W.T.M Gruijters

Reviewer #4: **Yes:** Dr. Asif Bilal

Reviewer #5: No

Reviewer #6: No

Reviewer #7: No

Reviewer #8: No

Reviewer #9: **Yes:** Pr. Samuel Buff

Reviewer #10: No

**********

[NOTE: If reviewer comments were submitted as an attachment file, they will be attached to this email and accessible via the submission site. Please log into your account, locate the manuscript record, and check for the action link "View Attachments". If this link does not appear, there are no attachment files.]

To ensure your figures meet our technical requirements, please review our figure guidelines: https://journals.plos.org/plosone/s/figures

You may also use PLOS’s free figure tool, NAAS, to help you prepare publication quality figures: https://journals.plos.org/plosone/s/figures#loc-tools-for-figure-preparation.

NAAS will assess whether your figures meet our technical requirements by comparing each figure against our figure specifications.

Attachment Submitted filename: plos.docx

Attachment Submitted filename: PONE REVISION.docx

Attachment Submitted filename: renamed_6c556.docx

# Author comment: Author response to Decision Letter 1

**DOI:** 10.1371/journal.pone.0344932.r002

4 Jun 2026

Please see our attached document, "Response to Reviewers".

Attachment Submitted filename: Response to Reviewers.pdf

# Peer review: Decision Letter 1

**DOI:** 10.1371/journal.pone.0344932.r003

**Authors:** Ghadeer Sabah Bustani

15 Jul 2026

Dear Dr. McKenzie,

Thank you for submitting your manuscript to PLOS ONE. After careful consideration, we feel that it has merit but does not fully meet PLOS ONE’s publication criteria as it currently stands. Therefore, we invite you to submit a revised version of the manuscript that addresses the points raised during the review process.

The manuscript is suitable for publication after addressing the remaining minor issues identified by the reviewer. Please revise the manuscript carefully in accordance with the reviewer’s recommendations, particularly regarding the revising the title to ensure that it is scientifically accurate, descriptive, concise, and not structured primarily to accommodate the “ABC” acronym. The title should clearly distinguish between cryopreservation of aldehyde-fixed brains and aldehyde-based cryopreservation.

Please submit your revised manuscript by Aug 29 2026 11:59PM. If you will need more time than this to complete your revisions, please reply to this message or contact the journal office at plosone@plos.org. When you're ready to submit your revision, log on to https://www.editorialmanager.com/pone/ and select the 'Submissions Needing Revision' folder to locate your manuscript file.

- A letter that responds to each point raised by the academic editor and reviewer(s). You should upload this letter as a separate file labeled 'Response to Reviewers'.
- A marked-up copy of your manuscript that highlights changes made to the original version. You should upload this as a separate file labeled 'Revised Manuscript with Track Changes'.
- An unmarked version of your revised paper without tracked changes. You should upload this as a separate file labeled 'Manuscript'.

If you would like to make changes to your financial disclosure, please include your updated statement in your cover letter. Guidelines for resubmitting your figure files are available below the reviewer comments at the end of this letter.

If applicable, we recommend that you deposit your laboratory protocols in protocols.io to enhance the reproducibility of your results. Protocols.io assigns your protocol its own identifier (DOI) so that it can be cited independently in the future. For instructions see: https://journals.plos.org/plosone/s/submission-guidelines#loc-laboratory-protocols. Additionally, PLOS ONE offers an option for publishing peer-reviewed Lab Protocol articles, which describe protocols hosted on protocols.io. Read more information on sharing protocols at https://plos.org/protocols?utm_medium=editorial-email&utm_source=authorletters&utm_campaign=protocols.

As the corresponding author, your ORCID iD is verified in the submission system and will appear in the published article. PLOS supports the use of ORCID, and we encourage all coauthors to register for an ORCID iD and use it as well. Please encourage your coauthors to verify their ORCID iD within the submission system before final acceptance, as unverified ORCID iDs will not appear in the published article. *Only*  the individual author can complete the verification step; PLOS staff *cannot*  verify ORCID iDs on behalf of authors.

We look forward to receiving your revised manuscript.

Kind regards,

Ghadeer Sabah Bustani, Ph.D

Academic Editor

PLOS One

Journal Requirements:

1. If the reviewer comments include a recommendation to cite specific previously published works, please review and evaluate these publications to determine whether they are relevant and should be cited. There is no requirement to cite these works unless the editor has indicated otherwise.

2. Please review your reference list to ensure that it is complete and correct. If you have cited papers that have been retracted, please include the rationale for doing so in the manuscript text, or remove these references and replace them with relevant current references. Any changes to the reference list should be mentioned in the rebuttal letter that accompanies your revised manuscript. If you need to cite a retracted article, indicate the article’s retracted status in the References list and also include a citation and full reference for the retraction notice.

[Note: HTML markup is below. Please do not edit.]

Reviewers' comments:

Reviewer's Responses to Questions

**Comments to the Author**

Reviewer #3: (No Response)

Reviewer #4: (No Response)

Reviewer #7: All comments have been addressed

**********

2. Is the manuscript technically sound, and do the data support the conclusions?

Reviewer #3: Partly

Reviewer #4: Yes

Reviewer #7: Yes

**********

3. Has the statistical analysis been performed appropriately and rigorously?

Reviewer #3: Yes

Reviewer #4: N/A

Reviewer #7: N/A

**********

4. Have the authors made all data underlying the findings in their manuscript fully available?

The PLOS Data policy

Reviewer #3: Yes

Reviewer #4: Yes

Reviewer #7: Yes

**********

5. Is the manuscript presented in an intelligible fashion and written in standard English?

Reviewer #3: No

Reviewer #4: Yes

Reviewer #7: Yes

**********

Reviewer #3: The authors have improved their manuscript considerably. Some might be interested to know how long it takes for cryo-protectants to penetrate fixed brain, even if the paper in only indicative rather than precise about the process.

There are still two major problems already raised that have not been addressed at all or not fully addressed.

1)The title is still aimed at being an expanded acronym rather than descriptive of the paper’s content. As much as ABC for Aldehyde-based cryopreservation .... might be useful as a short cut descriptor in a lab it does not appropriately reflect the contents of the paper. There is no “Aldehyde-based cryopreservation” of whole brains in the paper. There is cryopreservation of aldehyde fixed brains, a significantly different meaning. To sacrifice the meaning of the paper’s title for the sake of fitting an illogical acronym is more than inconcise. It is significantly flawed paper writing style. Running against Plone’s requirements for a title ‘Titles must be specific, descriptive, concise, and understandable to readers outside your exact field’.

2)While the formulae used for cryopreservatives have been enourmously improved they still appear incomplete and technically non-reproducible without assumptions being made. Around line 198 Formula in the text and table 2 seem to assume incorrectly that 30% sucrose w/v adds no volume to the cryopreservatives? Table 2 40%+ 30%+ 28%+ 2%= 100% plus 25% sucrose =125% for example? While not directly proportional it does add at least 13% to the volume. How is this accounted for?

A minor point. In terms of clarity of the somewhat lengthy sentences at times, one that seems worth revising:

183 "The substitution of PBS for most of the NBF was made to reduce the total formaldehyde load through the loading, storage, and unloading process, which we judged to be unnecessary at the original concentration once 2% glutaraldehyde was added earlier and maintained throughout the solution changes."

The sentence so long it becomes confusing. The subject of the sentence is changed without making it clear which subjects are being referred to at any one point. I presume it means :

The substitution of PBS for most of the NBF was made to reduce the total formaldehyde load through the loading, storage, and unloading processes. We judged formaldehyde unnecessary at the original concentration due to the effect of adding 2% glutaraldehyde earlier in this protocol than in other protocols, and maintaining glutaraldehyde throughout the solution changes.

Reviewer #4: he authors present an interesting and well-executed study describing an aldehyde-based cryopreservation protocol for whole-brain preservation following aldehyde fixation. The manuscript addresses an important methodological challenge in neuroscience, brain banking, and tissue preservation by proposing a practical approach that combines conventional aldehyde fixation with cryoprotectant loading and subzero storage. The study is well organized, the objectives are clearly stated, and the methodology is described in sufficient detail to facilitate reproducibility. The use of CT imaging to monitor cryoprotectant penetration, together with light microscopy and electron microscopy for structural validation, provides a comprehensive evaluation of the proposed protocol. Overall, the work represents a valuable contribution to the field and has the potential to benefit laboratories engaged in long-term preservation of whole-brain specimens.

Reviewer #7: (No Response)

**********

what does this mean?). If published, this will include your full peer review and any attached files.

If you choose “no”, your identity will remain anonymous but your review may still be made public.

**Do you want your identity to be public for this peer review?**  For information about this choice, including consent withdrawal, please see our Privacy Policy

Reviewer #3: **Yes:** W.T.M. Gruijters

Reviewer #4: **Yes:** Dr. Asif Bilal

Reviewer #7: No

**********

[NOTE: If reviewer comments were submitted as an attachment file, they will be attached to this email and accessible via the submission site. Please log into your account, locate the manuscript record, and check for the action link "View Attachments". If this link does not appear, there are no attachment files.]

To ensure your figures meet our technical requirements, please review our figure guidelines: https://journals.plos.org/plosone/s/figures

You may also use PLOS’s free figure tool, NAAS, to help you prepare publication quality figures: https://journals.plos.org/plosone/s/figures#loc-tools-for-figure-preparation.

NAAS will assess whether your figures meet our technical requirements by comparing each figure against our figure specifications.

# Author comment: Author response to Decision Letter 2

**DOI:** 10.1371/journal.pone.0344932.r004

27 Jul 2026

Response to Reviewers

We thank the Editor and the Reviewers for their positive and constructive comments, which have greatly improved the manuscript. Below, we provide point-by-point responses to the comments from Reviewer #3.

> The authors have improved their manuscript considerably. Some might be interested to know how long it takes for cryo-protectants to penetrate fixed brain, even if the paper in only indicative rather than precise about the process.

There are still two major problems already raised that have not been addressed at all or not fully addressed.

1)The title is still aimed at being an expanded acronym rather than descriptive of the paper’s content. As much as ABC for Aldehyde-based cryopreservation .... might be useful as a short cut descriptor in a lab it does not appropriately reflect the contents of the paper. There is no “Aldehyde-based cryopreservation” of whole brains in the paper. There is cryopreservation of aldehyde fixed brains, a significantly different meaning. To sacrifice the meaning of the paper’s title for the sake of fitting an illogical acronym is more than inconcise. It is significantly flawed paper writing style. Running against Plone’s requirements for a title ‘Titles must be specific, descriptive, concise, and understandable to readers outside your exact field’.

We thank the reviewer for this comment. We agree with the reviewer. To make the title more accurately reflect the contents of the paper, we have changed the title to “Cryopreservation of aldehyde-fixed whole brains”.

> 2)While the formulae used for cryopreservatives have been enourmously improved they still appear incomplete and technically non-reproducible without assumptions being made. Around line 198 Formula in the text and table 2 seem to assume incorrectly that 30% sucrose w/v adds no volume to the cryopreservatives? Table 2 40%+ 30%+ 28%+ 2%= 100% plus 25% sucrose =125% for example? While not directly proportional it does add at least 13% to the volume. How is this accounted for?

We thank the reviewer for this comment. This comment prompted us to revisit our original solution preparation records, which led us to identify four errors in the manuscript that have now been corrected.

First, for both the loading and unloading portions of the refined protocol, the volume contribution of dissolved sucrose was not accounted for during solution preparation. Because the final solution volume was not measured after sucrose dissolution, the exact final concentrations cannot be determined retrospectively. Therefore, we added clarifications throughout the Methods that the reported concentrations should be regarded as nominal concentrations based on the intended formulation rather than measured final solution volumes. For example, when describing the loading protocol we noted: “Notably, the additional volume contributed by the dissolved sucrose after adding it to PBS was not accounted for, so these percentages should be considered nominal concentrations, because they are based on the intended formulation rather than the measured final solution volume.” We also added a paragraph on this to the Limitations section: “Seventh, in the refined protocol for cryoprotectant loading and unloading of samples, the volume contribution of the dissolved sucrose in PBS was not accounted for when preparing the cryoprotectant solutions. As a result, the reported concentrations are nominal rather than true final concentrations, and the actual concentrations of the solution components were slightly lower than stated for those solutions.”

Second, we determined that for the whole brain protocol and the initial protocol of biopsy sample cryoprotection, the sucrose and PVP were dissolved in the ethylene glycol component of the solution rather than in NBF. This error has been corrected.

Third, we identified an error in the reported composition of the final cryoprotectant loading solution. The percentage of PBS was incorrectly listed as 28% rather than 38%. This error has been corrected.

Fourth, in reviewing our preparation records in response to the reviewer's comments, we discovered that the manuscript had incorrectly described the glutaraldehyde concentration. The protocol used 2% (v/v) of a 50% glutaraldehyde stock solution, corresponding to a final glutaraldehyde concentration of 1%, rather than 2%. This error has been corrected.

> A minor point. In terms of clarity of the somewhat lengthy sentences at times, one that seems worth revising:

183 "The substitution of PBS for most of the NBF was made to reduce the total formaldehyde load through the loading, storage, and unloading process, which we judged to be unnecessary at the original concentration once 2% glutaraldehyde was added earlier and maintained throughout the solution changes."

The sentence so long it becomes confusing. The subject of the sentence is changed without making it clear which subjects are being referred to at any one point. I presume it means :

The substitution of PBS for most of the NBF was made to reduce the total formaldehyde load through the loading, storage, and unloading processes. We judged formaldehyde unnecessary at the original concentration due to the effect of adding 2% glutaraldehyde earlier in this protocol than in other protocols, and maintaining glutaraldehyde throughout the solution changes.

We thank the reviewer for this comment. We have adopted the reviewer’s suggested revision for this sentence, which we agree makes it clearer.

Attachment Submitted filename: Response to Reviewers.docx

# Editor report: Decision Letter 2

**DOI:** 10.1371/journal.pone.0344932.r005

**Authors:** Ghadeer Sabah Bustani

2 Aug 2026

Cryopreservation of aldehyde-fixed whole brains

PONE-D-26-10146R2

Dear Dr. McKenzie,

We’re pleased to inform you that your manuscript has been judged scientifically suitable for publication and will be formally accepted for publication once it meets all outstanding technical requirements.

Within one week, you’ll receive an e-mail detailing the required amendments. When these have been addressed, you’ll receive a formal acceptance letter and your manuscript will be scheduled for publication.

An invoice will be generated when your article is formally accepted. Please note, if your institution has a publishing partnership with PLOS and your article meets the relevant criteria, all or part of your publication costs will be covered. Please make sure your user information is up-to-date by logging into Editorial Manager at Editorial Manager® and clicking the ‘Update My Information' link at the top of the page. For questions related to billing, please contact billing support.

If your institution or institutions have a press office, please notify them about your upcoming paper to help maximize its impact. If they’ll be preparing press materials, please inform our press team as soon as possible -- no later than 48 hours after receiving the formal acceptance. Your manuscript will remain under strict press embargo until 2 pm Eastern Time on the date of publication. For more information, please contact onepress@plos.org.

Kind regards,

Ghadeer Sabah Bustani, Ph.D

Academic Editor

PLOS One

Additional Editor Comments (optional):

Reviewers' comments:

# Editor report: Acceptance letter

**DOI:** 10.1371/journal.pone.0344932.r006

**Authors:** Ghadeer Sabah Bustani

PONE-D-26-10146R2

PLOS One

Dear Dr. McKenzie,

I'm pleased to inform you that your manuscript has been deemed suitable for publication in PLOS One. Congratulations! Your manuscript is now being handed over to our production team.

At this stage, our production department will prepare your paper for publication. This includes ensuring the following:

* All references, tables, and figures are properly cited

* All relevant supporting information is included in the manuscript submission,

* There are no issues that prevent the paper from being properly typeset

You will receive further instructions from the production team, including instructions on how to review your proof when it is ready. Please keep in mind that we are working through a large volume of accepted articles, so please give us a few days to review your paper and let you know the next and final steps.

Lastly, if your institution or institutions have a press office, please let them know about your upcoming paper now to help maximize its impact. If they'll be preparing press materials, please inform our press team within the next 48 hours. Your manuscript will remain under strict press embargo until 2 pm Eastern Time on the date of publication. For more information, please contact onepress@plos.org.

You will receive an invoice from PLOS for your publication fee after your manuscript has reached the completed accept phase. If you receive an email requesting payment before acceptance or for any other service, this may be a phishing scheme. Learn how to identify phishing emails and protect your accounts at https://explore.plos.org/phishing.

If we can help with anything else, please email us at customercare@plos.org.

Thank you for submitting your work to PLOS One and supporting open access.

Kind regards,

PLOS ONE Editorial Office Staff

on behalf of

Dr. Ghadeer Sabah Bustani

Academic Editor

PLOS One
